# Supplementary material for: CLAW: An automated Snakemake workflow for the assembly of chloroplast genomes from long-read data
Source: PLoS Comput Biol. 2024 Feb 9;20(2):e1011870. doi: 10.1371/journal.pcbi.1011870 (PMC10883564; doi:10.1371/journal.pcbi.1011870)
Supplement: S1 File — RbcL alignments from the algal, monocot, and dicot assemblies generated by CLAW and extracted from the reference genomes for each taxonomic group. (DOCX) [file pcbi.1011870.s001.docx]

Supplementary File 1 – RbcL alignments.

CLAW-generated *Flye* assemblies using ONT data

C. reinhardtii - reference ATGGTTCCACAAACAGAAACTAAAGCAGGTGCTGGATTCAAAGCCGGTGTAAAAGACTACCGTTTAACATACTACACACCTGATTACGTAGTAAGAGATACTGATATTTTAGCTGCATTCCGTATGACTCCACAACTAGGTGTTCCACCT 150

C. reinhardtii - CLAW ATGGTTCCACAAACAGAAACTAAAGCAGGTGCTGGATTCAAAGCCGGTGTA-AAGACTACCGTTTAACATACTACACACCTGATTACGTAGTAAGAGATACTGATATTTTAGCTGCATTCCGTATGACTCCACAACCAGGTGTTCCACCT 149

C. reinhardtii - reference GAAGAATGTGGTGCTGCTGTAGCTGCTGAATCTTCAACAGGTACATGGACTACAGTATGGACTGACGGTTTAACAAGTCTTGACCGTTACAAAGGTCGTTGTTACGATATCGAACCAGTTCCGGGTGAAGACAACCAATACATTGCTTAC 300

C. reinhardtii - CLAW GAAGAATGTGGTGCTGCTGTAGCTGCTGAATCTTCAACAGGTACATGGACTACAGTATGGACTGACGGTTTAACAAGTCTTGACCGTTACAAAGGTCGTTGTTACGATATCGAACCAGTTCCGGGTGAAGACAACCAATACATTGCTTAC 299

C. reinhardtii - reference GTAGCTTACCCAATCGACTTATTCGAAGAAGGTTCAGTAACTAACATGTTCACTTCTATTGTAGGTAACGTATTCGGTTTCAAAGCTTTACGTGCTCTACGTCTTGAAGACCTTCGTATTCCACCTGCTTACGTTAAAACATTCGTAGGT 450

C. reinhardtii - CLAW GTAGCTTACCCAATCGACTTATTCGAAGAAGGTTCAGTAACTAACATGTTCACTTCTATTGTAGGTAACGTATTCGGTTTCAAAGCTTTACGTGCTCTACGTCTTGAAGACCTTCGTATTCCACCTGCTTACGTTAAAACATTCGTAGGT 449

C. reinhardtii - reference CCTCCACACGGTATTCAGGTAGAACGTGACAAATTAAACAAATATGGTCGTGGTCTTTTAGGTTGTACAATCAAACCTAAATTAGGTCTTTCAGCTAAAAACTACGGTCGTGCAGTTTATGAATGTTTACGTGGTGGTCTTGACTTTACT 600

C. reinhardtii - CLAW CCTCCACACGGTATTCAGGTAGAACGTGACAAATTAAACAAATATGGTCGTGGTCTTTTAGGTTGTACAATCAAACCTAAATTAGGTCTTTCAGCTAAAAACTACGGTCGTGCAGTTTATGAATGTTTACGTGGTGGTCTTGACTTTACT 599

C. reinhardtii - reference AAAGACGACGAAAACGTAAACTCACAACCATTCATGCGTTGGCGTGACCGTTTCCTTTTCGTTGCTGAAGCTATTTACAAAGCTCAAGCAGAAACAGGTGAAGTTAAAGGTCACTACTTAAACGCTACTGCTGGTACTTGTGAAGAAATG 750

C. reinhardtii - CLAW AAAGACGACGAAAACGTAAACTCACAACCATTCATGCGTTGGCGTGACCGTTTCCTTTTCGTTGCTGAAGCTATTTACAAAGCTCAAGCAGAAACAGGTGAAGTTAAAGGTCACTACTTAAACGCTACTGCTGGTACTTGTGAAGAAATG 749

C. reinhardtii - reference ATGAAACGTGCAGTATGTGCTAAAGAATTAGGTGTACCTATTATTATGCACGACTACTTAACAGGTGGTTTCACAGCTAACACTTCATTAGCTATCTACTGTCGTGACAACGGTCTTCTTCTACACATCCACCGTGCTATGCACGCGGTT 900

C. reinhardtii - CLAW ATGAAACGTGCAGTATGTGCTAAAGAATTAGGTGTACCTATTATTATGCACGACTACTTAACAGGTGGTTTCACAGCCAACACTTCATTAGCTATCTACTGTCGTGACAACGGTCTTCTTCTACACATCCACCGTGCTATGCACGCGGTT 899

C. reinhardtii - reference ATTGACCGTCAACGTAACCACGGTATTCACTTCCGTGTTCTTGCTAAAGCTCTTCGTATGTCTGGTGGTGACCACCTTCACTCTGGTACTGTTGTAGGTAAACTAGAAGGTGAACGTGAAGTTACTCTAGGTTTCGTAGACTTAATGCGT 1050

C. reinhardtii - CLAW ATTGACCGTCAACGTAACCACGGTATTCACTTCCGTGTTCTTGCTAAAGCTCTTCGTATGTCTGGTGGTGACCACCTTCACTCTGGTACTGTTGTAGGTAAACTAGAAGGTGAACGTGAAGTTACTCTAGGTTTCGTAGACTTAATGCGT 1049

C. reinhardtii - reference GATGACTACGTTGAAAAAGACCGTAGCCGTGGTATTTACTTCACTCAAGACTGGTGTTCAATGCCAGGTGTTATGCCAGTTGCTTCAGGCGGTATTCACGTATGGCACATGCCAGCTTTAGTTGAAATCTTCGGTGATGACGCATGTCTT 1200

C. reinhardtii - CLAW GATGACTACGTTG-AAAAGACCGTAGCCGTGGTATTTACTTCACTCAAGACTGGTGTTCAATGCCAGGTGTTATGCCAGTTGCTTCAGGTGGTATTCACGTATGGCACATGCCAGCTTTAGTTGAAATCTTCGGTGATGACGCATGTCTT 1198

C. reinhardtii - reference CAGTTCGGTGGTGGTACTCTAGGTCACCCTTGGGGTAACGCTCCAGGTGCTGCAGCTAACCGTGTAGCTCTTGAAGCTTGTACTCAAGCTCGTAACGAAGGTCGTGACCTTGCTCGTGAAGGTGGCGACGTAATTCGTTCAGCTTGTAAA 1350

C. reinhardtii - CLAW CAGTTCGGTGGTGGTACTCTAGGTCACCCTTGGGGTAACGCTCCAGGTGCTGCAGCTAACCGTGTAGCTCTTGAAGCTTGTACTCAAGCTCGTAACGAAGGTCGTGACCTTGCTCGTGAAGGTGGCGACGTAATTCGTTCAGCTTGTAAA 1348

C. reinhardtii - reference TGGTCTCCAGAACTTGCTGCTGCATGTGAAGTTTGGAAAGAAATTAAATTCGAATTTGATACTATTGACAAACTTTAA 1428

C. reinhardtii - CLAW TGGTCTCCAGAACTTGCTGCTGCATGTGAAGTTTGGAAAGAAATTAAATTCGAATTTGATACTATTGACAAACTTTAA 1426

C. variabilis - CLAW ATGGCTCCACAAACTGAAACTAGAGCAGGTGCTGGGTTTAAAGCAGGTGTTAAAGACTACCGTTTAACTTACTATACTCCTGATTACCAACC-AAAGACACTGATATTCTTGCAGCATTCCGTATGACTCCTCAACCAGGTGTTCCACCA 149

C. variabilis - reference ATGGCTCCACAAACTGAAACTAGAGCAGGTGCTGGGTTTAAAGCAGGTGTTAAAGACTACCGTTTAACTTACTATACTCCTGATTACCAACCAAAAGACACTGATATTCTTGCAGCATTCCGTATGACTCCTCAACCAGGTGTTCCACCA 150

C. variabilis - CLAW GAAGAAGCTGGTGCAGCGGTAGCAGCAGAATCATCAACTGGTACTTGGACAACTGTATGGACTGATGGTTTAACTAGTTTAGATCGTTACAAAGGCCGTTGTTATGACATCGAGCCAGTTCCAGGTGAAGAAAACCAATACATTGCATAT 299

C. variabilis - reference GAAGAAGCTGGTGCAGCGGTAGCAGCAGAATCATCAACTGGTACTTGGACAACTGTATGGACTGATGGTTTAACTAGTTTAGATCGTTACAAAGGCCGTTGTTATGACATCGAGCCAGTTCCAGGTGAAGAAAACCAATACATTGCATAT 300

C. variabilis - CLAW ATTGCATATCCTTTAGATC-TTTTGAAGAAGGATCTGTAACTAATTTATTTACTTCAATTGTAGGTAACGTT-TTGGTTTCAAAGCTCTTCGTGCTTTACGTTTAGAAGATCTTCGTATTCCACCAGCATACGT-AAAACTTTCCAAGGT 446

C. variabilis - reference ATTGCATATCCTTTAGATCTTTTTGAAGAAGGATCTGTAACTAATTTATTTACTTCAATTGTAGGTAACGTTTTTGGTTTCAAAGCTCTTCGTGCTTTACGTTTAGAAGATCTTCGTATTCCACCAGCATACGTAAAAACTTTCCAAGGT 450

C. variabilis - CLAW CCTCCTCATGGTATTCAAGTAGAACGTGATAAACTTAACAAATATGGTCGTGGTTTATTAGGTTGTACAATTAAACCAAAATTAGGTCTTTCAGCT-AAAACTACGGTCGTGCTGTATACGAATGTTTACGTGGTGGTCTTGATTTCACT 595

C. variabilis - reference CCTCCTCATGGTATTCAAGTAGAACGTGATAAACTTAACAAATATGGTCGTGGTTTATTAGGTTGTACAATTAAACCAAAATTAGGTCTTTCAGCTAAAAACTACGGTCGTGCTGTATACGAATGTTTACGTGGTGGTCTTGATTTCACT 600

C. variabilis - CLAW AAAGATGATGAAAACGTAAACTCTCAACCATTCATGCGTTGGAGAGATCGTTTCTTATTCGTTGCGGAAGCTATCTACAAATCTCAATCTGAAACAGGTGAAATTAAAGGTCACTATTTAAATGCGACTGCAGCAACTGCTGAAG-AAAT 744

C. variabilis - reference AAAGATGATGAAAACGTAAACTCTCAACCATTCATGCGTTGGAAAGATCGTTTCTTATTCGTTGCGGAAGCTATCTACAAATCTCAATCTGAAACAGGGGAAATTAAAGGTCACTATTTAAATGCGACCGCACCA-CTGCTGGAGAAAAG 749

C. variabilis - CLAW GCTTAAACGTGCGGAATGTGCAAAAGATTTAGGTGTACCTATTGTTATGCATGACTACTTAACTGGTGGTTTCACAGCAAACACAAGTTTAGCTCATTACTGTCGTGATAATGGTCTTCTTCTA--CATTCACCGTGCAATGCACGCTGT 892

C. variabilis - reference GTTTAAACGTGCGGAATGTGCAAAAGATTTAGGTGTACCTATTGTTATGCATGACTACTTAACTGGTGGTTTCACAGCAAACACAAGTTTAGCTCATTACTGTCGTGATAATGGTCTTCTTCTACACATTCACCGGGCAATGCACGCGGT 899

C. variabilis - CLAW AATTGACCGTCAAAGAAATCATGGTATTCACTTCCGTGTTTTAGCAAAAGCTCTTCGTTTATCTGGTGGTGACCACTTACACTCTGGTACAGTTGTAGGTAAATTAGAAGGTGAACGTGAAGTAACGTTAGGTTTCGTTGACTTAATGCG 1042

C. variabilis - reference AATTGACCGTCAAAGAAATCATGGTATTCACTTCCGTGTTTTAGCAAAAGCTCTTCGTTTATCTGGTGGTGACCACTTACACTCTGGTACAGTTGTAGGTAAATTAGAAGGTGAACGTGAAGTAACGTTAGGTTTCGTTGACTTAATGCG 1049

C. variabilis - CLAW TGATGACTACATTGAGAAAGATCGTAGCCGTGGTATCTACTTCACTCAAGACTGGGTTTCTTTACCAGGTACAATGCCAGTAGCTTCTGGTGGTATTCACGTATGGCACATGCCAGCTCTAGTTGAGATTTTCGGTGATGATGCTTGTTT 1192

C. variabilis - reference TGATGACTACATTGAGAAAGATCGTAGCCGTGGTATCTACTTCACTCAAGACTGGGTTTCTTTACCAGGTACAATGCCAGTAGCTTCTGGTGGTATTCACGTATGGCACATGCCAGCTCTAGTTGAGATTTTCGGTGATGATGCTTGTTT 1199

C. variabilis - CLAW ACAATTCGGTGGTGGTACTTTAGGTCACCCTTGGGGTAACGCT-CAGGTGCTGCTGCAAACCGTGTTGCTTTAGAAGCATGTACTCAAGCGCGTAATGAAGGTCGTGACCTTGCTCGTGAAGGCGGTGATATTATCCGTGCAGCTTGCAA 1341

C. variabilis - reference ACAATTCGGTGGTGGTACTTTAGGTCACCCTTGGGGTAACGCTCCAGGTGCTGCTGCAAACCGTGTTGCTTTAGAAGCATGTACTCAAGCGCGTAATGAAGGTCGTGACCTTGCTCGTGAAGGCGGTGATATTATCCGTGCAGCTTGCAA 1349

C. variabilis - CLAW ATGGAGTCCTGAATTAGCTGCTGCTTGTGAAGTTTGGAAAGAAATTAAATTTGAATTCGAAACAATCGATACTCTTTAA 1420

C. variabilis - reference ATGGAGTCCTGAATTAGCTGCTGCTTGTGAAGTTTGGAAAGAAATTAAATTTGAATTCGAAACAATCGATACTCTTTAA 1428

O. tauri - CLAW ATGGCACCACAAACTGAAACTAAAACTGGTACTGGATTCCAGGCTGGAGTAAAAGACTACCGTTTAACATACTACACTCCTGACTACCAAGTTAAGGAAACTGACATTCTTGCTGCATTCCGTATGACTCCACAACCAGGAGTTCCTGCT 150

O. tauri - reference ATGGCACCACAAACTGAAACTAAAACTGGTACTGGATTCCAGGCTGGAGTAAAAGACTACCGTTTAACATACTACACTCCTGACTACCAAGTTAAGGAAACTGACATTCTTGCTGCATTCCGTATGACTCCACAACCAGGAGTTCCTGCT 150

O. tauri - CLAW GAAGAGTGTGGAGCAGCTGTAGCAGCTGAGTCTTCAACTGGTACTTGGACAACTGTATGGACAGATGGTCTTACTCAACTTGACCGTTACAAGGGACGTTGTTACGACCTTGAGCCGGTTCCTGGAGAAGACAACCAGTTCATCGCGTAC 300

O. tauri - reference GAAGAGTGTGGAGCAGCTGTAGCAGCTGAGTCTTCAACTGGTACTTGGACAACTGTATGGACAGATGGTCTTACTCAACTTGACCGTTACAAGGGACGTTGTTACGACCTTGAGCCGGTTCCTGGAGAAGACAACCAGTTCATCGCGTAC 300

O. tauri - CLAW GTTGCTTACCCACTCGACCTTTTCGAGGAAGGATCAGTAACAAACCTTTTCACATCAATCGTAGGAAACGTATTCGGATTCAAGGCACTTCGTGCTCTCCGTTTAGAAGACCTTCGTATTCCTGTAGCATACTGTAAGACTTTCCAGGGT 450

O. tauri - reference GTTGCTTACCCACTCGACCTTTTCGAGGAAGGATCAGTAACAAACCTTTTCACATCAATCGTAGGAAACGTATTCGGATTCAAGGCACTTCGTGCTCTCCGTTTAGAAGACCTTCGTATTCCTGTAGCATACTGTAAGACTTTCCAGGGT 450

O. tauri - CLAW GCTCCTCACGGAATTCAATGTGAGCGTGATAAGCTTAACAAGTACGGACGTGGACTTCTTGGATGTACTATTAAGCCGAAGCTTGGTCTTTCAGCTAAGAACTACGGACGTGCAGTATACGAGTGTCTTCGTGGTGGACTTGACTTCACG 600

O. tauri - reference GCTCCTCACGGAATTCAAACTGAGCGTGATAAGCTTAACAAGTACGGACGTGGACTTCTTGGATGTACTATTAAGCCTAAGCTTGGTCTTTCAGCTAAGAACTACGGACGTGCAGTATACGAGTGTCTTCGTGGTGGACTTGACTTCACG 600

O. tauri - CLAW AAGGATGACGAGAACGTAAACTCACAGCCATTCATGCGTTGGCGTGACCGTTTCCTTTTCGTAGCTGAGGCTATCTACAAGTCTCAAGCTGAGACTGGTGAGATTAAGGGACACTACCTTAACGCAACAGCTGGAAACGTTGACCAGATG 750

O. tauri - reference AAGGATGACGAGAACGTAAACTCACAGCCATTCATGCGTTGGCGTGACCGTTTCCTTTTCGTAGCTGAGGCTATCTACAAGTCTCAAGCTGAGACTGGTGAGATTAAGGGACACTACCTTAACGCAACAGCTGGAAACGTTGACCAGATG 750

O. tauri - CLAW CTTAAGCGTGCTCAAGTAGCTAAGGAACTCGGAATGCCTATTATTATGCACGACTACCTTACTGCTGGATTCACAGCTAACACAACTCTCGCTACTTACTGTCGTGAAGAAGGACTTCTCCTTCACATTCACCGTGCAATGCACGCAGTA 900

O. tauri - reference CTTAAGCGTGCTCAAGTAGCTAAGGAACTCGGAATGCCTATTATTATGCACGACTACCTTACTGCTGGATTCACAGCTAACACAACTCTCGCTACTTACTGTCGTGAAGAAGGACTTCTCCTTCACATTCACCGTGCAATGCACGCAGTA 900

O. tauri - CLAW ATTGACCGTCAGCGTAACCACGGAATCCACTTCCGTGTACTTGCTAAGGCTCTCCGTCTTTCAGGTGGTGACCACCTTCACTCAGGAACTGTAGTAGGTAAGCTTGAGGGTGAGCGTAACGTAACTCTTGGTTTCGTAGACCTTATGCGT 1050

O. tauri - reference ATTGACCGTCAGCGTAACCACGGAATCCACTTCCGTGTACTTGCTAAGGCTCTCCGTCTTTCAGGTGGTGACCACCTTCACTCAGGAACTGTAGTAGGTAAGCTTGAGGGTGAGCGTAACGTAACTCTTGGTTTCGTAGACCTTATGCGT 1050

O. tauri - CLAW GATGCTTACGTTGAGAAGGACCGTGACCGTGGAATTTACTTCTCACAAGACTGGGCTTCTCTTCCAGGTGTAATGCCAGTAGCTTCTGGTGGTATTCACGTATGGCACATGCCAGCTCTCGTAGAGATCTTCGGAGATGACGCTTGTCTT 1200

O. tauri - reference GATGCTTACGTTGAGAAGGACCGTGACCGTGGAATTTACTTCTCACAAGACTGGGCTTCTCTTCCAGGTGTAATGCCAGTAGCTTCTGGTGGTATTCACGTATGGCACATGCCAGCTCTCGTAGAGATCTTCGGAGATGACGCTTGTCTT 1200

O. tauri - CLAW CAGTTCGGTGGTGGAACTCTTGGACACCCATGGGGGAACGCTCCAGGTGCATCTGCTAACCGTGTAGCACTTGAGGCTTGTACTCAGGCTCGTAACGAAGGACGTGACCTCGCTCGTGAGGGTGGTGACGTAATCCGTGCAGCTTGTAAG 1350

O. tauri - reference CAGTTCGGTGGTGGAACTCTTGGACACCCATGGGGGAACGCTCCAGGTGCATCTGCTAACCGTGTAGCACTTGAGGCTTGTACTCAGGCTCGTAACGAAGGACGTGACCTCGCTCGTGAGGGTGGTGACGTAATCCGTGCAGCTTGTAAG 1350

O. tauri - CLAW TGGTCGCCTGAGCTCGCAGCAGCTTGTGAAGTATGGAAAGAGATCAAGTTTGAGTTCGATACGGTGGATAC 1421

O. tauri - reference TGGTCTCCTGAGCTCGCAGCAGCTTGTGAAGTATGGAAAGAGATCAAGTTTGAGTTCGATACTGTTGA--- 1418

P. provasolii - CLAW GGTGTACAAGACTACCGTCTTACTTACTACACTCCCGAGTACCAAGTAAAGGCTACTGATATTCTTGCGGCTTTCCGTATGACTCCCCAGCCTGGTGTAC-CCCCGAGGAGTGTGGTGCAGCGGTAGCAGCTGAGTCCTCCACAGGTACT 149

P. provasolii - reference GGTGTACAAGACTACCGTCTTACTTACTACACTCCCGAGTACCAAGTAAAGGCTACTGATATTCTTGCGGCTTTCCGTATGACTCCCCAGCCTGGTGTACCCCCCGAGGAGTGTGGTGCAGCGGTAGCAGCTGAGTCCTCCACAGGTACT 150

P. provasolii - CLAW TGGACTACTGTATGGACTGATGGTCTTACTTGTCTTGACAACTACAAGGGTCGTTGTTACGACCTCGAGCCCGTACCTGGTGAAGATAACCAGTACATTGCGTACATTGCTTACCCTATCGACCTCTTTGAGGAAGGTTCTGTAACTAAC 299

P. provasolii - reference TGGACTACTGTATGGACTGATGGTCTTACTTGTCTTGACAACTACAAGGGTCGTTGTTACGACCTCGAGCCCGTACCTGGTGAAGATAACCAGTACATTGCGTACATTGCTTACCCTATCGACCTCTTTGAGGAAGGTTCTGTAACTAAC 300

P. provasolii - CLAW CTCTTTACTTCTATTGTAGGTAACGTATTTGGTTTCAAGGCTCTCCGTGCTCTTCGTCTTGAGGATCTTCGTATTCCTGCTGCATACGTAAAGACATTTGCTGGTCCTCCCCACGGTATTCAGGTTGAGCGTGATAAGCTTAACAAGTAC 449

P. provasolii - reference CTCTTTACTTCTATTGTAGGTAACGTATTTGGTTTCAAGGCTCTCCGTGCTCTTCGTCTTGAGGATCTTCGTATTCCTGCTGCATACGTAAAGACATTTGCTGGTCCTCCCCACGGTATTCAGGTTGAGCGTGATAAGCTTAACAAGTAC 450

P. provasolii - CLAW GGTCGTCCTCTTCTCGGTTGTACTATTAAGCCTAAGCTCGGTCTCTCTGCTAAGAACTATGGTCGTGCAGTTTACGAGTGTCTCCGTGGTGGTCTTGACTTTACTAAGGATGATGAGAACGTAAACTCCCAGCCTTTCATGCGTTGGCGT 599

P. provasolii - reference GGTCGTCCTCTTCTCGGTTGTACTATTAAGCCTAAGCTCGGTCTCTCTGCTAAGAACTATGGTCGTGCAGTTTACGAGTGTCTCCGTGGTGGTCTTGACTTTACTAAGGATGATGAGAACGTAAACTCCCAGCCTTTCATGCGTTGGCGT 600

P. provasolii - CLAW GATCGTTTCCTCTTCTGTGCTGAGGCTATTTACAAGGCACAAGGTGAAACTGGTGAGATTAAGGGTCACTACCTTAACGCAACTGCTGGTACGGCTGAGGAAATGCTCAAGCGTGCTGAGTTCGCTGTAGACCTTGGTATGCCCATTGTT 749

P. provasolii - reference GATCGTTTCCTCTTCTGTGCTGAGGCTATTTACAAGGCACAAGGTGAAACTGGTGAGATTAAGGGTCACTACCTTAACGCAACTGCTGGTACGGCTGAGGAAATGATGAAGCGTGCTGAGTTCGCTGTAGACCTTGGTATGCCCATTGTT 750

P. provasolii - CLAW ATGCACGACTACCTTACTGGTGGTTTCACATCCAACACTACTCTTTCTAACTACTGTCGTGACAACGGTCTTCTTCTCCACATTCACCGTGCAATGCACGCGGTAATTGACCGTCAGCGTAACCACGGTATTCACTTCCGTGTTCTCGCG 899

P. provasolii - reference ATGCACGACTACCTTACTGGTGGTTTCACATCCAACACTACTCTTTCTAACTACTGTCGTGACAACGGTCTTCTTCTCCACATTCACCGTGCAATGCACGCGGTAATTGACCGTCAGCGTAACCACGGTATTCACTTCCGTGTTCTCGCG 900

P. provasolii - CLAW AAGGCTCTCCGTCTTTCTGGTGGTGACCACCTTCACTCTGGTACTGTAGTAGGTAAGCTTGAGGGTGAGCGTGAAGTAACACTTGGTTTCGTAGATCTTATGCGTGACGACTTCGTTGAGAAGGACCGTAACCGTGGTATCTACTTTACT 1049

P. provasolii - reference AAGGCTCTCCGTCTTTCTGGTGGTGACCACCTTCACTCTGGTACTGTAGTAGGTAAGCTTGAGGGTGAGCGTGAAGTAACACTTGGTTTCGTAGATCTTATGCGTGACGACTTCGTTGAGAAGGACCGTAACCGTGGTATCTACTTTACT 1050

P. provasolii - CLAW CAAGAGTGGTGTTCTATGGGTGGTGTACTCCCCGTAGCATCCGGTGGTATTCACGTATGGCACATGCCCGCACTCGTAGAGATCTTCGGTGATGACTCTGTACTCCAGTTTGGTGGTGGTACACTTGGTCACCCCTGGGGTAACGCTCCT 1199

P. provasolii - reference CAAGAGTGGTGTTCTATGGGTGGTGTAATGCCCGTAGCATCCGGTGGTATTCACGTATGGCACATGCCCGCACTCGTAGAGATCTTCGGTGATGACTCTGTACTCCAGTTTGGTGGTGGTACACTTGGTCACCCCTGGGGTAACGCTCCT 1200

P. provasolii - CLAW GGTGCGGCTGCTAACCGTGTAGCTCTTGAGGCTTGTGTACAAGCTCGTAACGAAGGTCGTGACCTCGCTCGTGAGGGTGGTGACGTAATTCGTGCGGCTGCTAAGTGGAGCCCTGAGCTTGCTGCAGCTTGTGAGGTTTGGAAGGAGATT 1349

P. provasolii - reference GGTGCGGCTGCTAACCGTGTAGCTCTTGAGGCTTGTGTACAAGCTCGTAACGAAGGTCGTGACCTCGCTCGTGAGGGTGGTGACGTAATTCGTGCGGCTGCTAAGTGGAGCCCTGAGCTTGCTGCAGCTTGTGAGGTTTGGAAGGAGATT 1350

P. provasolii - CLAW AAGTTCGAATTCGAGACTATTGATAAGCTCTAA 1382

P. provasolii - reference AAGTTCGAATTCGAGACTATTGATAAGCTCTAA 1383

A. officinalis - reference ATGTCACCACAAACAGAGACTAAAGCAAGTGTTGGATTTAAAGCTGGTGTTAAAGATTACAGATTGACTTATTATACTCCTGATTACGAAACCAAAGATACTGATATCTTGGCAGCATTCCGAGTAACTGCTCAACCCGGAGTTCCCCCT 150

A. officinalis - CLAW ATGTCACCACAAACAGAGACTAAAGCAAGTGTTGGATTTAAAGCTGGTGTTAAAGATTACAGATTGACTTATTATACTCCTGATTACGAAACCAAAGATACTGATATCTTGGCAGCATTCCGAGTAACTGCTCAACCCGGAGTT-CCCCT 149

A. officinalis - reference GAAGAAGCGGGCGCTGCGGTAGCTGCCGAATCTTCTACTGGTACATGGACAACTGTGTGGACTGATGGACTTACCAGTCTTGATCGTTACAAAGGACGATGCTACCACATCGAGCCCGTTATTGGGGAAGCAGAGCAATTTATTGCTTAT 300

A. officinalis - CLAW GAAGAAGCGGGCGCTGCGGTAGCTGCCGAATCTTCTACTGGTACATGGACAACTGTGTGGACTGATGGACTTACCAGTCTTGATCGTTACAAAGGACGATGCTACCACATCGAGCCCGTTATTGGGGAAGCAGAGCAATTTATTGCTTAT 299

A. officinalis - reference GTAGCTTATCCTTTAGACCTTTTTGAAGAAGGTTCTGTTACTAACATGTTTACTTCCATTGTGGGTAATGTATTTGGTTTCAAAGCCCTACGAGCTCTACGTTTGGAGGATCTGCGAATTCCCCCTGCTTATTCCAAAACTTTCCAAGGC 450

A. officinalis - CLAW GTAGCTTATCCTTTAGACC-TTTTGAAGAAGGTTCTGTTACTAACATGTTTACTTCCATTGTGGGTAATGTATTTGGTTTCAAAGCCCTACGAGCTCTACGTTTGGAGGATCTGCGAATT-CCCCTGCTTATTCCAAAACTTTCCAAGGC 447

A. officinalis - reference CCGCCTCATGGTATCCAAGTTGAAAGAGATAAATTGAACAAGTATGGTCGTCCCCTATTGGGATGTACTATTAAACCAAAATTGGGATTATCCGCAAAAAACTACGGTAGAGCAGTTTATGAATGTTTACGCGGTGGGCTTGATTTTACC 600

A. officinalis - CLAW CCGCCTCATGGTATCCAAGTTGAAAGAGATAAATTGAACAAGTATGGTCGTCCCCTATTGGGATGTACTATTAAACCAAAATTGGGATTATCCGCAAAAAACTACGGTAGAGCAGTTTATGAATGTTTACGCGGTGGGCTTGATTTTACC 597

A. officinalis - reference AAGGATGATGAAAACGTGAACTCACAACCTTTTATGCGTTGGCGAGACCGTTTCTGTTTTTGTGCTGAAGCTCTTTATAAAGCACAAGCGGAAACAGGTGAAATCAAAGGACATTACTTGAATGCAACTGCAGGTACATGTGAAGAAATG 750

A. officinalis - CLAW AAGGATGATGAAAACGTGAACTCACAACCTTTTATGCGTTGGCGAGACCGTTTCTGTTTTTGTGCTGAAGCTCTTTATAAAGCACAAGCGGAAACAGGTGAAATCAAAGGACATTACTTGAATGCAACTGCAGGTACATGTGAAGAAATG 747

A. officinalis - reference ATGAAAAGGGCCATATTTGCCAGAGAATTGGGAGTTCCCATCGTAATGCATGACTACTTAACTGGAGGATTTACTGCAAATACCACTTTGGCTCATTATTGCCGCGACAATGGTCTACTTCTTCACATCCACCGCGCAATGCATGCAGTT 900

A. officinalis - CLAW ATGAAAAGGGCCATATTTGCCAGAGAATTGGGAGTTCCCATCGTAATGCATGACTACTTAACTGGAGGATTTACTGCAAATACCACTTTGGCTCATTATTGCCGCGACAATGGTCTACTTCTTCACATCCACCGCGCAATGCATGCAGTT 897

A. officinalis - reference ATTGATAGACAGAAAAATCATGGTATGCATTTTCGTGTACTAGCTAAAGCATTACGTATGTCTGGTGGAGATCATATTCACGCTGGTACAGTAGTAGGTAAACTGGAAGGGGAACGTGAGATGACTTTAGGTTTTGTTGATTTATTACGT 1050

A. officinalis - CLAW ATTGATAGACAGAAAAATCATGGTATGCATTTTCGTGTACTAGCTAAAGCATTACGTATGTCTGGTGGAGATCATATTCACGCTGGTACAGTAGTAGGTAAACTGGAAGGGGAACGTGAGATGACTTTAGGTTTTGTTGATTTATTACGT 1047

A. officinalis - reference GATGATTATATTGAAAAAGACCGAAGTCGCGGTATTTTTTTCACTCAAGATTGGGTTTCTATGCCAGGTGTTATTCCCGTGGCTTCAGGGGGTATTCATGTTTGGCATATGCCTGCCCTAACCGAAATCTTTGGAGATGATTCCGTACTA 1200

A. officinalis - CLAW GATGATTATATTG-AAAAGACCGAAGTCGCGGTA-TTTTTTCACTCAAGATTGGGTTTCTATGCCAGGTGTTATTCCCGTGGCTTCA-GGGGTATTCATGTTTGGCATATGCCTGCCCTAACCGAAATCTTTGGAGATGATTCCGTACTA 1194

A. officinalis - reference CAGTTCGGTGGAGGAACTTTAGGACACCCTTGGGGAAATGCACCTGGTGCGGTAGCTAATCGGGTAGCTTTAGAAGCATGCGTACAAGCTCGTAATGAGGGACGTGATCTTGCTCGTGAGGGTAATGAGATTATCCGTGAAGCTGCCAAA 1350

A. officinalis - CLAW CAGTTCGGTGGAGGAACTTTAGGACACCCTTGGGGAAATGCACCTGGTGCGGTAGCTAATCGGGTAGCTTTAGAAGCATGCGTACAAGCTCGTAATGAGGGACGTGATCTTGCTCGTGAGGGTAATGAGATTATCCGTGAAGCTGCCAAA 1344

A. officinalis - reference TGGAGCCCGGAACTAGCCGCTGCTTGTGAAGTATGGAAAGAGATCAAATTCGAGTTCGAACCAGTAGATAAGATAGAT 1428

A. officinalis - CLAW TGGAGCCCGGAACTAGCCGCTGCTTGTGAAGTATGGAAAGAGATCAAATTCGAGTTCGAACCAGTAGATAAGATAGAT 1422

D. antarctica - CLAW ATGTCACCACAAACAGAAACTAAAGCAAGTGTTGGATTTCAAGCTGGTGTTAAAGATTATAAATTGACTTACTACACCCCGG-GTATGAAACCAAGGATACTGATATCTTGGCAGCATTCCGAGTAACTCCTCAGCCTGGGGTTC-CCCG 148

D. antarctica - reference ATGTCACCACAAACAGAAACTAAAGCAAGTGTTGGATTTCAAGCTGGTGTTAAAGATTATAAATTGACTTACTACACCCCGGAGTATGAAACCAAGGATACTGATATCTTGGCAGCATTCCGAGTAACTCCTCAGCCTGGGGTTCCCCCG 150

D. antarctica - CLAW GAAGAAGCAGGGGCTGCAGTAGCTGCCGAATCTTCTACTGGTACATGGACAACTGTTTGGACTGATGGACTTACCAGTCTTGATCGTTACAAAGGACGATGCTATCACATCGAGCCTGTTGCTGGGGAAGACAACCAATGGATCTGTTAT 298

D. antarctica - reference GAAGAAGCAGGGGCTGCAGTAGCTGCCGAATCTTCTACTGGTACATGGACAACTGTTTGGACTGATGGACTTACCAGTCTTGATCGTTACAAAGGACGATGCTATCACATCGAGCCTGTTGCTGGGGAAGACAACCAATGGATCTGTTAT 300

D. antarctica - CLAW GTAGCTTATCCATTAGACCTATTTGAAGAGGGTTCCGTTACTAACATGTTTACTTCCATTGTGGGTAACGTATTTGGTTTCAAAGCCCTACGTGCTCTACGTCTGGAGGATCTACGAATTC-CCCTGCTTATGCAAAAACTTTCCAAGGC 447

D. antarctica - reference GTAGCTTATCCATTAGACCTATTTGAAGAGGGTTCCGTTACTAACATGTTTACTTCCATTGTGGGTAACGTATTTGGTTTCAAAGCCCTACGTGCTCTACGTCTGGAGGATCTACGAATTCCCCCTGCTTATGCAAAAACTTTCCAAGGC 450

D. antarctica - CLAW CCGCCTCATGGTATCCAAGTTGAAAGAGATAAGTTGAACAAATATGGTCGTCCTTTATTGGGATGTACTATTAAACCAAAATTGGGATTATCCGC-AAAAATTACGGTAGAGCGTGTTATGAGTGTCTACGTGGTGGACTTGATTTTACC 596

D. antarctica - reference CCGCCTCATGGTATCCAAGTTGAAAGAGATAAGTTGAACAAATATGGTCGTCCTTTATTGGGATGTACTATTAAACCAAAATTGGGATTATCCGCAAAAAATTACGGTAGAGCGTGTTATGAGTGTCTACGTGGTGGACTTGATTTTACC 600

D. antarctica - CLAW AAAGATGATGAAAACGTAAACTCACAACCATTTATGCGCTGGAGAGACCGTTTTG-TTTTTGTGCCGAAGCTATTTATAAAGCACAGGCCGAAACTGGTGAAATTAAGGGGCATTACTTGAATGCGACTGCAGGTACATGTGAAGAAATG 745

D. antarctica - reference AAAGATGATGAAAACGTAAACTCACAACCATTTATGCGCTGGAGAGACCGTTTTGTTTTTTGTGCCGAAGCTATTTATAAAGCACAGGCCGAAACTGGTGAAATTAAGGGGCATTACTTGAATGCGACTGCAGGTACATGTGAAGAAATG 750

D. antarctica - CLAW ATTAAGAGAGCTGTATTTGCAAGAGAATTAGGGGTTCCTATTGTAATGCATGACTACATAACT-GGGGATTCACCGCAAATACTAGTTTGGCTCATTATTGCCGCGACAATGGCCTACTTCTTCACATTCACCGTGCAATGCATGCAGTT 894

D. antarctica - reference ATTAAGAGAGCTGTATTTGCAAGAGAATTAGGGGTTCCTATTGTAATGCATGACTACATAACTGGGGGATTCACCGCAAATACTAGTTTGGCTCATTATTGCCGCGACAATGGCCTACTTCTTCACATTCACCGTGCAATGCATGCAGTT 900

D. antarctica - CLAW ATTGATAGACAGAAAAATCATGGTATGCATTTCCGTGTATTAGCTAAAGCATTGCGTATGTCTGGG-GAGATCATATCCACGCCGGTACAGTAGTAGGTAAGTTAGAAGGGGAACGCGAAATGACTTTAGGTTTTGTTGATTTATTGCGC 1043

D. antarctica - reference ATTGATAGACAGAAAAATCATGGTATGCATTTCCGTGTATTAGCTAAAGCATTGCGTATGTCTGGGGGAGATCATATCCACGCCGGTACAGTAGTAGGTAAGTTAGAAGGGGAACGCGAAATGACTTTAGGTTTTGTTGATTTATTGCGC 1050

D. antarctica - CLAW GATGATTTTATTGA-AAAGATCGTGCTCGCGGTATCT-TTTCACTCAGGACTGGGTATCCATGCCAGGTGTTATACCGGTAGCTTCAGGTGGTATTCATGTTTGGCATATGCCAGCTCTGACCGAAATCTTTGGGGATGATTCCGTATTA 1191

D. antarctica - reference GATGATTTTATTGAAAAAGATCGTGCTCGCGGTATCTTTTTCACTCAGGACTGGGTATCCATGCCAGGTGTTATACCGGTAGCTTCAGGTGGTATTCATGTTTGGCATATGCCAGCTCTGACCGAAATCTTTGGGGATGATTCCGTATTA 1200

D. antarctica - CLAW CAATTTGGTGGAGGAACTTTAGGACATCCTTGGGGAAATGCACCTGGTGCAGCAGCTAATCGAGTGGCTTTAGAAGCCTGTGTACAAGCTCGTAACGAAGGGCGCGATCTTGCTCGTGAAGGTAATGAAATTATCCGAGCAGCTTGCAAA 1341

D. antarctica - reference CAATTTGGTGGAGGAACTTTAGGACATCCTTGGGGAAATGCACCTGGTGCAGCAGCTAATCGAGTGGCTTTAGAAGCCTGTGTACAAGCTCGTAACGAAGGGCGCGATCTTGCTCGTGAAGGTAATGAAATTATCCGAGCAGCTTGCAAA 1350

D. antarctica - CLAW TGGAGTCCTGAACTAGCCGCGGCTTGTGAAGTATGGAAAGCGATCAAATT 1391

D. antarctica - reference TGGAGTCCTGAACTAGCCGCGGCTTGTGAAGTATGGAAAGCGATCAAATT 1400

O. sativa - CLAW ATGTCACCACAAACAGAAACTAAAGCAAGTGTTGGATTTAAAGCTGGTGTTAAGGATTATAAATTGACTTACTACACCCCGGAGTACGAAACCAAGGACACTGATATCTTGGCAGCATTCCGAGTAACTCCTCAGCC-GGGGTTCCGCCC 149

O. sativa - reference ATGTCACCACAAACAGAAACTAAAGCAAGTGTTGGATTTAAAGCTGGTGTTAAGGATTATAAATTGACTTACTACACCCCGGAGTACGAAACCAAGGACACTGATATCTTGGCAGCATTCCGAGTAACTCCTCAGCCGGGGGTTCCGCCC 150

O. sativa - CLAW GAAGAAGCA-GGGCTGCAGTAGCTGCCGAATCTTCTACTGGTACATGGACAACTGTTTGGACTGATGGACTTACCAGTCTTGATCGTTACAAAGGCCGATGCTATCACATCGAGCCCGTTGTTGGGGAGGATAATCAATATATCGCTTAT 298

O. sativa - reference GAAGAAGCAGGGGCTGCAGTAGCTGCCGAATCTTCTACTGGTACATGGACAACTGTTTGGACTGATGGACTTACCAGTCTTGATCGTTACAAAGGCCGATGCTATCACATCGAGCCCGTTGTTGGGGAGGATAATCAATATATCGCTTAT 300

O. sativa - CLAW GTAGCTTATCCATTAGACCTATTTGAAGAGGGTTCTGTTACTAACATGTTTACTTCCATTGTGGGTAACGTATTTGGTTTCAAAGCCCTACGCGCTCTACGTCTGGAGGATCTGCGAATTC-CCCTACTTATTCAAAAACTTTCCAAGGT 447

O. sativa - reference GTAGCTTATCCATTAGACCTATTTGAAGAGGGTTCTGTTACTAACATGTTTACTTCCATTGTGGGTAACGTATTTGGTTTCAAAGCCCTACGCGCTCTACGTCTGGAGGATCTGCGAATTCCCCCTACTTATTCAAAAACTTTCCAAGGT 450

O. sativa - CLAW CCGCCTCATGGTATCCAAGTTGAAAGGGATAAGTTGAACAAATACGGTCGTCCTTTATTGGGATGTACTATTAAACCAAAATTGGGATTATCTGCAAAAAATTATGGTAGAGCATGTTATGAGTGTCTACGCGGTGGACTTGATTTTACC 597

O. sativa - reference CCGCCTCATGGTATCCAAGTTGAAAGGGATAAGTTGAACAAATACGGTCGTCCTTTATTGGGATGTACTATTAAACCAAAATTGGGATTATCTGCAAAAAATTATGGTAGAGCATGTTATGAGTGTCTACGCGGTGGACTTGATTTTACC 600

O. sativa - CLAW AAAGATGATGAAAACGTAAACTCACAACCATTTATGCGTTGGAGGGACCGTTTTGTCT-TTGTGCCGAAGCTATTTATAAATCACAGGCCGAAACCGGTGAAATTAAG-GGCATTACTTGAATGCGACTGCAGGTACATGCGAAGAAATG 745

O. sativa - reference AAAGATGATGAAAACGTAAACTCACAACCATTTATGCGTTGGAGGGACCGTTTTGTCTTTTGTGCCGAAGCTATTTATAAATCACAGGCCGAAACCGGTGAAATTAAGGGGCATTACTTGAATGCGACTGCAGGTACATGCGAAGAAATG 750

O. sativa - CLAW ATTAAAAGAGCTGTATTTGCGAGGGAATTA-GGGTTCCTATTGTAATGCATGACTACTTAACC-GGGGATTCACCGCAAATACTAGTTTGGCTCATTATTGCCGCGACAACGGCCTACTTCTTCACATTCACCGAGCAATGCATGCAGTT 893

O. sativa - reference ATTAAAAGAGCTGTATTTGCGAGGGAATTAGGGGTTCCTATTGTAATGCATGACTACTTAACCGGGGGATTCACCGCAAATACTAGTTTGGCTCATTATTGCCGCGACAACGGCCTACTTCTTCACATTCACCGAGCAATGCATGCAGTT 900

O. sativa - CLAW ATTGATAGACAGAAAAATCATGGTATGCATTTCCGTGTATTAGCTAAAGCATTGCGTATGTCTGGG-GAGATCATATCCACGCTGGTACAGTAGTAGGTAAGTTAGAAGGGGAACGCGAAATGACTTTAGGTTTTGTTGATTTATTGCGC 1042

O. sativa - reference ATTGATAGACAGAAAAATCATGGTATGCATTTCCGTGTATTAGCTAAAGCATTGCGTATGTCTGGGGGAGATCATATCCACGCTGGTACAGTAGTAGGTAAGTTAGAAGGGGAACGCGAAATGACTTTAGGTTTTGTTGATTTATTGCGC 1050

O. sativa - CLAW GATGATTTTATTGAAAAAGATCGTGCTCGCGGTATC-TTTTCACTCAGGACTGGGTATCCATGCCAGGTGTTATACCGGTGGCTTCA-GGGGTATTCATGTTTGGCATATGCCAGCTCTGACCGAAATCTTTGGAGATGATTCTGTATTG 1190

O. sativa - reference GATGATTTTATTGAAAAAGATCGTGCTCGCGGTATCTTTTTCACTCAGGACTGGGTATCCATGCCAGGTGTTATACCGGTGGCTTCAGGGGGTATTCATGTTTGGCATATGCCAGCTCTGACCGAAATCTTTGGAGATGATTCTGTATTG 1200

O. sativa - CLAW CAATTTGGTGGAGGAACTTTAGGACATCCTTGGGGTAATGCACCTGGTGCAGCAGCTAATCGGGTGGCTTTAGAAGCCTGTGTACAAGCTCGTAACGAAGGGCGCGATCTTGCTCGTGAAGGTAATGAAATTATCCGATCAGCTTGCAAA 1340

O. sativa - reference CAATTTGGTGGAGGAACTTTAGGACATCCTTGGGGTAATGCACCTGGTGCAGCAGCTAATCGGGTGGCTTTAGAAGCCTGTGTACAAGCTCGTAACGAAGGGCGCGATCTTGCTCGTGAAGGTAATGAAATTATCCGATCAGCTTGCAAA 1350

O. sativa - CLAW TGGAGTCCTGAACTAGCCGCAGCTTGTGAAATATGGAAAGCGATCAAATT 1390

O. sativa - reference TGGAGTCCTGAACTAGCCGCAGCTTGTGAAATATGGAAAGCGATCAAATT 1400

S. polyrhiza - CLAW ATGTCACCACAAACAGAGACTAAAGCAAGTGCTGGATTCAAAGCTGGTGTTAAAGATTACAAATTGACTTATTATACTCCTGAGTATGAGACAAAAGATACGGATATCTTGGCAGCATTCCGAGTAACTCCTCAACCTGGAGTTCCACCT 150

S. polyrhiza - reference ATGTCACCACAAACAGAGACTAAAGCAAGTGCTGGATTCAAAGCTGGTGTTAAAGATTACAAATTGACTTATTATACTCCTGAGTATGAGACAAAAGATACGGATATCTTGGCAGCATTCCGAGTAACTCCTCAACCTGGAGTTCCACCT 150

S. polyrhiza - CLAW GAAGAAGCAGGGGCTGCAGTAGCTGCCGAATCTTCTACTGGTACATGGACAACTGTGTGGACTGATGGACTTACCAGCCTTGATCGTTACAAAGGACGATGCTACCATATCGAACCCGTTGTTGGAGAGGAAAATCAATATATTGCTTAT 300

S. polyrhiza - reference GAAGAAGCAGGGGCTGCAGTAGCTGCCGAATCTTCTACTGGTACATGGACAACTGTGTGGACTGATGGACTTACCAGCCTTGATCGTTACAAAGGACGATGCTACCATATCGAACCCGTTGTTGGAGAGGAAAATCAATATATTGCTTAT 300

S. polyrhiza - CLAW GTAGCTTACCCTTTAGACCTTTTTGAAGAAGGTTCTGTTACTAACATGTTTACTTCCATTGTAGGTAATGTATTTGGGTTTAAAGCTTTACGAGCTCTACGTCTGGAAGATTTGCGAATTCCTCCTGCTTATTCCAAAACTTTCCAAGGC 450

S. polyrhiza - reference GTAGCTTACCCTTTAGACCTTTTTGAAGAAGGTTCTGTTACTAACATGTTTACTTCCATTGTAGGTAATGTATTTGGGTTTAAAGCTTTACGAGCTCTACGTCTGGAAGATTTGCGAATTCCTCCTGCTTATTCCAAAACTTTCCAAGGC 450

S. polyrhiza - CLAW CCACCTCATGGGATCCAAGTTGAGAGAGATAAATTGAACAAGTATGGTCGTCCTCTATTGGGATGTACCATCAAACCAAAATTGGGATTATCCGCGAAA-ACTACGGTAGAGCGGTTTATGAATGTCTACGTGGTGGACTTGATTTTACC 599

S. polyrhiza - reference CCACCTCATGGGATCCAAGTTGAGAGAGATAAATTGAACAAGTATGGTCGTCCTCTATTGGGATGTACCATCAAACCAAAATTGGGATTATCCGCGAAAAACTACGGTAGAGCGGTTTATGAATGTCTACGTGGTGGACTTGATTTTACC 600

S. polyrhiza - CLAW AAGGATGATGAAAACGTGAACTCACAACCATTTATGCGTTGGAGAGACCGTTTCTTATTTTGTGCTGAAGCAATTTATAAAGCACAAGCTGAAACAGGTGAAATTAAAGGGCATTACTTAAATGCTACTGCAGGTACTTGTGAAGAAATG 749

S. polyrhiza - reference AAGGATGATGAAAACGTGAACTCACAACCATTTATGCGTTGGAGAGACCGTTTCTTATTTTGTGCTGAAGCAATTTATAAAGCACAAGCTGAAACAGGTGAAATTAAAGGGCATTACTTAAATGCTACTGCAGGTACTTGTGAAGAAATG 750

S. polyrhiza - CLAW ATCAAAAGGGCTGTGTTTGCCAGAGAATTGGGAGTCCCTATTGTAATGCATGACTACTTGACA-GGGGATTCACTGCAAATACTAGTTTAGCACATTATTGCCGAGACAACGGCCTACTTCTTCACATCCACCGTGCAATGCATGCAGTT 898

S. polyrhiza - reference ATCAAAAGGGCTGTGTTTGCCAGAGAATTGGGAGTCCCTATTGTAATGCATGACTACTTGACAGGGGGATTCACTGCAAATACTAGTTTAGCACATTATTGCCGAGACAACGGCCTACTTCTTCACATCCACCGTGCAATGCATGCAGTT 900

S. polyrhiza - CLAW ATTGATAGACAGAAAAATCATGGTATGCATTTCCGTGTACTAGCTAAAGCATTACGTATGTCTGGTGGGGATCATATTCACGCTGGTACAGTAGTAGGTAAACTGGAAGGTGAACGTGAGATGACTTTAGGTTTTGTTGATTTATTACGT 1048

S. polyrhiza - reference ATTGATAGACAGAAAAATCATGGTATGCATTTCCGTGTACTAGCTAAAGCATTACGTATGTCTGGTGGGGATCATATTCACGCTGGTACAGTAGTAGGTAAACTGGAAGGTGAACGTGAGATGACTTTAGGTTTTGTTGATTTATTACGT 1050

S. polyrhiza - CLAW GATGATTATATTGAAAAAGACCGAAGTCGTGGTATTTTCTTCACTCAAGATTGGGTCTCTATGCCAGGTGTTATACCTGTGGCTTCAGGGGGTATTCATGTTTGGCATATGCCTGCCCTGACCGAGATCTTTGGAGATGATTCCGTACTA 1198

S. polyrhiza - reference GATGATTATATTGAAAAAGACCGAAGTCGTGGTATTTTCTTCACTCAAGATTGGGTCTCTATGCCAGGTGTTATACCTGTGGCTTCAGGGGGTATTCATGTTTGGCATATGCCTGCCCTGACCGAGATCTTTGGAGATGATTCCGTACTA 1200

S. polyrhiza - CLAW CAGTTTGGTGGCGGAACTTTAGGACACCCTTGGGGAAATGCACCTGGTGCAGTAGCTAACCGTGTAGCTTTAGAAGCGTGTGTACAAGCTCGTAATGAGGGACGTGATCTTGCTCGTGAAGGTAATGAAATTATCCGTGAAGCTTGCAAA 1348

S. polyrhiza - reference CAGTTTGGTGGCGGAACTTTAGGACACCCTTGGGGAAATGCACCTGGTGCAGTAGCTAACCGTGTAGCTTTAGAAGCGTGTGTACAAGCTCGTAATGAGGGACGTGATCTTGCTCGTGAAGGTAATGAAATTATCCGTGAAGCTTGCAAA 1350

S. polyrhiza - CLAW TGGAGTCCTGAACTAGCCGCTGCTTGTGAAGTTTGGAAAGCGATCAAATTTGAGTTCGAACCAGTAGATAAGCTAGATG 1427

S. polyrhiza - reference TGGAGTCCTGAACTAGCCGCTGCTTGTGAAGTTTGGAAAGCGATCAAATTTGAGTTCGAACCAGTAGATAAGCTAGATG 1429

A. sinensis - CLAW ATGTCACCACAAACAGAGACTAAAGCAAGTGTTGGATTCAAAGCTGGTGTTAAAGAGTATAAATTGACTTATTATACTCCTGAATATGAAACCAAAGATACTGATATCTTGGCAGCATTCCGAGTAACTCCTCAACCTGGAGTTCCGCCT 150

A. sinensis - reference ATGTCACCACAAACAGAGACTAAAGCAAGTGTTGGATTCAAAGCTGGTGTTAAAGAGTATAAATTGACTTATTATACTCCTGAATATGAAACCAAAGATACTGATATCTTGGCAGCATTCCGAGTAACTCCTCAACCTGGAGTTCCGCCT 150

A. sinensis - CLAW GAGGAAGCAGGGGCTGCGGTAGCTGCTGAATCTTCTACTGGTACATGGACAACTGTGTGGACCGACGGGCTTACCAGCCTTGATCGTTACAAAGGGCGATGCTACCACATCGAGCCCGTTGCTGGGGAAGAAAATCAATATATATGTTAT 300

A. sinensis - reference GAGGAAGCAGGGGCTGCGGTAGCTGCTGAATCTTCTACTGGTACATGGACAACTGTGTGGACCGACGGGCTTACCAGCCTTGATCGTTACAAAGGGCGATGCTACCACATCGAGCCCGTTGCTGGGGAAGAAAATCAATATATATGTTAT 300

A. sinensis - CLAW GTAGCTTACCCCTTAGACC-TTTTGAAGAGGGTTCTGTTACTAACATGTTTACTTCCATTGTTGGTAATGTATTTGGGTTCAAAGCCCTGCGCGCTCTACGTCTAGAAGATCTGCGAATCCCTACTTCTTATATTAAAACTTTCCAAGGT 449

A. sinensis - reference GTAGCTTACCCCTTAGACCTTTTTGAAGAGGGTTCTGTTACTAACATGTTTACTTCCATTGTTGGTAATGTATTTGGGTTCAAAGCCCTGCGCGCTCTACGTCTAGAAGATCTGCGAATCCCTACTTCTTATATTAAAACTTTCCAAGGT 450

A. sinensis - CLAW CCGCCTCATGGCATCCAAGTTGAAAGAGATAAATTGAACAAGTACGGCCGTCCCCTATTGGGATGTACTATTAAACCTAAATTGGGGTTATCCGCTAAAAACTACGGTAGAGCGGTTTATGAATGTCTACGTGGTGGACTTGATTTTACC 599

A. sinensis - reference CCGCCTCATGGCATCCAAGTTGAAAGAGATAAATTGAACAAGTACGGCCGTCCCCTATTGGGATGTACTATTAAACCTAAATTGGGGTTATCCGCTAAAAACTACGGTAGAGCGGTTTATGAATGTCTACGTGGTGGACTTGATTTTACC 600

A. sinensis - CLAW AAAGATGATGAGAATGTGAACTCCCAACCATTTATGCGTTGGAGAGACCGTTTCTTATTTTGTGCCGAAGCAATTTATAAAGCACAGGCTGAAACAGGTGAAATCAAAGGGCATTACTTGAATGCTACTGCGGGTACATGCGAAGAAATG 749

A. sinensis - reference AAAGATGATGAGAATGTGAACTCCCAACCATTTATGCGTTGGAGAGACCGTTTCTTATTTTGTGCCGAAGCAATTTATAAAGCACAGGCTGAAACAGGTGAAATCAAAGGGCATTACTTGAATGCTACTGCGGGTACATGCGAAGAAATG 750

A. sinensis - CLAW ATCAAAAGGGCTGTATTTGCCAGAGAATTAGGAGCTCCTATCGTAATGCATGACTATTTAAC--GGGGATTCACGGCAAATACTAGCTTGGCTCATTATTGCCGAGATAATGGTCTCCTTCTTCACATCCATCGCGCAATGCACGCAGTT 897

A. sinensis - reference ATCAAAAGGGCTGTATTTGCCAGAGAATTAGGAGCTCCTATCGTAATGCATGACTATTTAACGGGGGGATTCACGGCAAATACTAGCTTGGCTCATTATTGCCGAGATAATGGTCTCCTTCTTCACATCCATCGCGCAATGCACGCAGTT 900

A. sinensis - CLAW ATTGATAGACAGAAGAATCACGGTATGCACTTCCGTGTACTAGCTAAAGCCTTACGTATGTCTGGTGGAGATCATATTCACGCTGGTACAGTAGTAGGTAAACTTGAAGGAGAAAGAGACATAACTTTGGGTTTTGTTGATTTACTACGT 1047

A. sinensis - reference ATTGATAGACAGAAGAATCACGGTATGCACTTCCGTGTACTAGCTAAAGCCTTACGTATGTCTGGTGGAGATCATATTCACGCTGGTACAGTAGTAGGTAAACTTGAAGGAGAAAGAGACATAACTTTGGGTTTTGTTGATTTACTACGT 1050

A. sinensis - CLAW GATGATTTTATTG-AAAAGATAGAAGCCGTGGTATTTATTTCACTCAAGATTGGGTCTCTCTACCAGGTGTTATACCGGTAGCTTC-GGGGGTATTCACGTTTGGCATATGCCTGCTTTGACCGAGATCTTTGGTGATGATGCCGTACTA 1195

A. sinensis - reference GATGATTTTATTGAAAAAGATAGAAGCCGTGGTATTTATTTCACTCAAGATTGGGTCTCTCTACCAGGTGTTATACCGGTAGCTTCGGGGGGTATTCACGTTTGGCATATGCCTGCTTTGACCGAGATCTTTGGTGATGATGCCGTACTA 1200

A. sinensis - CLAW CAATTTGGTGGAGGAACTTTAGGACACCCTTGGGGAAATGCACCGGGTGCCGTCGCTAATCGAGTAGCTCTAGAAGCATGTGTACAAGCTCGTAATGAGGGACGTGATCTTGCTCGCGAGGGTAATGAAATTATCCGTACGGCTAGCAAA 1345

A. sinensis - reference CAATTTGGTGGAGGAACTTTAGGACACCCTTGGGGAAATGCACCGGGTGCCGTCGCTAATCGAGTAGCTCTAGAAGCATGTGTACAAGCTCGTAATGAGGGACGTGATCTTGCTCGCGAGGGTAATGAAATTATCCGTACGGCTAGCAAA 1350

A. sinensis - CLAW TGGAGTCCTGAACTAGCTGCTGCTTGTGAAGTATGGAAAGAGATCAAATTTGAATTCCAAGCAGTGGATA 1415

A. sinensis - reference TGGAGTCCTGAACTAGCTGCTGCTTGTGAAGTATGGAAAGAGATCAAATTTGAATTCCAAGCAGTGGATA 1420

C. sativa - reference ATGTCACCACAAACAGAGACTAAAGCAAGTGTTGGATTCAAAGCTGGTGTTAAAGATTATAAATTGACTTATTACACTCCGGAATATCAAACCAAAGATACTGATATCTTGGCAGCATTTCGAGTAACTCCTCAACCTGGAGTTCCCCCT 150

C. sativa - CLAW ATGTCACCACAAACAGAGACTAAAGCAAGTGTTGGATTCAAAGCTGGTGTTAAAGATTATAAATTGACTTATTACACTCCGGAATATCAAACCAAAGATACTGATATCTTGGCAGCATTTCGAGTAACTCCTCAACCTGGAGTT-CCCCT 149

C. sativa - reference GAAGAAGCAGGGGCTGCGGTAGCTGCTGAATCTTCTACTGGTACATGGACAACTGTATGGACTGATGGGCTTACCAGCCTTGATCGCTACAAAGGTCGATGCTACCACATCGAGCCCGTTGCTGGAGAAGAAAATCAATTTATTGCTTAT 300

C. sativa - CLAW GAAGAAGCAGGGGCTGCGGTAGCTGCTGAATCTTCTACTGGTACATGGACAACTGTATGGACTGATGGGCTTACCAGCCTTGATCGCTACAAAGGTCGATGCTACCACATCGAGCCCGTTGCTGGAGAAGAAAATCAATTTATTGCTTAT 299

C. sativa - reference GTAGCTTATCCCTTAGACCTTTTTGAAGAAGGTTCTGTTACTAACATGTTTACTTCCATTGTGGGTAATGTATTTGGGTTCAAGGCCCTGCGCGCTCTACGTCTGGAAGATTTGAGAATCCCTACTTCTTATACTAAAACTTTCCAAGGT 450

C. sativa - CLAW GTAGCTTATCCCTTAGACC-TTTTGAAGAAGGTTCTGTTACTAACATGTTTACTTCCATTGTGGGTAATGTATTTGGGTTCAAGGCCCTGCGCGCTCTACGTCTGGAAGATTTGAGAATCCCTACTTCTTATACTAAAACTTTCCAAGGT 448

C. sativa - reference CCGCCTCATGGGATCCAAGTTGAGAGAGATAAATTGAACAAGTATGGTCGCCCACTATTGGGATGTACTATTAAACCTAAATTGGGGTTATCCGCTAAGAATTACGGTAGAGCAGTTTATGAATGTCTTCGCGGTGGACTTGATTTTACC 600

C. sativa - CLAW CCGCCTCATGGGATCCAAGTTGAGAGAGATAAATTGAACAAGTATGGTCGCCCACTATTGGGATGTACTATTAAACCTAAATTGGGGTTATCCGCTAAGAATTACGGTAGAGCAGTTTATGAATGTCTTCGCGGTGGACTTGATTTTACC 598

C. sativa - reference AAAGATGATGAGAACGTAAATTCCCAACCATTTATGCGTTGGAGAGACCGTTTCTTATTTTGTGCAGAAGCAATTTATAAATCACAGTCTGAAACAGGGGAAATCAAAGGACATTACTTGAATGCTACTGCAGGTACATGTGAAGAAATG 750

C. sativa - CLAW AAAGATGATGAGAACGTAAATTCCCAACCATTTATGCGTTGGAGAGACCGTTTCTTATTTTGTGCAGAAGCAATTTATAAATCACAGTCTGAAACA-GGGAAATCAAAGGACATTACTTGAATGCTACTGCAGGTACATGTGAAGAAATG 747

C. sativa - reference ATGAAAAGGGCTGTATTTGCCAGAGAATTGGGAGTTCCTATCGTAATGCATGATTACTTAACAGGAGGATTCACTGCAAATACTAGTCTGGCTCATTATTGTCGAGATAATGGTCTACTTCTTCACATCCACCGTGCAATGCATGCGGTT 900

C. sativa - CLAW ATGAAAAGGGCTGTATTTGCCAGAGAATTGGGAGTTCCTATCGTAATGCATGATTACTTAACAGGAGGATTCACTGCAAATACTAGTCTGGCTCATTATTGTCGAGATAATGGTCTACTTCTTCACATCCACCGTGCAATGCATGCGGTT 897

C. sativa - reference ATTGATAGACAAAAGAATCATGGTATACACTTCCGTGTACTAGCTAAAGCGTTACGTATGTCTGGTGGAGATCATATCCATTCAGGTACTGTAGTAGGTAAACTTGAAGGGGAAAGAGAAATCACTTTAGGCTTTGTTGATTTACTACGT 1050

C. sativa - CLAW ATTGATAGAC-AAAGAATCATGGTATACACTTCCGTGTACTAGCTAAAGCGTTACGTATGTCTGGTGGAGATCATATCCATTCAGGTACTGTAGTAGGTAAACTTGAA-GGGAAAGAGAAATCACTTTAGGCTTTGTTGATTTACTACGT 1045

C. sativa - reference GATGATTTTATTGAAAAAGATCGAAGCCGTGGTATTTATTTCACTCAAGATTGGGTCTCTCTACCAGGTGTTCTGCCTGTGGCTTCAGGGGGTATTCACGTTTGGCATATGCCTGCTTTGACCGAGATCTTTGGAGATGATTCCGTACTA 1200

C. sativa - CLAW GATGATTTTATTG-AAAAGATCGAAGCCGTGGTATTTATTTCACTCAAGATTGGGTCTCTCTACCAGGTGTTCTGCCTGTGGCTTCA-GGGGTATTCACGTTTGGCATATGCCTGCTTTGACCGAGATCTTTGGAGATGATTCCGTACTA 1193

C. sativa - reference CAATTTGGTGGAGGAACTTTAGGACATCCTTGGGGAAATGCACCCGGTGCTGTCGCTAATCGAGTAGCTCTAGAAGCATGTGTACAAGCTCGTAATGAGGGACGTGATCTTGCTCGTGAGGGTAATGAAATTATTCGTGAGGCTTGTAAA 1350

C. sativa - CLAW CAATTTGGTGGAGGAACTTTAGGACATCCTT-GGGAAATGCACCCGGTGCTGTCGCTAATCGAGTAGCTCTAGAAGCATGTGTACAAGCTCGTAATGAGGGACGTGATCTTGCTCGTGAGGGTAATGAAATTATTCGTGAGGCTTGTAAA 1342

C. sativa - reference TGGAGTCCTGAACTAGCTGCTGCTTGTGAAGTTTGGAAGGAAATCAAATTT 1401

C. sativa - CLAW TGGAGTCCTGAACTAGCTGCTGCTTGTGAAGTTTGGAAGGAAATCAAATTT 1393

C. avellana - CLAW ATGTCACCACAAACAGAGACTAAAGCAAGTGTTGGATTCAAAGCTGGTGTTAAAGATTATAAATTAACTTATTATACTCCTGACTATGAAACCAAAGATACTGATATCTTGGCAGCGTTCCGAGTAACTCCTCAACCTGGAGTTCCGCCT 150

C. avellana - reference ATGTCACCACAAACAGAGACTAAAGCAAGTGTTGGATTCAAAGCTGGTGTTAAAGATTATAAATTAACTTATTATACTCCTGACTATGAAACCAAAGATACTGATATCTTGGCAGCGTTCCGAGTAACTCCTCAACCTGGAGTTCCGCCT 150

C. avellana - CLAW GAGGAAGCAGGGGCAGCAGTAGCTGCTGAATCTTCTACTGGTACATGGACAACTGTGTGGACCGATGGACTTACTAGTCTTGATCGTTACAAAGGACGATGCTACCACATCGAGCCAGTTGCTGGAGAAGAAAGTCAATTTATTGCTTAT 300

C. avellana - reference GAGGAAGCAGGGGCAGCAGTAGCTGCTGAATCTTCTACTGGTACATGGACAACTGTGTGGACCGATGGACTTACTAGTCTTGATCGTTACAAAGGACGATGCTACCACATCGAGCCAGTTGCTGGAGAAGAAAGTCAATTTATTGCTTAT 300

C. avellana - CLAW GTAGCTTACCCCTTAGACC-TTTTGAAGAAGGTTCTGTTACTAACATGTTTACTTCCATTGTGGGTAATGTATTTGGATTCAAGGCCCTGCGTGCTCTACGTCTGGAGGATTTGCGAATCCCTCCTGCTTATTCTAAAACTTTCCAAGGC 449

C. avellana - reference GTAGCTTACCCCTTAGACCTTTTTGAAGAAGGTTCTGTTACTAACATGTTTACTTCCATTGTGGGTAATGTATTTGGATTCAAGGCCCTGCGTGCTCTACGTCTGGAGGATTTGCGAATCCCTCCTGCTTATTCTAAAACTTTCCAAGGC 450

C. avellana - CLAW CCGCCTCACGGCATCCAAGTTGAGAGAGATAAATTAAACAAGTATGGCCGCC-CCTATTGGGATGTACTATTAAACCTAAATTGGGATTATCCGCTAAGAATTACGGTAGAGCGGTTTATGAATGTCTCCGCGGTGGGCTTGATTTTACC 598

C. avellana - reference CCGCCTCACGGCATCCAAGTTGAGAGAGATAAATTAAACAAGTATGGCCGCCCCCTATTGGGATGTACTATTAAACCTAAATTGGGATTATCCGCTAAGAATTACGGTAGAGCGGTTTATGAATGTCTCCGCGGTGGGCTTGATTTTACC 600

C. avellana - CLAW AAAGATGATGAAAACGTGAATTCCCAACCATTTATGCGTTGGAGAGACCGTTTCCTATTTTGTGCCGAAGCAATTTATAAAGCGCAGGCTGAAACAGGTGAAATCAAAGGGCATTACTTGAATGCTACTGCAGGTACATGCGAAGAAATG 748

C. avellana - reference AAAGATGATGAAAACGTGAATTCCCAACCATTTATGCGTTGGAGAGACCGTTTCCTATTTTGTGCCGAAGCAATTTATAAAGCGCAGGCTGAAACAGGTGAAATCAAAGGGCATTACTTGAATGCTACTGCAGGTACATGCGAAGAAATG 750

C. avellana - CLAW ATCAAAAGGGCTGTATTTGCCAGAGAATTGGGAGTTCCTATCGTAATGCATGACTACTTAACC-GGGGATTCACTGCAAATACTAGCTTGGCTCATTATTGCCGGGATAATGGTCTACTTCTTCACATCCATCGTGCAATGCATGCAGTT 897

C. avellana - reference ATCAAAAGGGCTGTATTTGCCAGAGAATTGGGAGTTCCTATCGTAATGCATGACTACTTAACCGGGGGATTCACTGCAAATACTAGCTTGGCTCATTATTGCCGGGATAATGGTCTACTTCTTCACATCCATCGTGCAATGCATGCAGTT 900

C. avellana - CLAW ATTGATAGACAGAAGAATCATGGTATACACTTTCGTGTACTAGCTAAAGCGTTACGCATGTCTGGTGGAGATCATATTCACGCTGGTACCGTAGTAGGTAAACTTGAAGGGGAAAGAGAGATCACTTTAGGCTTTGTTGATTTACTGCGT 1047

C. avellana - reference ATTGATAGACAGAAGAATCATGGTATACACTTTCGTGTACTAGCTAAAGCGTTACGCATGTCTGGTGGAGATCATATTCACGCTGGTACCGTAGTAGGTAAACTTGAAGGGGAAAGAGAGATCACTTTAGGCTTTGTTGATTTACTGCGT 1050

C. avellana - CLAW GATGATTATATTGA-AAAGATCGAAGCCGCGGTATTTATTTTACTCAAGATTGGGTCTCTCTACCAGGTGTTCTGCCCGTGGCTTCAGGGGGTATTCACGTTTGGCATATGCCTGCTCTGACCGAAATCTTTGGAGATGATTCCGTACTA 1196

C. avellana - reference GATGATTATATTGAAAAAGATCGAAGCCGCGGTATTTATTTTACTCAAGATTGGGTCTCTCTACCAGGTGTTCTGCCCGTGGCTTCAGGGGGTATTCACGTTTGGCATATGCCTGCTCTGACCGAAATCTTTGGAGATGATTCCGTACTA 1200

C. avellana - CLAW CAATTCGGCGGAGGAACTTTAGGGCACCCTTGGGGAAATGCACCGGGTGCTGTAGCTAATCGAGTAGCTCTAGAAGCATGTGTACAAGCTCGTAATGAGGGACGTGATCTTGCTCGTGAGGGTAATGAAATTATTCGTGCGGCCGGTAAA 1346

C. avellana - reference CAATTCGGCGGAGGAACTTTAGGGCACCCTTGGGGAAATGCACCGGGTGCTGTAGCTAATCGAGTAGCTCTAGAAGCATGTGTACAAGCTCGTAATGAGGGACGTGATCTTGCTCGTGAGGGTAATGAAATTATTCGTGCGGCCGGTAAA 1350

C. avellana - CLAW TGGAGTCCTGAGCTAGCTGCCGCTTGTGAAGTATGGAAGGAGATCAAATTTGAATTCCCAGCAATGGATA 1416

C. avellana - reference TGGAGTCCTGAGCTAGCTGCCGCTTGTGAAGTATGGAAGGAGATCAAATTTGAATTCCCAGCAATGGATA 1420

E. polybractea - CLAW ATGTCACCACAAACAGAGACTAAAGCAAGTGTTGGATTCAAAGCTGGTGTTAAAGATTATAAACTGACTTATTATACTCCTGACTATGAAACCAAAGATACTGATATCTTGGCAGCATTCCGAGTAACTCCTCAACCTGGAGTTCCTGCT 150

E. polybractea - reference ATGTCACCACAAACAGAGACTAAAGCAAGTGTTGGATTCAAAGCTGGTGTTAAAGATTATAAACTGACTTATTATACTCCTGACTATGAAACCAAAGATACTGATATCTTGGCAGCATTCCGAGTAACTCCTCAACCTGGAGTTCCTGCT 150

E. polybractea - CLAW GAGGAAGCAGGGGCTGCGGTAGCTGCTGAATCTTCTACTGGTACATGGACAACTGTGTGGACCGATGGGCTTACCAGCCTTGATCGTTATAAAGGAAGATGCTACCACATCGAGCCTGTTGCTGGAGAAGAAAATCAATATATATGTTAT 300

E. polybractea - reference GAGGAAGCAGGGGCTGCGGTAGCTGCTGAATCTTCTACTGGTACATGGACAACTGTGTGGACCGATGGGCTTACCAGCCTTGATCGTTATAAAGGAAGATGCTACCACATCGAGCCTGTTGCTGGAGAAGAAAATCAATATATATGTTAT 300

E. polybractea - CLAW GTAGCTTACCCTTTAGACCTTT-TGAAGAAGGTTCTGTTACTAATATGTTTACTTCCATTGTGGGTAATGTATTTGGGTTCAAAGCCCTGCGCGCTCTACGTCTGGAGGATCTGCGAATCCCTACTTCCTATACGAAAACTTTCCAAGGC 449

E. polybractea - reference GTAGCTTACCCTTTAGACCTTTTTGAAGAAGGTTCTGTTACTAATATGTTTACTTCCATTGTGGGTAATGTATTTGGGTTCAAAGCCCTGCGCGCTCTACGTCTGGAGGATCTGCGAATCCCTACTTCCTATACGAAAACTTTCCAAGGC 450

E. polybractea - CLAW CCGCCTCATGGCATCCAAGTTGAGAGAGATAAATTGAACAAATATGGGCGTCCCCTATTGGGATGTACTATTAAACCGAAATTGGGGTTATCCGCTAAGAACTACGGTAGAGCAGTTTATGAATGTCTTCGTGGTGGACTTGATTTTACG 599

E. polybractea - reference CCGCCTCATGGCATCCAAGTTGAGAGAGATAAATTGAACAAATATGGGCGTCCCCTATTGGGATGTACTATTAAACCGAAATTGGGGTTATCCGCTAAGAACTACGGTAGAGCAGTTTATGAATGTCTTCGTGGTGGACTTGATTTTACG 600

E. polybractea - CLAW AAAGATGATGAGAACGTGAACTCACAACCATTTATGCGTTGGAGAGACCGTTTCTTATTTTGTGCCGAAGCAATTTTTAAATCACAGGCTGAAACAGGTGAAATCAAAGGGCATTACTTGAATGCTACTGCAGGTACATGCGAAGAAATG 749

E. polybractea - reference AAAGATGATGAGAACGTGAACTCACAACCATTTATGCGTTGGAGAGACCGTTTCTTATTTTGTGCCGAAGCAATTTTTAAATCACAGGCTGAAACAGGTGAAATCAAAGGGCATTACTTGAATGCTACTGCAGGTACATGCGAAGAAATG 750

E. polybractea - CLAW ATGAAAAGGGCTGTATTTGCCAGAGAATTGGGAGTTCCTATCGTAATGCATGACTACTTAACAGGG-GATTCACTGCAAATACTAGCTTGGCTCATTATTGCCGAGATAATGGTCTACTTCTTCACATCCATCGTGCAATGCATGCAGTT 898

E. polybractea - reference ATGAAAAGGGCTGTATTTGCCAGAGAATTGGGAGTTCCTATCGTAATGCATGACTACTTAACAGGGGGATTCACTGCAAATACTAGCTTGGCTCATTATTGCCGAGATAATGGTCTACTTCTTCACATCCATCGTGCAATGCATGCAGTT 900

E. polybractea - CLAW ATTGATAGACAGAAAAATCATGGTATGCACTTTAGGGTACTAGCTAAAGCCTTACGTATGTCTGGTGGAGATCATATTCACGCTGGTACTGTAGTAGGTAAACTTGAAGGAGAAAGAGACATTACTTTGGGCTTTGTTGATTTACTACGT 1048

E. polybractea - reference ATTGATAGACAGAAAAATCATGGTATACACTTTAGGGTACTAGCTAAAGCCTTACGTATGTCTGGTGGAGATCATATTCACGCTGGTACTGTAGTAGGTAAACTTGAAGGAGAAAGAGACATTACTTTGGGCTTTGTTGATTTACTACGT 1050

E. polybractea - CLAW GATGATTTTATTGAAAAAGATCGAAGCCGCGGTATTTATTTCACTCAAGATTGGGTCTCTCTACCAGGTGTTCTGCCCGTAGCTTCTG-GGGTATTCACGTTTGGCATATGCCTGCTCTGACCGAGATCTTTGGAGATGATTCCGTACTA 1197

E. polybractea - reference GATGATTTTATTGAAAAAGATCGAAGCCGCGGTATTTATTTCACTCAAGATTGGGTCTCTCTACCAGGTGTTCTGCCCGTAGCTTCTGGGGGTATTCACGTTTGGCATATGCCTGCTCTGACCGAGATCTTTGGAGATGATTCCGTACTA 1200

E. polybractea - CLAW CAATTCGGCGGAGGAACTTTAGGACACCCTTGGGGAAATGCACCGGGTGCCGTAGCTAATCGAGTAGCTCTAGAAGCATGCGTACAAGCTCGTAATGAGGGACGTGATCTTGCTCGTGAGGGTAATGAAATTATCCGTGAGGCTAGCAAA 1347

E. polybractea - reference CAATTCGGCGGAGGAACTTTAGGACACCCTTGGGGAAATGCACCGGGTGCCGTAGCTAATCGAGTAGCTCTAGAAGCATGCGTACAAGCTCGTAATGAGGGACGTGATCTTGCTCGTGAGGGTAATGAAATTATCCGTGAGGCTAGCAAA 1350

E. polybractea - CLAW TGGAGTCCTGAACTAGCTGCTGCTTGTGAAGTATGGAAAGAGATCAAATTTGAATTC 1404

E. polybractea - reference TGGAGTCCTGAACTAGCTGCTGCTTGTGAAGTATGGAAAGAGATCAAATTT------ 1401

G. longicalyx - CLAW ATGTCACCACAAACAGAGACTAAAGCAAGTGTTGGATTCAAAGCTGGTGTTAAAGAGTATAAATTGACTTATTATACTCCTGAATATGAAGTCAAAGATACTGATATCTTGGCAGCCTTCCGAGTAACTCCTCAACCCGGAGTTCCGCCT 150

G. longicalyx - reference ATGTCACCACAAACAGAGACTAAAGCAAGTGTTGGATTCAAAGCTGGTGTTAAAGAGTATAAATTGACTTATTATACTCCTGAATATGAAGTCAAAGATACTGATATCTTGGCAGCCTTCCGAGTAACTCCTCAACCCGGAGTTCCGCCT 150

G. longicalyx - CLAW GAGGAAGCAGGGGCCGCGGTAGCTGCTGAATCTTCTACTGGTACATGGACAACCGTGTGGACCGATGGGCTTACCAGCCTTGATCGTTACAAAGGGCGATGCTACGACATTGAGCCCGTTCCTGGAGAAGAAGATCAATATATATGTTAT 300

G. longicalyx - reference GAGGAAGCAGGGGCCGCGGTAGCTGCTGAATCTTCTACTGGTACATGGACAACCGTGTGGACCGATGGGCTTACCAGCCTTGATCGTTACAAAGGGCGATGCTACGACATTGAGCCCGTTCCTGGAGAAGAAGATCAATATATATGTTAT 300

G. longicalyx - CLAW GTAGCTTACCCTTTAGACCT-TTTGAAGAAGGTTCTGTTACTAACATGTTTACTTCCATTGTGGGTAATGTATTTGGGTTCAAAGCCCTGCGCGCTCTACGTCTAGAGGATCTGCGAATCCCTACTGCTTATATTAAAACTTTCCAAGGC 449

G. longicalyx - reference GTAGCTTACCCTTTAGACCTTTTTGAAGAAGGTTCTGTTACTAACATGTTTACTTCCATTGTGGGTAATGTATTTGGGTTCAAAGCCCTGCGCGCTCTACGTCTAGAGGATCTGCGAATCCCTACTGCTTATATTAAAACTTTCCAAGGC 450

G. longicalyx - CLAW CCGCCTCATGGCATACAGGTTGAAAGAGATAAATTGAACAAGTATGGTCGCCC-CTATTAGGATGTACTATTAAACCTAAATT-GGGTTATCCGCTAAGAACTACGGTAGAGCAGTTTATGAATGTCTACGTGGCGGGCTTGATTTTACC 597

G. longicalyx - reference CCGCCTCATGGCATACAGGTTGAAAGAGATAAATTGAACAAGTATGGTCGCCCCCTATTAGGATGTACTATTAAACCTAAATTGGGGTTATCCGCTAAGAACTACGGTAGAGCAGTTTATGAATGTCTACGTGGCGGGCTTGATTTTACC 600

G. longicalyx - CLAW AAAGATGATGAGAATGTGAACTCCCAACCATTTATGCGCTGGAGAGACCGTTTCTTATTTTGTGCCGAAGCAATTTATAAATCACAGGCTGAAACAGGTGAAATCAAAGGGCATTACTTGAATGCTACTGCAGGTACATGTGAAGAAATG 747

G. longicalyx - reference AAAGATGATGAGAATGTGAACTCCCAACCATTTATGCGCTGGAGAGACCGTTTCTTATTTTGTGCCGAAGCAATTTATAAATCACAGGCTGAAACAGGTGAAATCAAAGGGCATTACTTGAATGCTACTGCAGGTACATGTGAAGAAATG 750

G. longicalyx - CLAW ATCAAAAGGGCCGTGTGTGCTAGAGAATTGGGAGTTCCTATCGTAATGCACGACTACTTAACAGGTGGGTTCACTGCAAATACTAGCTTGGCTCATTATTGCCGAGATAATGGTCTACTTCTTCACATCCATCGCGCAATGCACGCAGTT 897

G. longicalyx - reference ATCAAAAGGGCCGTGTGTGCTAGAGAATTGGGAGTTCCTATCGTAATGCACGACTACTTAACAGGTGGGTTCACTGCAAATACTAGCTTGGCTCATTATTGCCGAGATAATGGTCTACTTCTTCACATCCATCGCGCAATGCACGCAGTT 900

G. longicalyx - CLAW ATTGATAGACAGAAGAATCATGGTATGCACTTTCGTGTACTAGCTAAAGCTTTACGTATGTCTGGTGGAGATCATATTCACGCTGGTACAGTAGTAGGTAAACTTGAAGGAGAAAGGGACATAACTTTGGGCTTTGTTGATTTACTACGT 1047

G. longicalyx - reference ATTGATAGACAGAAGAATCATGGTATGCACTTTCGTGTACTAGCTAAAGCTTTACGTATGTCTGGTGGAGATCATATTCACGCTGGTACAGTAGTAGGTAAACTTGAAGGAGAAAGGGACATAACTTTGGGCTTTGTTGATTTACTACGT 1050

G. longicalyx - CLAW GATGATTTTATTGA-AAAGATCGAAGCCGTGGTATTTATTTCACTCAAGATTGGGTTTCTATGCCAGGTGTTCTGCCCGTAGCTTCGGG--GTATTCACGTTTGGCATATGCCTGCTTTGACCGAGATCTTTGGAGATGATTCCGTACTA 1194

G. longicalyx - reference GATGATTTTATTGAAAAAGATCGAAGCCGTGGTATTTATTTCACTCAAGATTGGGTTTCTATGCCAGGTGTTCTGCCCGTAGCTTCGGGGGGTATTCACGTTTGGCATATGCCTGCTTTGACCGAGATCTTTGGAGATGATTCCGTACTA 1200

G. longicalyx - CLAW CAATTCGGTGGAGGAACTTTAGGACACCCTT-GGGAAATGCACCGGGTGCCGTAGCTAATCGAGTAGCTTTAGAAGCATGTGTACAAGCTCGTAATGAGGGACGTGACCTTGCCCGCGAGGGTAATGAAATTATCCGCGAGGCTAGCAAA 1343

G. longicalyx - reference CAATTCGGTGGAGGAACTTTAGGACACCCTTGGGGAAATGCACCGGGTGCCGTAGCTAATCGAGTAGCTTTAGAAGCATGTGTACAAGCTCGTAATGAGGGACGTGACCTTGCCCGCGAGGGTAATGAAATTATCCGCGAGGCTAGCAAA 1350

G. longicalyx - CLAW TGGAGTCCTGAACTAGCTGCTGCTTGTGAAGTATGGAAGGCGATCAAATTTGAATTCGACGCAGTGGATAAATTAGAT 1421

G. longicalyx - reference TGGAGTCCTGAACTAGCTGCTGCTTGTGAAGTATGGAAGGCGATCAAATTTGAATTCGACGCAGTGGATAAATTAGAT 1428

L. sativus - CLAW ATGTCACCACAAACAGAAACTAAAGCAAAGGTTGGGTTCAAAGCTGGTGTTAAAGATTATAAATTGACTTATTATACTCCTGACTATCAAACCAAAGATACTGATATCTTGGCAGCATTCCGAGTAACTCCTCAACCTGGAGTTCCGCCT 150

L. sativus - reference ATGTCACCACAAACAGAAACTAAAGCAAAGGTTGGGTTCAAAGCTGGTGTTAAAGATTATAAATTGACTTATTATACTCCTGACTATCAAACCAAAGATACTGATATCTTGGCAGCATTCCGAGTAACTCCTCAACCTGGAGTTCCGCCT 150

L. sativus - CLAW GAAGAAGCAGGTGCAGCGGTAGCTGCAGAATCTTCCACTGGTACATGGACAACTGTGTGGACCGATGGACTTACCAGCCTTGATCGTTATAAAGGACGCTGCTACGAGATCGAGCCTGTTCCTGGAGAAGATAATCAATTTATTGCTTAT 300

L. sativus - reference GAAGAAGCAGGTGCAGCGGTAGCTGCAGAATCTTCCACTGGTACATGGACAACTGTGTGGACCGATGGACTTACCAGCCTTGATCGTTATAAAGGACGCTGCTACGAGATCGAGCCTGTTCCTGGAGAAGATAATCAATTTATTGCTTAT 300

L. sativus - CLAW GTAGCTTATCCCTTAGACC-TTTTGAAGAAGGTTCTGTTACTAACATGTTTACCTCCATTGTAGGTAATGTATTTGGGTTCAAGGCCTTGCGCGCTCTACGTCTGGAAGATTTGCGAATCCCTAATGCTTATGTTAAAACTTTCCAAGGT 449

L. sativus - reference GTAGCTTATCCCTTAGACCTTTTTGAAGAAGGTTCTGTTACTAACATGTTTACCTCCATTGTAGGTAATGTATTTGGGTTCAAGGCCTTGCGCGCTCTACGTCTGGAAGATTTGCGAATCCCTAATGCTTATGTTAAAACTTTCCAAGGT 450

L. sativus - CLAW CCTCCTCACGGAATCCAAGTTGAGAGAGATAAATTGAACAAGTATGGACGTCCCCTATTGGGATGTACTATAAAACCCAAATTGGGTTTATCAGCTAAGAATTATGGTAGAGCAGTTTATGAATGTCTCCGCGGGGGACTTGATTTTACC 599

L. sativus - reference CCTCCTCACGGAATCCAAGTTGAGAGAGATAAATTGAACAAGTATGGACGTCCCCTATTGGGATGTACTATAAAACCCAAATTGGGTTTATCAGCTAAGAATTATGGTAGAGCAGTTTATGAATGTCTCCGCGGGGGACTTGATTTTACC 600

L. sativus - CLAW AAAGATGATGAAAATGTGAACTCCCAACCATTTATGCGTTGGAGAGACCGTTTCTTATTTTGTGCCGAAGCAATTTATAAATCACAGGCCGAAACA-GGGAAATCAAAGGACATTATTTGAATGCTACTGCGGGTACATGTGAAGAAATG 748

L. sativus - reference AAAGATGATGAAAATGTGAACTCCCAACCATTTATGCGTTGGAGAGACCGTTTCTTATTTTGTGCCGAAGCAATTTATAAATCACAGGCCGAAACAGGGGAAATCAAAGGACATTATTTGAATGCTACTGCGGGTACATGTGAAGAAATG 750

L. sativus - CLAW CTA-AAAGAGCTGTATTTGCTAGAGAATTGGGCGTTCCTATCGTAATGCATGACTACTTAACAGGTGGATTCACTGCAAATACTACCCTGTCTCACTATTGCCGCGATAATGGTCTACTTCTTCATATCCACCGTGCAATGCATGCAGTT 897

L. sativus - reference CTAAAAAGAGCTGTATTTGCTAGAGAATTGGGCGTTCCTATCGTAATGCATGACTACTTAACAGGTGGATTCACTGCAAATACTACCCTGTCTCACTATTGCCGCGATAATGGTCTACTTCTTCATATCCACCGTGCAATGCATGCAGTT 900

L. sativus - CLAW ATCGATAGAC-AAAAAATCATGGTATGCACTTTCGTGTATTAGCTAAAGCCTTACGTTTGTCTGGTGGAGATCATATTCACGCTGGTACTGTAGTAGGTAAACTTGAAGGAGAAAGGGAGATTACTTTAGGTTTTGTTGATTTACTACGT 1046

L. sativus - reference ATCGATAGACAAAAAAATCATGGTATGCACTTTCGTGTATTAGCTAAAGCCTTACGTTTGTCTGGTGGAGATCATATTCACGCTGGTACTGTAGTAGGTAAACTTGAAGGAGAAAGGGAGATTACTTTAGGTTTTGTTGATTTACTACGT 1050

L. sativus - CLAW GATGATTATATTG-AAAAGATCGAAGTCGCGGTATTTATTTCACTCAGGATTGGGTTTCTTTACCAGGTGTTATCCCTGTTGCTTCA-GGGGTATTCACGTTTGGCATATGCCTGCTCTGACCGAGATATTTGGAGATGATTCTGTACTC 1194

L. sativus - reference GATGATTATATTGAAAAAGATCGAAGTCGCGGTATTTATTTCACTCAGGATTGGGTTTCTTTACCAGGTGTTATCCCTGTTGCTTCAGGGGGTATTCACGTTTGGCATATGCCTGCTCTGACCGAGATATTTGGAGATGATTCTGTACTC 1200

L. sativus - CLAW CAATTCGGTGGAGGAACTTTAGGACACCCTTGGGGAAATGCACCTGGTGCCGTAGCGAATCGAGTAGCTCTGGAAGCATGTGTACAAGCTCGGAATGAGGGACGTGATCTTGCTCGCGAGGGTAATGCAATTATCCGTCAAGCTTGCAAA 1344

L. sativus - reference CAATTCGGTGGAGGAACTTTAGGACACCCTTGGGGAAATGCACCTGGTGCCGTAGCGAATCGAGTAGCTCTGGAAGCATGTGTACAAGCTCGGAATGAGGGACGTGATCTTGCTCGCGAGGGTAATGCAATTATCCGTCAAGCTTGCAAA 1350

L. sativus - CLAW TGGAGTCCTGAATTAGCTGCTGCTTGTGAAGTCTGGAAGGAAATCAAATTTGAATTCCCAGCAATGGATACT 1416

L. sativus - reference TGGAGTCCTGAATTAGCTGCTGCTTGTGAAGTCTGGAAGGAAATCAAATTTGAATTCCCAGCAATGGATA-- 1420

M. truncatula - CLAW ------CCACAAACAGAAACTAAAGCAACGGTTGGGTTCAAAGCTGGTGTTAAAGATTATCGATTGACTTATTATACTCCTGACTATGAAACCAAAGATACTGATATCTTGGCAGCATTCCGAGTAAGTCCTCAACCTGGAGTTCCGGCT 144

M. truncatula - reference ATGTCACCACAAACAGAAACTAAAGCAACGGTTGGGTTCAAAGCTGGTGTTAAAGATTATCGATTGACTTATTATACTCCTGACTATGAAACCAAAGATACTGATATCTTGGCAGCATTCCGAGTAAGTCCTCAACCTGGAGTTCCGGCT 150

M. truncatula - CLAW GAAGAAGCAGGTGCAGCGGTAGCTGCCGAATCTTCCACTGGGACATGGACAACCGTGTGGACCGATGGACTTACCAGTCTTGATCGTTATAAAGGACGCTGCTACCACATCGAACCTGTTGCTGGAGAAGAGAGTCAATTTATTGCTTAT 294

M. truncatula - reference GAAGAAGCAGGTGCAGCGGTAGCTGCCGAATCTTCCACTGGGACATGGACAACCGTGTGGACCGATGGACTTACCAGTCTTGATCGTTATAAAGGACGCTGCTACCACATCGAACCTGTTGCTGGAGAAGAGAGTCAATTTATTGCTTAT 300

M. truncatula - CLAW GTAGCTTATCCCTTAGACCT-TTTGAAGAAGGTTCTGTTACTAACATGTTTACCTCCATTGTAGGTAATGTATTTGGGTTCAAGGCCTTGCGTGCTCTACGTCTGGAAGATTTGCGAATCCCCGTTGCTTATGTTAAAACTTTCCAAGGT 443

M. truncatula - reference GTAGCTTATCCCTTAGACCTTTTTGAAGAAGGTTCTGTTACTAACATGTTTACCTCCATTGTAGGTAATGTATTTGGGTTCAAGGCCTTGCGTGCTCTACGTCTGGAAGATTTGCGAATCCCCGTTGCTTATGTTAAAACTTTCCAAGGT 450

M. truncatula - CLAW CCTCCTCACGGAATCCAAGTTGAGAGAGATAAATTGAACAAATATGGACGTCCCCTATTGGGATGTACTATTAAACCTAAATTGGGTTTATCCGCTAAAAATTACGGTAGAGCAGTTTATGAATGTCTACGTGGTGGACTTGATTTTACC 593

M. truncatula - reference CCTCCTCACGGAATCCAAGTTGAGAGAGATAAATTGAACAAATATGGACGTCCCCTATTGGGATGTACTATTAAACCTAAATTGGGTTTATCCGCTAAAAATTACGGTAGAGCAGTTTATGAATGTCTACGTGGTGGACTTGATTTTACC 600

M. truncatula - CLAW AAAGATGATGAAAATGTGAACTCCCAACCATTTATGCGTTGGAGAGACCGTTTCTTATTTTGTGCCGAAGCTATTTATAAAGCACAGGCCGAAACTGGTGAAATCAAAGGACATTATTTGAATGCTACTGCGGGCACCTGTGAAGACATG 743

M. truncatula - reference AAAGATGATGAAAATGTGAACTCCCAACCATTTATGCGTTGGAGAGACCGTTTCTTATTTTGTGCCGAAGCTATTTATAAAGCACAGGCCGAAACTGGTGAAATCAAAGGACATTATTTGAATGCTACTGCGGGCACCTGTGAAGACATG 750

M. truncatula - CLAW ATGAAAAGAGCTGTATTTGCTAGAGAATTGGGCGTGCCTATCGTAATGCATGACTACTTAACCGGTGGATTCACTGCAAATACTACCTTGGCTCACTATTGCCGCGATAATGGTCTACTTCTTCATATCCACCGTGCAATGCATGCAGTT 893

M. truncatula - reference ATGAAAAGAGCTGTATTTGCTAGAGAATTGGGCGTGCCTATCGTAATGCATGACTACTTAACCGGTGGATTCACTGCAAATACTACCTTGGCTCACTATTGCCGCGATAATGGTCTACTTCTTCATATCCACCGTGCAATGCATGCAGTT 900

M. truncatula - CLAW ATTGATAGACAGAAAAATCATGGTATGCACTTTCGTGTATTAGCTAAAGCGTTACGTATGTCAGGTGGAGATCATATTCACGCTGGTACTGTAGTAGGTAAACTGGAAGGAGAAAGGGATATTACTTTAGGTTTTGTTGATTTACTACGT 1043

M. truncatula - reference ATTGATAGACAGAAAAATCATGGTATGCACTTTCGTGTATTAGCTAAAGCGTTACGTATGTCAGGTGGAGATCATATTCACGCTGGTACTGTAGTAGGTAAACTGGAAGGAGAAAGGGATATTACTTTAGGTTTTGTTGATTTACTACGT 1050

M. truncatula - CLAW GATGATTTTGTTGAAAAAGATAGAAGTCGCGGTATTT-TTTCACTCAGGATTGGGTTTCTTTACCTGGTGTTCTGCCTGTTGCTTCAGGTGGTATTCATGTTTGGCATATGCCTGCTCTGACCGAGATATTTGGAGATGATTCTGTACTT 1192

M. truncatula - reference GATGATTTTGTTGAAAAAGATAGAAGTCGCGGTATTTTTTTCACTCAGGATTGGGTTTCTTTACCTGGTGTTCTGCCTGTTGCTTCAGGTGGTATTCATGTTTGGCATATGCCTGCTCTGACCGAGATATTTGGAGATGATTCTGTACTT 1200

M. truncatula - CLAW CAATTCGGTGGAGGAACTTTAGGACACCCTTGGGGAAATGCACCTGGTGCCGTAGCGAATCGAGTAGCTCTGGAAGCATGTGTACAAGCTCGTAATGAAGGACGTGATCTTGCTCGTGAGGGTAATGAAATTATCCGTGAAGCTACCAAA 1342

M. truncatula - reference CAATTCGGTGGAGGAACTTTAGGACACCCTTGGGGAAATGCACCTGGTGCCGTAGCGAATCGAGTAGCTCTGGAAGCATGTGTACAAGCTCGTAATGAAGGACGTGATCTTGCTCGTGAGGGTAATGAAATTATCCGTGAAGCTACCAAA 1350

M. truncatula - CLAW TGGAGTCCTGAATTAGCTGCTGCTTGTGAAGTCTGGAAGGAGATCAAATTTGAATTCCCAGCAATGGATACTATTTAA 1420

M. truncatula - reference TGGAGTCCTGAATTAGCTGCTGCTTGTGAAGTCTGGAAGGAGATCAAATTTGAATTCCCAGCAATGGATA-------- 1420

P. ginseng - CLAW ATGTCACCACAAACAGAGACTAAAGCAAGTGTTGGATTCAAAGCTGGTGTTAAAGATTACAAATTGACTTATTATACTCCTGACTATGATCCCAAAGATACTGATATCTTGGCAGCATTCCGAGTAACTCCTCAACCTGGAGTTCCAGCT 150

P. ginseng - reference ATGTCACCACAAACAGAGACTAAAGCAAGTGTTGGATTCAAAGCTGGTGTTAAAGATTACAAATTGACTTATTATACTCCTGACTATGATCCCAAAGATACTGATATCTTGGCAGCATTCCGAGTAACTCCTCAACCTGGAGTTCCAGCT 150

P. ginseng - CLAW GAAGAAGCAGGGGCCGCGGTAGCTGCCGAATCTTCTACTGGTACATGGACAACTGTGTGGACCGATGGACTTACTAGCCTTGATCGTTACAAAGGGCGATGCTACAAAATAGAGCCCGTTGCTGGAGAAGAAACTCAATTTATTGCTTAT 300

P. ginseng - reference GAAGAAGCAGGGGCCGCGGTAGCTGCCGAATCTTCTACTGGTACATGGACAACTGTGTGGACCGATGGACTTACTAGCCTTGATCGTTACAAAGGGCGATGCTACAAAATAGAGCCCGTTGCTGGAGAAGAAACTCAATTTATTGCTTAT 300

P. ginseng - CLAW GTAGCTTACCCATTAGACCTTTTTGAAGAAGGTTCTGTTACTAACATGTTTACTTCCATTGTAGGTAATGTATTTGGGTTCAAAGCCCTGCGTGCTCTACGTCTGGAAGATCTGCGAATCCCTGTTGCTTATGTTAAAACTTTCCAAGGC 450

P. ginseng - reference GTAGCTTACCCATTAGACCTTTTTGAAGAAGGTTCTGTTACTAACATGTTTACTTCCATTGTAGGTAATGTATTTGGGTTCAAAGCCCTGCGTGCTCTACGTCTGGAAGATCTGCGAATCCCTGTTGCTTATGTTAAAACTTTCCAAGGC 450

P. ginseng - CLAW CCGCCTCATGGCATCCAAGTTGAGAGAGATAAATTGAACAAGTATGGTCGTCCCCTGTTGGGATGTACTATTAAACCTAAATTGGGGTTATCTGCTAAAAACTACGGTAGAGCGGTTTATGAATGTCTCCGGGGTGGACTTGATTTTACC 600

P. ginseng - reference CCGCCTCATGGCATCCAAGTTGAGAGAGATAAATTGAACAAGTATGGTCGTCCCCTGTTGGGATGTACTATTAAACCTAAATTGGGGTTATCTGCTAAAAACTACGGTAGAGCGGTTTATGAATGTCTCCGGGGTGGACTTGATTTTACC 600

P. ginseng - CLAW AAAGACGATGAGAACGTGAACTCACAACCATTTATGCGTTGGAGAGATCGTTTCTTATTTTGTGCCGAAGCACTTTATAAAGCACAGGCTGAAACAGGTGAAATCAAAGGGCATTACTTGAATGCTACTGCGGGTACATGCGAAGACATG 750

P. ginseng - reference AAAGACGATGAGAACGTGAACTCACAACCATTTATGCGTTGGAGAGATCGTTTCTTATTTTGTGCCGAAGCACTTTATAAAGCACAGGCTGAAACAGGTGAAATCAAAGGGCATTACTTGAATGCTACTGCGGGTACATGCGAAGACATG 750

P. ginseng - CLAW ATGAAAAGGGCTGTATTTGCCAGAGAATTGGGAGTTCCTATCGTAATGCATGATTACATAACA-GGGGATTCACCGCAAATACTACCTTGGCTCATTATTGCCGAGATAATGGCCTACTTCTTCACATCCACCGCGCAATGCATGCAGTT 899

P. ginseng - reference ATGAAAAGGGCTGTATTTGCCAGAGAATTGGGAGTTCCTATCGTAATGCATGATTACATAACAGGGGGATTCACCGCAAATACTACCTTGGCTCATTATTGCCGAGATAATGGCCTACTTCTTCACATCCACCGCGCAATGCATGCAGTT 900

P. ginseng - CLAW ATTGATAGACAGAAGAATCATGGTATGCACTTTCGTGTACTAGCTAAAGCGTTACGTATGTCTGGTGGAGATCATATTCACTCCGGTACCGTAGTAGGTAAACTTGAAGGGGAAAGAGACATCACTTTGGGCTTTGTTGATTTACTGCGT 1049

P. ginseng - reference ATTGATAGACAGAAGAATCATGGTATGCACTTTCGTGTACTAGCTAAAGCGTTACGTATGTCTGGTGGAGATCATATTCACTCCGGTACCGTAGTAGGTAAACTTGAAGGGGAAAGAGACATCACTTTGGGCTTTGTTGATTTACTGCGT 1050

P. ginseng - CLAW GATGATTTCATTGAAAAAGATCGAAGTCGCGGTATTTATTTCACCCAAGATTGGGTCTCTCTACCAGGTGTTCTGCCCGTGGCTTC-GGGGGTATTCACGTTTGGCATATGCCTGCTCTGACCGAGATCTTTGGGGATGATTCCGTACTA 1198

P. ginseng - reference GATGATTTCATTGAAAAAGATCGAAGTCGCGGTATTTATTTCACCCAAGATTGGGTCTCTCTACCAGGTGTTCTGCCCGTGGCTTCGGGGGGTATTCACGTTTGGCATATGCCTGCTCTGACCGAGATCTTTGGGGATGATTCCGTACTA 1200

P. ginseng - CLAW CAGTTCGGTGGAGGAACTTTAGGACACCCTTGGGGAAATGCACCCGGTGCCGTAGCTAATCGAGTAGCTCTAGAAGCATGTGTACAAGCTCGTAATGAGGGACGTGATCTTGCTCGTGAAGGTAATGAAATTATCCGCGAGGCTGCTAAA 1348

P. ginseng - reference CAGTTCGGTGGAGGAACTTTAGGACACCCTTGGGGAAATGCACCCGGTGCCGTAGCTAATCGAGTAGCTCTAGAAGCATGTGTACAAGCTCGTAATGAGGGACGTGATCTTGCTCGTGAAGGTAATGAAATTATCCGCGAGGCTGCTAAA 1350

P. ginseng - CLAW TGGAGCCCTGAACTAGCTGCTGCTTGTGAGGTATGGAAGGAGATCAAATTT 1399

P. ginseng - reference TGGAGCCCTGAACTAGCTGCTGCTTGTGAGGTATGGAAGGAGATCAAATTT 1401

P. dulcis - CLAW ATGTCACCACAAACAGAGACTAAAGCAAGTGTTGGATTCAAAGCTGGTGTTAAAGATTATAAATTGACTTATTATACTCCTGACTATGAAACCAAAGATACTGATATCTTGGCAGCATTTCGAGTAACTCCTCAACCTGGAGTTCCACCT 150

P. dulcis - reference ATGTCACCACAAACAGAGACTAAAGCAAGTGTTGGATTCAAAGCTGGTGTTAAAGATTATAAATTGACTTATTATACTCCTGACTATGAAACCAAAGATACTGATATCTTGGCAGCATTTCGAGTAACTCCTCAACCTGGAGTTCCACCT 150

P. dulcis - CLAW GAAGAAGCA-GGGCAGCGGTAGCTGCTGAATCTTCTACTGGTACATGGACAACTGTATGGACTGACGGGCTTACTAGTCTTGATCGTTACAAAGGTCGATGCTACCACATCGAGCCCGTTGCTGGAGAAGAAAGTCAATTTATTGCTTAT 299

P. dulcis - reference GAAGAAGCAGGGGCAGCGGTAGCTGCTGAATCTTCTACTGGTACATGGACAACTGTATGGACTGACGGGCTTACTAGTCTTGATCGTTACAAAGGTCGATGCTACCACATCGAGCCCGTTGCTGGAGAAGAAAGTCAATTTATTGCTTAT 300

P. dulcis - CLAW GTAGCTTACCCCTTAGACC-TTTTGAAGAGGGTTCTGTTACTAACATGTTTACTTCCATTGTAGGTAATGTGTTTGGGTTCAAGGCCCTGCGCGCTCTACGTCTGGAGGATTTGCGAATCCCTCCTGCTTATGTTAAAACTTTCCAAGGC 448

P. dulcis - reference GTAGCTTACCCCTTAGACCTTTTTGAAGAGGGTTCTGTTACTAACATGTTTACTTCCATTGTAGGTAATGTGTTTGGGTTCAAGGCCCTGCGCGCTCTACGTCTGGAGGATTTGCGAATCCCTCCTGCTTATGTTAAAACTTTCCAAGGC 450

P. dulcis - CLAW CCGCCTCATGGGATCCAAGTTGAGAGAGATAAATTGAACAAGTATGGCCGC-CCCTATTGGGATGTACTATTAAACCTAAATTGGGGTTATCCGCTAAGAATTACGGTAGAGCAGTTTATGAATGTCTCCGCGGTGGACTTGATTTTACC 597

P. dulcis - reference CCGCCTCATGGGATCCAAGTTGAGAGAGATAAATTGAACAAGTATGGCCGCCCCCTATTGGGATGTACTATTAAACCTAAATTGGGGTTATCCGCTAAGAATTACGGTAGAGCAGTTTATGAATGTCTCCGCGGTGGACTTGATTTTACC 600

P. dulcis - CLAW AAAGATGATGAGAATGTTAATTCCCAACCATTTATGCGTTGGAGAGACCGTTTCTTATTTTGTGCCGAAGCAATTTATAAAGCACAGGCTGAAACAGGTGAAATCAAAGGGCATTACTTGAACGCTACTGCAGGTACATGCGAAGAGATG 747

P. dulcis - reference AAAGATGATGAGAATGTTAATTCCCAACCATTTATGCGTTGGAGAGACCGTTTCTTATTTTGTGCCGAAGCAATTTATAAAGCACAGGCTGAAACAGGTGAAATCAAAGGGCATTACTTGAACGCTACTGCAGGTACATGCGAAGAGATG 750

P. dulcis - CLAW ATCAAAAGAGCTGTATTTGCCAGAGAATT-GGGGTTCCTATCGTAATGCATGATTACTTAACA-GGGGATTCACTGCAAATACTAGCTTGGCTCATTATTGCCGAGATAATGGTTTACTTCTTCACATCCACCGTGCAATGCATGCAGTT 895

P. dulcis - reference ATCAAAAGAGCTGTATTTGCCAGAGAATTGGGGGTTCCTATCGTAATGCATGATTACTTAACAGGGGGATTCACTGCAAATACTAGCTTGGCTCATTATTGCCGAGATAATGGTTTACTTCTTCACATCCACCGTGCAATGCATGCAGTT 900

P. dulcis - CLAW ATTGATAGACAGAAGAATCATGGTATGCACTTTCGTGTACTAGCTAAAGCGTTACGTATGTCTGGTGGAGATCATATACACGCTGGTACCGTAGTAGGTAAACTTGAGGGG--AAAGGAGATCACTTTAGGCTTTGTTGATTTACTACGT 1043

P. dulcis - reference ATTGATAGACAGAAGAATCATGGTATGCACTTTCGTGTACTAGCTAAAGCGTTACGTATGTCTGGTGGAGATCATATACACGCTGGTACCGTAGTAGGTAAACTTGAGGGGGAAAGGGAGATCACTTTAGGCTTTGTTGATTTACTACGT 1050

P. dulcis - CLAW GATGATTTTGTTG-AAAAGATCGAAGCCGCGGTATTTATTTCACTCAAGATTGGGTCTCTATGCCAGGTGTTTTGCCTGTAGCTTCAG-GGGTATTCACGTTTGGCATATGCCTGCTCTGACCGAGATCTTTGGAGATGATTCTGTACTA 1191

P. dulcis - reference GATGATTTTGTTGAAAAAGATCGAAGCCGCGGTATTTATTTCACTCAAGATTGGGTCTCTATGCCAGGTGTTTTGCCTGTAGCTTCAGGGGGTATTCACGTTTGGCATATGCCTGCTCTGACCGAGATCTTTGGAGATGATTCTGTACTA 1200

P. dulcis - CLAW CAATTTGGCGGCGGAACTTTAGGGCACCCTT-GGGAAATGCACCTGGTGCCGTAGCTAATCGAGTAGCTCTAGAAGCATGTGTACAAGCTCGTAATGAGGGACGTGATCTTGCTCGTGAGGGTAATGAAATTATTCGCGAGGCTAGTAAA 1340

P. dulcis - reference CAATTTGGCGGCGGAACTTTAGGGCACCCTTGGGGAAATGCACCTGGTGCCGTAGCTAATCGAGTAGCTCTAGAAGCATGTGTACAAGCTCGTAATGAGGGACGTGATCTTGCTCGTGAGGGTAATGAAATTATTCGCGAGGCTAGTAAA 1350

P. dulcis - CLAW TGGAGTCCTGAACTAGCTGCTGCTTGTGAAATATGGAAGGAGATCAAATTTGAATTCCAAGCAATGGATA 1410

P. dulcis - reference TGGAGTCCTGAACTAGCTGCTGCTTGTGAAATATGGAAGGAGATCAAATTTGAATTCC------------ 1408

S. commersonii - CLAW ATGTCACCACAAACAGAGACTAAAGCAAGTGTTGGATTCAAAGCTGGTGTTAAAGAGTACAAATTGACTTATTATACTCCTGAGTACCAAACCAAGGATACTGATATATTGGCAGCATTCCGAGTAACTCCTCAACCTGGAGTTCCACCT 150

S. commersonii - reference ATGTCACCACAAACAGAGACTAAAGCAAGTGTTGGATTCAAAGCTGGTGTTAAAGAGTACAAATTGACTTATTATACTCCTGAGTACCAAACCAAGGATACTGATATATTGGCAGCATTCCGAGTAACTCCTCAACCTGGAGTTCCACCT 150

S. commersonii - CLAW GAAGAAGCAGGGGCCGCGGTAGCTGCCGAATCTTCTACTGGTACATGGACAACTGTATGGACCGATGGACTTACCAGTCTTGATCGTTACAAAGGGCGATGCTACCGCATCGAGCGTGTTGTTGGAGAAAAAGATCAATATATTGCTTAT 300

S. commersonii - reference GAAGAAGCAGGGGCCGCGGTAGCTGCCGAATCTTCTACTGGTACATGGACAACTGTATGGACCGATGGACTTACCAGTCTTGATCGTTACAAAGGGCGATGCTACCGCATCGAGCGTGTTGTTGGAGAAAAAGATCAATATATTGCTTAT 300

S. commersonii - CLAW GTAGCTTACCCTTTAGACCTTTTTGAAGAAGGTTCCGTTACCAATATGCTTACTTCCATTGTAGGTAACGTATTTGGGTTCAAAGCCCTGCGCGCTCTACGTCTGGAAGATCTGCGAATCCCTGTTGCTTATGTTAAAACTTTCCAAGGT 450

S. commersonii - reference GTAGCTTACCCTTTAGACCTTTTTGAAGAAGGTTCCGTTACCAATATGCTTACTTCCATTGTAGGTAACGTATTTGGGTTCAAAGCCCTGCGCGCTCTACGTCTGGAAGATCTGCGAATCCCTGTTGCTTATGTTAAAACTTTCCAAGGT 450

S. commersonii - CLAW CCGCCTCATGGGATCCAAGTTGAAAGAGATAAATTGAACAAGTATGGTCGTCCCCTGTTGGGATGTACTATTAAACCTAAATTGGGGTTATCTGC-AAAAACTACGGTAGAGCTGTTTATGAATGTCTTCGCGGTGGACTTGATTTTACC 599

S. commersonii - reference CCGCCTCATGGGATCCAAGTTGAAAGAGATAAATTGAACAAGTATGGTCGTCCCCTGTTGGGATGTACTATTAAACCTAAATTGGGGTTATCTGCAAAAAACTACGGTAGAGCTGTTTATGAATGTCTTCGCGGTGGACTTGATTTTACC 600

S. commersonii - CLAW AAAGATGATGAGAACGTGAACTCACAACCATTTATGCGTTGGAGAGATCGTTTCTTATTTTGTGCCGAAGCAC-TTTTAAAGCACAGGCTGAAACAGGTGAAATCAAAGGGCATTACTTGAATGCTACTGCAGGTACATGCGAAGAAATG 748

S. commersonii - reference AAAGATGATGAGAACGTGAACTCACAACCATTTATGCGTTGGAGAGATCGTTTCTTATTTTGTGCCGAAGCACTTTTTAAAGCACAGGCTGAAACAGGTGAAATCAAAGGGCATTACTTGAATGCTACTGCAGGTACATGCGAAGAAATG 750

S. commersonii - CLAW ATGAAAAGAGCTATATTTGCTAGAGAATTGGGCAC-CCGATCGTAATGCATGACTACTTAAC--GGGGATTCACCGCAAATACTACCTTGGCTCATTATTGCCGAGATAATGGTCTACTTCTTCACATCCACCGTGCAATGCATGCGGTT 895

S. commersonii - reference ATGAAAAGAGCTATATTTGCTAGAGAATTGGGCACTCCGATCGTAATGCATGACTACTTAACGGGGGGATTCACCGCAAATACTACCTTGGCTCATTATTGCCGAGATAATGGTCTACTTCTTCACATCCACCGTGCAATGCATGCGGTT 900

S. commersonii - CLAW ATTGATAGACAGAAGAATCATGGTATGCACTTCCGGGTATTAGCAAAAGCGTTACGTATGTCTGGTGGAGATCATATTCACTCTGGTACCGTAGTAGGTAAACTTGAAGGTGAAAGAGACATAACTTTGGGCTTTGTTGATTTACTGCGT 1045

S. commersonii - reference ATTGATAGACAGAAGAATCATGGTATGCACTTCCGGGTATTAGCAAAAGCGTTACGTATGTCTGGTGGAGATCATATTCACTCTGGTACCGTAGTAGGTAAACTTGAAGGTGAAAGAGACATAACTTTGGGCTTTGTTGATTTACTGCGT 1050

S. commersonii - CLAW GATGATTTTATTGAACAAGATAGAAGTCGCGGTATTTATTTCACTCAAGATTGGGTCTCTTTACCAGGTGTTCTACCTGTGGCTTCAGGAGGTATTCACGTTTGGCATATGCCTGCTCTGACCGAGATCTTTGGGGATGATTCCGTACTA 1195

S. commersonii - reference GATGATTTTATTGAACAAGATAGAAGTCGCGGTATTTATTTCACTCAAGATTGGGTCTCTTTACCAGGTGTTCTACCTGTGGCTTCAGGAGGTATTCACGTTTGGCATATGCCTGCTCTGACCGAGATCTTTGGGGATGATTCCGTACTA 1200

S. commersonii - CLAW CAGTTCGGTGGAGGAACTTTAGGACATCCTTGGGGTAATGCGCCAGGTGCCGTAGCTAATCGAGTAGCTCTAGAAGCATGTGTAAAAGCTCGTAATGAAGGACGTGATCTTGCTCGGGAAGGTAATGAGATTATTCGCGAGGCTTCCAAA 1345

S. commersonii - reference CAGTTCGGTGGAGGAACTTTAGGACATCCTTGGGGTAATGCGCCAGGTGCCGTAGCTAATCGAGTAGCTCTAGAAGCATGTGTAAAAGCTCGTAATGAAGGACGTGATCTTGCTCGGGAAGGTAATGAGATTATTCGCGAGGCTTCCAAA 1350

S. commersonii - CLAW TGGAGCCCGGAACTAGCTGCTGCTTGTGAGGTATGGAAAGAGATCGTATTTAATTT 1401

S. commersonii - reference TGGAGCCCGGAACTAGCTGCTGCTTGTGAGGTATGGAAAGAGATCGTATTTAA--- 1403

V. radiata - CLAW ATGTCACCACAAACAGAGACTAAAGCAAGTGTTGGGTTCAAAGCTGGTGTTAAAGATTATAAATTGACTTATTATACTCCTGACTATGAAACCAAAGATACTGATATCTTGGCAGCATTCCGAGTAACTCCTCAACCTGGAGTTCCACCT 150

V. radiata - reference ATGTCACCACAAACAGAGACTAAAGCAAGTGTTGGGTTCAAAGCTGGTGTTAAAGATTATAAATTGACTTATTATACTCCTGACTATGAAACCAAAGATACTGATATCTTGGCAGCATTCCGAGTAACTCCTCAACCTGGAGTTCCACCT 150

V. radiata - CLAW GAAGAAGCAGGTGCTGCGGTAGCCGCCGAATCTTCTACTGGTACATGGACAACTGTGTGGACCGATGGGCTTACCAGTCTTGATCGTTACAAAGGACGATGCTATCACATCGAACCTGTTCCT-GGGAAGAAAGTCAATTTATTGCTTAC 299

V. radiata - reference GAAGAAGCAGGTGCTGCGGTAGCCGCCGAATCTTCTACTGGTACATGGACAACTGTGTGGACCGATGGGCTTACCAGTCTTGATCGTTACAAAGGACGATGCTATCACATCGAACCTGTTCCTGGGGAAGAAAGTCAATTTATTGCTTAC 300

V. radiata - CLAW GTAGCTTATCCCTTAGACCTTTTTGAAGAAGGTTCTGTTACTAACATGTTTACTTCTATTGTCGGTAATGTATTTGGGTTCAAGGCACTGCGTGCTCTACGTCTGGAGGATTTGCGAATCCCAACCGCTTATGTTAAAACTTTCCAAGGT 449

V. radiata - reference GTAGCTTATCCCTTAGACCTTTTTGAAGAAGGTTCTGTTACTAACATGTTTACTTCTATTGTCGGTAATGTATTTGGGTTCAAGGCACTGCGTGCTCTACGTCTGGAGGATTTGCGAATCCCAACCGCTTATGTTAAAACTTTCCAAGGT 450

V. radiata - CLAW CCGCCTCATGGCATCCAAGTTGAGAGAGATAAATTGAACAAGTATGGTCGTCCCCTATTAGGATGTACTATTAAACCTAAATTGGGGTTATCCGCTAAGAATTATGGTAGAGCTGTTTATGAATGTCTTCGT-GGGGACTTGATTTTACC 598

V. radiata - reference CCGCCTCATGGCATCCAAGTTGAGAGAGATAAATTGAACAAGTATGGTCGTCCCCTATTAGGATGTACTATTAAACCTAAATTGGGGTTATCCGCTAAGAATTATGGTAGAGCTGTTTATGAATGTCTTCGTGGGGGACTTGATTTTACC 600

V. radiata - CLAW AAAGATGATGAAAATGTGAATTCCCAACCATTTATGCGTTGGAGAGACCGTTTCTTATTTTGTGCTGAAGCGATTTATAAATCACAGGCTGAAACAGGTGAAATCAAAGGGCATTACTTGAATGCAACTGCGGGTACATGCGAAGAAATG 748

V. radiata - reference AAAGATGATGAAAATGTGAATTCCCAACCATTTATGCGTTGGAGAGACCGTTTCTTATTTTGTGCTGAAGCGATTTATAAATCACAGGCTGAAACAGGTGAAATCAAAGGGCATTACTTGAATGCAACTGCGGGTACATGCGAAGAAATG 750

V. radiata - CLAW ATGAAAAGAGCTATATTTGCCAGAGAATTAGGTGTTCCTATCATAATGCATGATTATTTAACAGG-GGATTCACTGCAAATACTAGCTTGGCTCATTATTGCCGAGATAATGGTCTACTTCTTCATATACATCGTGCAATGCATGCAGTT 897

V. radiata - reference ATGAAAAGAGCTATATTTGCCAGAGAATTAGGTGTTCCTATCATAATGCATGATTATTTAACAGGGGGATTCACTGCAAATACTAGCTTGGCTCATTATTGCCGAGATAATGGTCTACTTCTTCATATACATCGTGCAATGCATGCAGTT 900

V. radiata - CLAW ATCGACAGACAAAAGAATCATGGTATGCACTTTCGTGTATTAGCTAAAGCATTACGTTTATCTGGTGGAGATCATGTCCACTCCGGTACCGTAGTAGGTAAACTTGAA-GGGAAAGAGAAATCACTTTAGGTTTTGTTGACTTACTGCGT 1046

V. radiata - reference ATCGACAGACAAAAGAATCATGGTATGCACTTTCGTGTATTAGCTAAAGCATTACGTTTATCTGGTGGAGATCATGTCCACTCCGGTACCGTAGTAGGTAAACTTGAAGGGGAAAGAGAAATCACTTTAGGTTTTGTTGACTTACTGCGT 1050

V. radiata - CLAW GATGATTTTGTTG-AAAAGATCGAAGTCGTGGTATTTATTTCACTCAGGATTGGGTTTCTCTACCAGGTGTTATACCTGTTGCTTCGGGAGGTATTCACGTTTGGCATATGCCTGCTCTGACCGAAATCTTTGGAGATGATTCCGTACTT 1195

V. radiata - reference GATGATTTTGTTGAAAAAGATCGAAGTCGTGGTATTTATTTCACTCAGGATTGGGTTTCTCTACCAGGTGTTATACCTGTTGCTTCGGGAGGTATTCACGTTTGGCATATGCCTGCTCTGACCGAAATCTTTGGAGATGATTCCGTACTT 1200

V. radiata - CLAW CAATTTGGCGGAGGAACTTTAGGACACCCTT-GGGGAATGCACCAGGTGCTGTAGCTAATCGAGTAGCTCTTGAAGCATGTGTGAAGGCTCGAAATGAAGGACGTGATCTTGCTCGTGAAGGTAATGAAATTATCCGTGAGGCTAGCAAA 1344

V. radiata - reference CAATTTGGCGGAGGAACTTTAGGACACCCTTGGGGGAATGCACCAGGTGCTGTAGCTAATCGAGTAGCTCTTGAAGCATGTGTGAAGGCTCGAAATGAAGGACGTGATCTTGCTCGTGAAGGTAATGAAATTATCCGTGAGGCTAGCAAA 1350

V. radiata - CLAW TGGAGTCCTGAATTAGCTGCTGCTTGCGAAGTATGGAAGGAGATAAAATTTGAATTCGAAGCAGTGGATACTATTT 1420

V. radiata - reference TGGAGTCCTGAATTAGCTGCTGCTTGCGAAGTATGGAAGGAGATAAAATTTGAATTC------------------- 1407

CLAW-generated *Flye* assemblies using Pac-Bio data

C. reinhardtii - reference ATGGTTCCACAAACAGAAACTAAAGCAGGTGCTGGATTCAAAGCCGGTGTAAAAGACTACCGTTTAACATACTACACACCTGATTACGTAGTAAGAGATACTGATATTTTAGCTGCATTCCGTATGACTCCACAACTAGGTGTTCCACCT 150

C. reinhardtii - CLAW ATGGTTCCACAAACAGAAACTAAAGCAGGTGCTGGATTCAAAGCCGGTGTAAAAGACTACCGTTTAACATACTACACACCTGATTACGTAGTAAGAGATACTGATATTTTAGCTGCATTCCGTATGACTCCACAACCAGGTGTTCCACCT 150

C. reinhardtii - reference GAAGAATGTGGTGCTGCTGTAGCTGCTGAATCTTCAACAGGTACATGGACTACAGTATGGACTGACGGTTTAACAAGTCTTGACCGTTACAAAGGTCGTTGTTACGATATCGAACCAGTTCCGGGTGAAGACAACCAATACATTGCTTAC 300

C. reinhardtii - CLAW GAAGAATGTGGTGCTGCTGTAGCTGCTGAATCTTCAACAGGTACATGGACTACAGTATGGACTGACGGTTTAACAAGTCTTGACCGTTACAAAGGTCGTTGTTACGATATCGAACCAGTTCCGGGTGAAGACAACCAATACATTGCTTAC 300

C. reinhardtii - reference GTAGCTTACCCAATCGACTTATTCGAAGAAGGTTCAGTAACTAACATGTTCACTTCTATTGTAGGTAACGTATTCGGTTTCAAAGCTTTACGTGCTCTACGTCTTGAAGACCTTCGTATTCCACCTGCTTACGTTAAAACATTCGTAGGT 450

C. reinhardtii - CLAW GTAGCTTACCCAATCGACTTATTCGAAGAAGGTTCAGTAACTAACATGTTCACTTCTATTGTAGGTAACGTATTCGGTTTCAAAGCTTTACGTGCTCTACGTCTTGAAGACCTTCGTATTCCACCTGCTTACGTTAAAACATTCGTAGGT 450

C. reinhardtii - reference CCTCCACACGGTATTCAGGTAGAACGTGACAAATTAAACAAATATGGTCGTGGTCTTTTAGGTTGTACAATCAAACCTAAATTAGGTCTTTCAGCTAAAAACTACGGTCGTGCAGTTTATGAATGTTTACGTGGTGGTCTTGACTTTACT 600

C. reinhardtii - CLAW CCTCCACACGGTATTCAGGTAGAACGTGACAAATTAAACAAATATGGTCGTGGTCTTTTAGGTTGTACAATCAAACCTAAATTAGGTCTTTCAGCTAAAAACTACGGTCGTGCAGTTTATGAATGTTTACGTGGTGGTCTTGACTTTACT 600

C. reinhardtii - reference AAAGACGACGAAAACGTAAACTCACAACCATTCATGCGTTGGCGTGACCGTTTCCTTTTCGTTGCTGAAGCTATTTACAAAGCTCAAGCAGAAACAGGTGAAGTTAAAGGTCACTACTTAAACGCTACTGCTGGTACTTGTGAAGAAATG 750

C. reinhardtii - CLAW AAAGACGACGAAAACGTAAACTCACAACCATTCATGCGTTGGCGTGACCGTTTCCTTTTCGTTGCTGAAGCTATTTACAAAGCTCAAGCAGAAACAGGTGAAGTTAAAGGTCACTACTTAAACGCTACTGCTGGTACTTGTGAAGAAATG 750

C. reinhardtii - reference ATGAAACGTGCAGTATGTGCTAAAGAATTAGGTGTACCTATTATTATGCACGACTACTTAACAGGTGGTTTCACAGCTAACACTTCATTAGCTATCTACTGTCGTGACAACGGTCTTCTTCTACACATCCACCGTGCTATGCACGCGGTT 900

C. reinhardtii - CLAW ATGAAACGTGCAGTATGTGCTAAAGAATTAGGTGTACCTATTATTATGCACGACTACTTAACAGGTGGTTTCACAGCTAACACTTCATTAGCTATCTACTGTCGTGACAACGGTCTTCTTCTACACATCCACCGTGCTATGCACGCGGTT 900

C. reinhardtii - reference ATTGACCGTCAACGTAACCACGGTATTCACTTCCGTGTTCTTGCTAAAGCTCTTCGTATGTCTGGTGGTGACCACCTTCACTCTGGTACTGTTGTAGGTAAACTAGAAGGTGAACGTGAAGTTACTCTAGGTTTCGTAGACTTAATGCGT 1050

C. reinhardtii - CLAW ATTGACCGTCAACGTAACCACGGTATTCACTTCCGTGTTCTTGCTAAAGCTCTTCGTATGTCTGGTGGTGACCACCTTCACTCTGGTACTGTTGTAGGTAAACTAGAAGGTGAACGTGAAGTTACTCTAGGTTTCGTAGACTTAATGCGT 1050

C. reinhardtii - reference GATGACTACGTTGAAAAAGACCGTAGCCGTGGTATTTACTTCACTCAAGACTGGTGTTCAATGCCAGGTGTTATGCCAGTTGCTTCAGGCGGTATTCACGTATGGCACATGCCAGCTTTAGTTGAAATCTTCGGTGATGACGCATGTCTT 1200

C. reinhardtii - CLAW GATGACTACGTTGAAAAAGACCGTAGCCGTGGTATTTACTTCACTCAAGACTGGTGTTCAATGCCAGGTGTTATGCCAGTTGCTTCAGGTGGTATTCACGTATGGCACATGCCAGCTTTAGTTGAAATCTTCGGTGATGACGCATGTCTT 1200

C. reinhardtii - reference CAGTTCGGTGGTGGTACTCTAGGTCACCCTTGGGGTAACGCTCCAGGTGCTGCAGCTAACCGTGTAGCTCTTGAAGCTTGTACTCAAGCTCGTAACGAAGGTCGTGACCTTGCTCGTGAAGGTGGCGACGTAATTCGTTCAGCTTGTAAA 1350

C. reinhardtii - CLAW CAGTTCGGTGGTGGTACTCTAGGTCACCCTTGGGGTAACGCTCCAGGTGCTGCAGCTAACCGTGTAGCTCTTGAAGCTTGTACTCAAGCTCGTAACGAAGGTCGTGACCTTGCTCGTGAAGGTGGCGACGTAATTCGTTCAGCTTGTAAA 1350

C. reinhardtii - reference TGGTCTCCAGAACTTGCTGCTGCATGTGAAGTTTGGAAAGAAATTAAATTCGAATTTGATACTATTGACAAACTTTAA 1428

C. reinhardtii - CLAW TGGTCTCCAGAACTTGCTGCTGCATGTGAAGTTTGGAAAGAAATTAAATTCGAATTTGATACTATTGACAAACTTTAA 1428


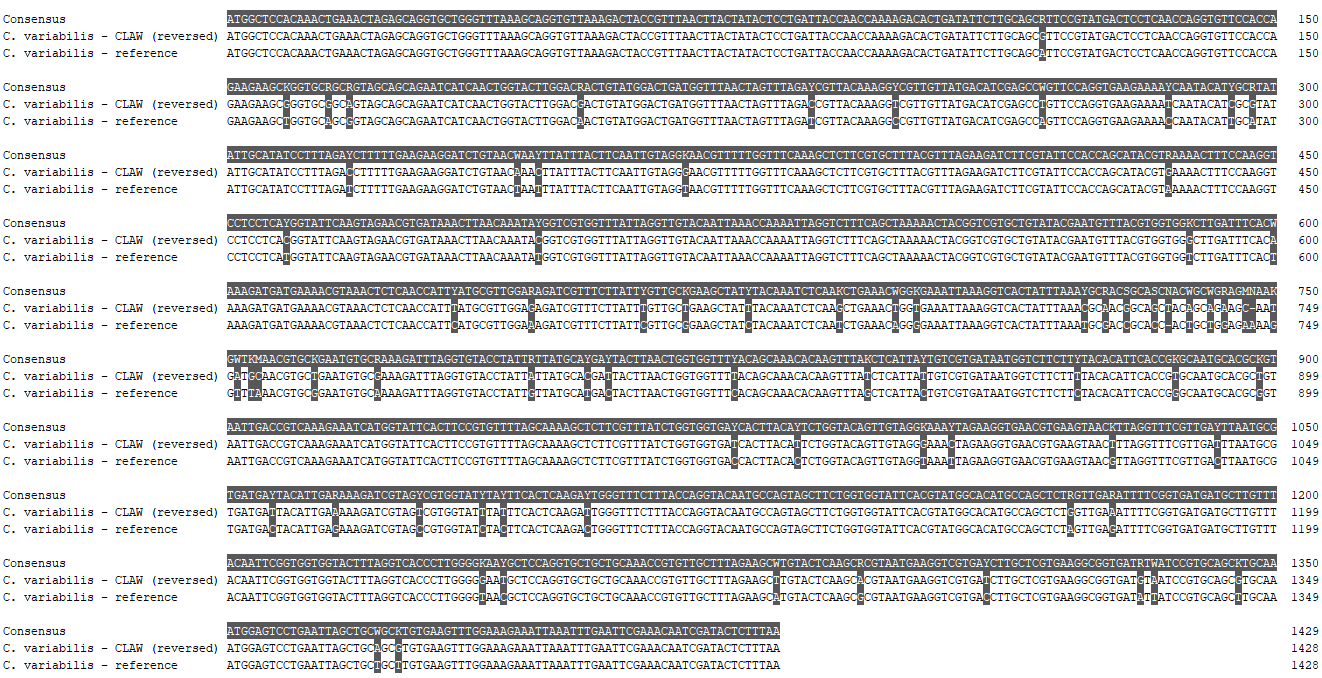


P. provasolii - CLAW (reversed) GGTGTACAAGACTACCGTCTTACTTACTACACTCCCGAGTACCAAGTAAAGGCTACTGATATTCTTGCGGCTTTCCGTATGACTCCCCAGCCTGGTGTACCCCCCGAGGAGTGTGGTGCAGCGGTAGCAGCTGAGTCCTCCACAGGTACT 150

P. provasolii - reference GGTGTACAAGACTACCGTCTTACTTACTACACTCCCGAGTACCAAGTAAAGGCTACTGATATTCTTGCGGCTTTCCGTATGACTCCCCAGCCTGGTGTACCCCCCGAGGAGTGTGGTGCAGCGGTAGCAGCTGAGTCCTCCACAGGTACT 150

P. provasolii - CLAW (reversed) TGGACTACTGTATGGACTGATGGTCTTACTTGTCTTGACAACTACAAGGGTCGTTGTTACGACCTCGAGCCCGTACCTGGTGAAGATAACCAGTACATTGCGTACATTGCTTACCCTATCGACCTCTTTGAGGAAGGTTCTGTAACTAAC 300

P. provasolii - reference TGGACTACTGTATGGACTGATGGTCTTACTTGTCTTGACAACTACAAGGGTCGTTGTTACGACCTCGAGCCCGTACCTGGTGAAGATAACCAGTACATTGCGTACATTGCTTACCCTATCGACCTCTTTGAGGAAGGTTCTGTAACTAAC 300

P. provasolii - CLAW (reversed) CTCTTTACTTCTATTGTAGGTAACGTATTTGGTTTCAAGGCTCTCCGTGCTCTTCGTCTTGAGGATCTTCGTATTCCTGCTGCATACGTAAAGACATTTGCTGGTCCTCCCCACGGTATTCAGGTTGAGCGTGATAAGCTTAACAAGTAC 450

P. provasolii - reference CTCTTTACTTCTATTGTAGGTAACGTATTTGGTTTCAAGGCTCTCCGTGCTCTTCGTCTTGAGGATCTTCGTATTCCTGCTGCATACGTAAAGACATTTGCTGGTCCTCCCCACGGTATTCAGGTTGAGCGTGATAAGCTTAACAAGTAC 450

P. provasolii - CLAW (reversed) GGTCGTCCTCTTCTCGGTTGTACTATTAAGCCTAAGCTCGGTCTCTCTGCTAAGAACTATGGTCGTGCAGTTTACGAGTGTCTCCGTGGTGGTCTTGACTTTACTAAGGATGATGAGAACGTAAACTCCCAGCCTTTCATGCGTTGGCGT 600

P. provasolii - reference GGTCGTCCTCTTCTCGGTTGTACTATTAAGCCTAAGCTCGGTCTCTCTGCTAAGAACTATGGTCGTGCAGTTTACGAGTGTCTCCGTGGTGGTCTTGACTTTACTAAGGATGATGAGAACGTAAACTCCCAGCCTTTCATGCGTTGGCGT 600

P. provasolii - CLAW (reversed) GATCGTTTCCTCTTCTGTGCTGAGGCTATTTACAAGGCACAAGGTGAAACTGGTGAGATTAAGGGTCACTACCTTAACGCAACTGCTGGTACGGCTGAGGAAATGCTCAAGCGTGCTGAGTTCGCTGTAGACCTTGGTATGCCCATTGTT 750

P. provasolii - reference GATCGTTTCCTCTTCTGTGCTGAGGCTATTTACAAGGCACAAGGTGAAACTGGTGAGATTAAGGGTCACTACCTTAACGCAACTGCTGGTACGGCTGAGGAAATGATGAAGCGTGCTGAGTTCGCTGTAGACCTTGGTATGCCCATTGTT 750

P. provasolii - CLAW (reversed) ATGCACGACTACCTTACTGGTGGTTTCACATCCAACACTACTCTTTCTAACTACTGTCGTGACAACGGTCTTCTTCTCCACATTCACCGTGCAATGCACGCGGTAATTGACCGTCAGCGTAACCACGGTATTCACTTCCGTGTTCTCGCG 900

P. provasolii - reference ATGCACGACTACCTTACTGGTGGTTTCACATCCAACACTACTCTTTCTAACTACTGTCGTGACAACGGTCTTCTTCTCCACATTCACCGTGCAATGCACGCGGTAATTGACCGTCAGCGTAACCACGGTATTCACTTCCGTGTTCTCGCG 900

P. provasolii - CLAW (reversed) AAGGCTCTCCGTCTTTCTGGTGGTGACCACCTTCACTCTGGTACTGTAGTAGGTAAGCTTGAGGGTGAGCGTGAAGTAACACTTGGTTTCGTAGATCTTATGCGTGACGACTTCGTTGAGAAGGACCGTAACCGTGGTATCTACTTTACT 1050

P. provasolii - reference AAGGCTCTCCGTCTTTCTGGTGGTGACCACCTTCACTCTGGTACTGTAGTAGGTAAGCTTGAGGGTGAGCGTGAAGTAACACTTGGTTTCGTAGATCTTATGCGTGACGACTTCGTTGAGAAGGACCGTAACCGTGGTATCTACTTTACT 1050

P. provasolii - CLAW (reversed) CAAGAGTGGTGTTCTATGGGTGGTGTACTCCCCGTAGCATCCGGTGGTATTCACGTATGGCACATGCCCGCACTCGTAGAGATCTTCGGTGATGACTCTGTACTCCAGTTTGGTGGTGGTACACTTGGTCACCCCTGGGGTAACGCTCCT 1200

P. provasolii - reference CAAGAGTGGTGTTCTATGGGTGGTGTAATGCCCGTAGCATCCGGTGGTATTCACGTATGGCACATGCCCGCACTCGTAGAGATCTTCGGTGATGACTCTGTACTCCAGTTTGGTGGTGGTACACTTGGTCACCCCTGGGGTAACGCTCCT 1200

P. provasolii - CLAW (reversed) GGTGCGGCTGCTAACCGTGTAGCTCTTGAGGCTTGTGTACAAGCTCGTAACGAAGGTCGTGACCTCGCTCGTGAGGGTGGTGACGTAATTCGTGCGGCTGCTAAGTGGAGCCCTGAGCTTGCTGCAGCTTGTGAGGTTTGGAAGGAGATT 1350

P. provasolii - reference GGTGCGGCTGCTAACCGTGTAGCTCTTGAGGCTTGTGTACAAGCTCGTAACGAAGGTCGTGACCTCGCTCGTGAGGGTGGTGACGTAATTCGTGCGGCTGCTAAGTGGAGCCCTGAGCTTGCTGCAGCTTGTGAGGTTTGGAAGGAGATT 1350

P. provasolii - CLAW (reversed) AAGTTCGAATTCGAGACTATTGATAAGCTCTAA 1383

P. provasolii - reference AAGTTCGAATTCGAGACTATTGATAAGCTCTAA 1383

A. officinalis - CLAW (reversed) ATGTCACCACAAACAGAGACTAAAGCAAGTGTTGGATTTAAAGCTGGTGTTAAAGATTACAGATTGACTTATTATACTCCTGATTACGAAACCAAAGATACTGATATCTTGGCAGCATTCCGAGTAACTGCTCAACCCGGAGTTCCCCCT 150

A. officinalis - reference ATGTCACCACAAACAGAGACTAAAGCAAGTGTTGGATTTAAAGCTGGTGTTAAAGATTACAGATTGACTTATTATACTCCTGATTACGAAACCAAAGATACTGATATCTTGGCAGCATTCCGAGTAACTGCTCAACCCGGAGTTCCCCCT 150

A. officinalis - CLAW (reversed) GAAGAAGCGGGCGCTGCGGTAGCTGCCGAATCTTCTACTGGTACATGGACAACTGTGTGGACTGATGGACTTACCAGTCTTGATCGTTACAAAGGACGATGCTACCACATCGAGCCCGTTATTGGGGAAGCAGAGCAATTTATTGCTTAT 300

A. officinalis - reference GAAGAAGCGGGCGCTGCGGTAGCTGCCGAATCTTCTACTGGTACATGGACAACTGTGTGGACTGATGGACTTACCAGTCTTGATCGTTACAAAGGACGATGCTACCACATCGAGCCCGTTATTGGGGAAGCAGAGCAATTTATTGCTTAT 300

A. officinalis - CLAW (reversed) GTAGCTTATCCTTTAGACCTTTTTGAAGAAGGTTCTGTTACTAACATGTTTACTTCCATTGTGGGTAATGTATTTGGTTTCAAAGCCCTACGAGCTCTACGTTTGGAGGATCTGCGAATTCCCCCTGCTTATTCCAAAACTTTCCAAGGC 450

A. officinalis - reference GTAGCTTATCCTTTAGACCTTTTTGAAGAAGGTTCTGTTACTAACATGTTTACTTCCATTGTGGGTAATGTATTTGGTTTCAAAGCCCTACGAGCTCTACGTTTGGAGGATCTGCGAATTCCCCCTGCTTATTCCAAAACTTTCCAAGGC 450

A. officinalis - CLAW (reversed) CCGCCTCATGGTATCCAAGTTGAAAGAGATAAATTGAACAAGTATGGTCGTCCCCTATTGGGATGTACTATTAAACCAAAATTGGGATTATCCGCAAAAAACTACGGTAGAGCAGTTTATGAATGTTTACGCGGTGGGCTTGATTTTACC 600

A. officinalis - reference CCGCCTCATGGTATCCAAGTTGAAAGAGATAAATTGAACAAGTATGGTCGTCCCCTATTGGGATGTACTATTAAACCAAAATTGGGATTATCCGCAAAAAACTACGGTAGAGCAGTTTATGAATGTTTACGCGGTGGGCTTGATTTTACC 600

A. officinalis - CLAW (reversed) AAGGATGATGAAAACGTGAACTCACAACCTTTTATGCGTTGGCGAGACCGTTTCTGTTTTTGTGCTGAAGCTCTTTATAAAGCACAAGCGGAAACAGGTGAAATCAAAGGACATTACTTGAATGCAACTGCAGGTACATGTGAAGAAATG 750

A. officinalis - reference AAGGATGATGAAAACGTGAACTCACAACCTTTTATGCGTTGGCGAGACCGTTTCTGTTTTTGTGCTGAAGCTCTTTATAAAGCACAAGCGGAAACAGGTGAAATCAAAGGACATTACTTGAATGCAACTGCAGGTACATGTGAAGAAATG 750

A. officinalis - CLAW (reversed) ATGAAAAGGGCCATATTTGCCAGAGAATTGGGAGTTCCCATCGTAATGCATGACTACTTAACTGGAGGATTTACTGCAAATACCACTTTGGCTCATTATTGCCGCGACAATGGTCTACTTCTTCACATCCACCGCGCAATGCATGCAGTT 900

A. officinalis - reference ATGAAAAGGGCCATATTTGCCAGAGAATTGGGAGTTCCCATCGTAATGCATGACTACTTAACTGGAGGATTTACTGCAAATACCACTTTGGCTCATTATTGCCGCGACAATGGTCTACTTCTTCACATCCACCGCGCAATGCATGCAGTT 900

A. officinalis - CLAW (reversed) ATTGATAGACAGAAAAATCATGGTATGCATTTTCGTGTACTAGCTAAAGCATTACGTATGTCTGGTGGAGATCATATTCACGCTGGTACAGTAGTAGGTAAACTGGAAGGGGAACGTGAGATGACTTTAGGTTTTGTTGATTTATTACGT 1050

A. officinalis - reference ATTGATAGACAGAAAAATCATGGTATGCATTTTCGTGTACTAGCTAAAGCATTACGTATGTCTGGTGGAGATCATATTCACGCTGGTACAGTAGTAGGTAAACTGGAAGGGGAACGTGAGATGACTTTAGGTTTTGTTGATTTATTACGT 1050

A. officinalis - CLAW (reversed) GATGATTATATTGAAAAAGACCGAAGTCGCGGTATTTTTTTCACTCAAGATTGGGTTTCTATGCCAGGTGTTATTCCCGTGGCTTCAGGGGGTATTCATGTTTGGCATATGCCTGCCCTAACCGAAATCTTTGGAGATGATTCCGTACTA 1200

A. officinalis - reference GATGATTATATTGAAAAAGACCGAAGTCGCGGTATTTTTTTCACTCAAGATTGGGTTTCTATGCCAGGTGTTATTCCCGTGGCTTCAGGGGGTATTCATGTTTGGCATATGCCTGCCCTAACCGAAATCTTTGGAGATGATTCCGTACTA 1200

A. officinalis - CLAW (reversed) CAGTTCGGTGGAGGAACTTTAGGACACCCTTGGGGAAATGCACCTGGTGCGGTAGCTAATCGGGTAGCTTTAGAAGCATGCGTACAAGCTCGTAATGAGGGACGTGATCTTGCTCGTGAGGGTAATGAGATTATCCGTGAAGCTGCCAAA 1350

A. officinalis - reference CAGTTCGGTGGAGGAACTTTAGGACACCCTTGGGGAAATGCACCTGGTGCGGTAGCTAATCGGGTAGCTTTAGAAGCATGCGTACAAGCTCGTAATGAGGGACGTGATCTTGCTCGTGAGGGTAATGAGATTATCCGTGAAGCTGCCAAA 1350

A. officinalis - CLAW (reversed) TGGAGCCCGGAACTAGCCGCTGC------------------------------------------------------- 1373

A. officinalis - reference TGGAGCCCGGAACTAGCCGCTGCTTGTGAAGTATGGAAAGAGATCAAATTCGAGTTCGAACCAGTAGATAAGATAGAT 1428

O. sativa - reference ATGTCACCACAAACAGAAACTAAAGCAAGTGTTGGATTTAAAGCTGGTGTTAAGGATTATAAATTGACTTACTACACCCCGGAGTACGAAACCAAGGACACTGATATCTTGGCAGCATTCCGAGTAACTCCTCAGCCGGGGGTTCCGCCC 150

O. sativa - CLAW ATGTCACCACAAACAGAAACTAAAGCAAGTGTTGGATTTAAAGCTGGTGTTAAGGATTATAAATTGACTTACTACACCCCGGAGTACGAAACCAAGGACACTGATATCTTGGCAGCATTCCGAGTAACTCCTCAGCCGGGGGTTCCGCCC 150

O. sativa - reference GAAGAAGCAGGGGCTGCAGTAGCTGCCGAATCTTCTACTGGTACATGGACAACTGTTTGGACTGATGGACTTACCAGTCTTGATCGTTACAAAGGCCGATGCTATCACATCGAGCCCGTTGTTGGGGAGGATAATCAATATATCGCTTAT 300

O. sativa - CLAW GAAGAAGCAGGGGCTGCAGTAGCTGCCGAATCTTCTACTGGTACATGGACAACTGTTTGGACTGATGGACTTACCAGTCTTGATCGTTACAAAGGCCGATGCTATCACATCGAGCCCGTTGTTGGGGAGGATAATCAATATATCGCTTAT 300

O. sativa - reference GTAGCTTATCCATTAGACCTATTTGAAGAGGGTTCTGTTACTAACATGTTTACTTCCATTGTGGGTAACGTATTTGGTTTCAAAGCCCTACGCGCTCTACGTCTGGAGGATCTGCGAATTCCCCCTACTTATTCAAAAACTTTCCAAGGT 450

O. sativa - CLAW GTAGCTTATCCATTAGACCTATTTGAAGAGGGTTCTGTTACTAACATGTTTACTTCCATTGTGGGTAACGTATTTGGTTTCAAAGCCCTACGCGCTCTACGTCTGGAGGATCTGCGAATTCCCCCTACTTATTCAAAAACTTTCCAAGGT 450

O. sativa - reference CCGCCTCATGGTATCCAAGTTGAAAGGGATAAGTTGAACAAATACGGTCGTCCTTTATTGGGATGTACTATTAAACCAAAATTGGGATTATCTGCAAAAAATTATGGTAGAGCATGTTATGAGTGTCTACGCGGTGGACTTGATTTTACC 600

O. sativa - CLAW CCGCCTCATGGTATCCAAGTTGAAAGGGATAAGTTGAACAAATACGGTCGTCCTTTATTGGGATGTACTATTAAACCAAAATTGGGATTATCTGCAAAAAATTATGGTAGAGCATGTTATGAGTGTCTACGCGGTGGACTTGATTTTACC 600

O. sativa - reference AAAGATGATGAAAACGTAAACTCACAACCATTTATGCGTTGGAGGGACCGTTTTGTCTTTTGTGCCGAAGCTATTTATAAATCACAGGCCGAAACCGGTGAAATTAAGGGGCATTACTTGAATGCGACTGCAGGTACATGCGAAGAAATG 750

O. sativa - CLAW AAAGATGATGAAAACGTAAACTCACAACCATTTATGCGTTGGAGGGACCGTTTTGTCTTTTGTGCCGAAGCTATTTATAAATCACAGGCCGAAACCGGTGAAATTAAGGGGCATTACTTGAATGCGACTGCAGGTACATGCGAAGAAATG 750

O. sativa - reference ATTAAAAGAGCTGTATTTGCGAGGGAATTAGGGGTTCCTATTGTAATGCATGACTACTTAACCGGGGGATTCACCGCAAATACTAGTTTGGCTCATTATTGCCGCGACAACGGCCTACTTCTTCACATTCACCGAGCAATGCATGCAGTT 900

O. sativa - CLAW ATTAAAAGAGCTGTATTTGCGAGGGAATTAGGGGTTCCTATTGTAATGCATGACTACTTAACCGGGGGATTCACCGCAAATACTAGTTTGGCTCATTATTGCCGCGACAACGGCCTACTTCTTCACATTCACCGAGCAATGCATGCAGTT 900

O. sativa - reference ATTGATAGACAGAAAAATCATGGTATGCATTTCCGTGTATTAGCTAAAGCATTGCGTATGTCTGGGGGAGATCATATCCACGCTGGTACAGTAGTAGGTAAGTTAGAAGGGGAACGCGAAATGACTTTAGGTTTTGTTGATTTATTGCGC 1050

O. sativa - CLAW ATTGATAGACAGAAAAATCATGGTATGCATTTCCGTGTATTAGCTAAAGCATTGCGTATGTCTGGGGGAGATCATATCCACGCTGGTACAGTAGTAGGTAAGTTAGAAGGGGAACGCGAAATGACTTTAGGTTTTGTTGATTTATTGCGC 1050

O. sativa - reference GATGATTTTATTGAAAAAGATCGTGCTCGCGGTATCTTTTTCACTCAGGACTGGGTATCCATGCCAGGTGTTATACCGGTGGCTTCAGGGGGTATTCATGTTTGGCATATGCCAGCTCTGACCGAAATCTTTGGAGATGATTCTGTATTG 1200

O. sativa - CLAW GATGATTTTATTGAAAAAGATCGTGCTCGCGGTATCTTTTTCACTCAGGACTGGGTATCCATGCCAGGTGTTATACCGGTGGCTTCAGGGGGTATTCATGTTTGGCATATGCCAGCTCTGACCGAAATCTTTGGAGATGATTCTGTATTG 1200

O. sativa - reference CAATTTGGTGGAGGAACTTTAGGACATCCTTGGGGTAATGCACCTGGTGCAGCAGCTAATCGGGTGGCTTTAGAAGCCTGTGTACAAGCTCGTAACGAAGGGCGCGATCTTGCTCGTGAAGGTAATGAAATTATCCGATCAGCTTGCAAA 1350

O. sativa - CLAW CAATTTGGTGGAGGAACTTTAGGACATCCTTGGGGTAATGCACCTGGTGCAGCAGCTAATCGGGTGGCTTTAGAAGCCTGTGTACAAGCTCGTAACGAAGGGCGCGATCTTGCTCGTGAAGGTAATGAAATTATCCGATCAGCTTGCAAA 1350

O. sativa - reference TGGAGTCCTGAACTAGCCGCAGCTTGTGAAATATGGAAAGCGATCAAATT 1400

O. sativa - CLAW TGGAGTCCTGAACTAGCCGCAGCTTGTGAAATATGGAAAGCGATCAAATT 1400

A. sinensis - CLAW ATGTCACCACAAACAGAGACTAAAGCAAGTGTTGGATTCAAAGCTGGTGTTAAAGAGTATAAATTGACTTATTATACTCCTGAATATGAAACCAAAGATACTGATATCTTGGCAGCATTCCGAGTAACTCCTCAACCTGGAGTTCCGCCT 150

A. sinensis - reference ATGTCACCACAAACAGAGACTAAAGCAAGTGTTGGATTCAAAGCTGGTGTTAAAGAGTATAAATTGACTTATTATACTCCTGAATATGAAACCAAAGATACTGATATCTTGGCAGCATTCCGAGTAACTCCTCAACCTGGAGTTCCGCCT 150

A. sinensis - CLAW GAGGAAGCAGGGGCTGCGGTAGCTGCTGAATCTTCTACTGGTACATGGACAACTGTGTGGACCGACGGGCTTACCAGCCTTGATCGTTACAAAGGGCGATGCTACCACATCGAGCCCGTTGCTGGGGAAGAAAATCAATATATATGTTAT 300

A. sinensis - reference GAGGAAGCAGGGGCTGCGGTAGCTGCTGAATCTTCTACTGGTACATGGACAACTGTGTGGACCGACGGGCTTACCAGCCTTGATCGTTACAAAGGGCGATGCTACCACATCGAGCCCGTTGCTGGGGAAGAAAATCAATATATATGTTAT 300

A. sinensis - CLAW GTAGCTTACCCCTTAGACCTTTTTGAAGAGGGTTCTGTTACTAACATGTTTACTTCCATTGTTGGTAATGTATTTGGGTTCAAAGCCCTGCGCGCTCTACGTCTAGAAGATCTGCGAATCCCTACTTCTTATATTAAAACTTTCCAAGGT 450

A. sinensis - reference GTAGCTTACCCCTTAGACCTTTTTGAAGAGGGTTCTGTTACTAACATGTTTACTTCCATTGTTGGTAATGTATTTGGGTTCAAAGCCCTGCGCGCTCTACGTCTAGAAGATCTGCGAATCCCTACTTCTTATATTAAAACTTTCCAAGGT 450

A. sinensis - CLAW CCGCCTCATGGCATCCAAGTTGAAAGAGATAAATTGAACAAGTACGGCCGTCCCCTATTGGGATGTACTATTAAACCTAAATTGGGGTTATCCGCTAAAAACTACGGTAGAGCGGTTTATGAATGTCTACGTGGTGGACTTGATTTTACC 600

A. sinensis - reference CCGCCTCATGGCATCCAAGTTGAAAGAGATAAATTGAACAAGTACGGCCGTCCCCTATTGGGATGTACTATTAAACCTAAATTGGGGTTATCCGCTAAAAACTACGGTAGAGCGGTTTATGAATGTCTACGTGGTGGACTTGATTTTACC 600

A. sinensis - CLAW AAAGATGATGAGAATGTGAACTCCCAACCATTTATGCGTTGGAGAGACCGTTTCTTATTTTGTGCCGAAGCAATTTATAAAGCACAGGCTGAAACAGGTGAAATCAAAGGGCATTACTTGAATGCTACTGCGGGTACATGCGAAGAAATG 750

A. sinensis - reference AAAGATGATGAGAATGTGAACTCCCAACCATTTATGCGTTGGAGAGACCGTTTCTTATTTTGTGCCGAAGCAATTTATAAAGCACAGGCTGAAACAGGTGAAATCAAAGGGCATTACTTGAATGCTACTGCGGGTACATGCGAAGAAATG 750

A. sinensis - CLAW ATCAAAAGGGCTGTATTTGCCAGAGAATTAGGAGCTCCTATCGTAATGCATGACTATTTAACGGGGGGATTCACGGCAAATACTAGCTTGGCTCATTATTGCCGAGATAATGGTCTCCTTCTTCACATCCATCGCGCAATGCACGCAGTT 900

A. sinensis - reference ATCAAAAGGGCTGTATTTGCCAGAGAATTAGGAGCTCCTATCGTAATGCATGACTATTTAACGGGGGGATTCACGGCAAATACTAGCTTGGCTCATTATTGCCGAGATAATGGTCTCCTTCTTCACATCCATCGCGCAATGCACGCAGTT 900

A. sinensis - CLAW ATTGATAGACAGAAGAATCACGGTATGCACTTCCGTGTACTAGCTAAAGCCTTACGTATGTCTGGTGGAGATCATATTCACGCTGGTACAGTAGTAGGTAAACTTGAAGGAGAAAGAGACATAACTTTGGGTTTTGTTGATTTACTACGT 1050

A. sinensis - reference ATTGATAGACAGAAGAATCACGGTATGCACTTCCGTGTACTAGCTAAAGCCTTACGTATGTCTGGTGGAGATCATATTCACGCTGGTACAGTAGTAGGTAAACTTGAAGGAGAAAGAGACATAACTTTGGGTTTTGTTGATTTACTACGT 1050

A. sinensis - CLAW GATGATTTTATTGAAAAAGATAGAAGCCGTGGTATTTATTTCACTCAAGATTGGGTCTCTCTACCAGGTGTTATACCGGTAGCTTCGGGGGGTATTCACGTTTGGCATATGCCTGCTTTGACCGAGATCTTTGGTGATGATGCCGTACTA 1200

A. sinensis - reference GATGATTTTATTGAAAAAGATAGAAGCCGTGGTATTTATTTCACTCAAGATTGGGTCTCTCTACCAGGTGTTATACCGGTAGCTTCGGGGGGTATTCACGTTTGGCATATGCCTGCTTTGACCGAGATCTTTGGTGATGATGCCGTACTA 1200

A. sinensis - CLAW CAATTTGGTGGAGGAACTTTAGGACACCCTTGGGGAAATGCACCGGGTGCCGTCGCTAATCGAGTAGCTCTAGAAGCATGTGTACAAGCTCGTAATGAGGGACGTGATCTTGCTCGCGAGGGTAATGAAATTATCCGTACGGCTAGCAAA 1350

A. sinensis - reference CAATTTGGTGGAGGAACTTTAGGACACCCTTGGGGAAATGCACCGGGTGCCGTCGCTAATCGAGTAGCTCTAGAAGCATGTGTACAAGCTCGTAATGAGGGACGTGATCTTGCTCGCGAGGGTAATGAAATTATCCGTACGGCTAGCAAA 1350

A. sinensis - CLAW TGGAGTCCTGAACTAGCTGCTGCTTGTGAAGTATGGAAAGAGATCAAATTTGAATTCC------------ 1408

A. sinensis - reference TGGAGTCCTGAACTAGCTGCTGCTTGTGAAGTATGGAAAGAGATCAAATTTGAATTCCAAGCAGTGGATA 1420

C. sativa - reference ATGTCACCACAAACAGAGACTAAAGCAAGTGTTGGATTCAAAGCTGGTGTTAAAGATTATAAATTGACTTATTACACTCCGGAATATCAAACCAAAGATACTGATATCTTGGCAGCATTTCGAGTAACTCCTCAACCTGGAGTTCCCCCT 150

C. sativa - CLAW ATGTCACCACAAACAGAGACTAAAGCAAGTGTTGGATTCAAAGCTGGTGTTAAAGATTATAAATTGACTTATTACACTCCGGAATATCAAACCAAAGATACTGATATCTTGGCAGCATTTCGAGTAACTCCTCAACCTGGAGTTCCCCCT 150

C. sativa - reference GAAGAAGCAGGGGCTGCGGTAGCTGCTGAATCTTCTACTGGTACATGGACAACTGTATGGACTGATGGGCTTACCAGCCTTGATCGCTACAAAGGTCGATGCTACCACATCGAGCCCGTTGCTGGAGAAGAAAATCAATTTATTGCTTAT 300

C. sativa - CLAW GAAGAAGCAGGGGCTGCGGTAGCTGCTGAATCTTCTACTGGTACATGGACAACTGTATGGACTGATGGGCTTACCAGCCTTGATCGCTACAAAGGTCGATGCTACCACATCGAGCCCGTTGCTGGAGAAGAAAATCAATTTATTGCTTAT 300

C. sativa - reference GTAGCTTATCCCTTAGACCTTTTTGAAGAAGGTTCTGTTACTAACATGTTTACTTCCATTGTGGGTAATGTATTTGGGTTCAAGGCCCTGCGCGCTCTACGTCTGGAAGATTTGAGAATCCCTACTTCTTATACTAAAACTTTCCAAGGT 450

C. sativa - CLAW GTAGCTTATCCCTTAGACCTTTTTGAAGAAGGTTCTGTTACTAACATGTTTACTTCCATTGTGGGTAATGTATTTGGGTTCAAGGCCCTGCGCGCTCTACGTCTGGAAGATTTGAGAATCCCTACTTCTTATACTAAAACTTTCCAAGGT 450

C. sativa - reference CCGCCTCATGGGATCCAAGTTGAGAGAGATAAATTGAACAAGTATGGTCGCCCACTATTGGGATGTACTATTAAACCTAAATTGGGGTTATCCGCTAAGAATTACGGTAGAGCAGTTTATGAATGTCTTCGCGGTGGACTTGATTTTACC 600

C. sativa - CLAW CCGCCTCATGGGATCCAAGTTGAGAGAGATAAATTGAACAAGTATGGTCGCCCACTATTGGGATGTACTATTAAACCTAAATTGGGGTTATCCGCTAAGAATTACGGTAGAGCAGTTTATGAATGTCTTCGCGGTGGACTTGATTTTACC 600

C. sativa - reference AAAGATGATGAGAACGTAAATTCCCAACCATTTATGCGTTGGAGAGACCGTTTCTTATTTTGTGCAGAAGCAATTTATAAATCACAGTCTGAAACAGGGGAAATCAAAGGACATTACTTGAATGCTACTGCAGGTACATGTGAAGAAATG 750

C. sativa - CLAW AAAGATGATGAGAACGTAAATTCCCAACCATTTATGCGTTGGAGAGACCGTTTCTTATTTTGTGCAGAAGCAATTTATAAATCACAGTCTGAAACAGGGGAAATCAAAGGACATTACTTGAATGCTACTGCAGGTACATGTGAAGAAATG 750

C. sativa - reference ATGAAAAGGGCTGTATTTGCCAGAGAATTGGGAGTTCCTATCGTAATGCATGATTACTTAACAGGAGGATTCACTGCAAATACTAGTCTGGCTCATTATTGTCGAGATAATGGTCTACTTCTTCACATCCACCGTGCAATGCATGCGGTT 900

C. sativa - CLAW ATGAAAAGGGCTATATTTGCCAGAGAATTGGGAGTTCCTATCGTAATGCATGATTACTTAACAGGAGGATTCACTGCAAATACTAGTCTGGCTCATTATTGTCGAGATAATGGTCTACTTCTTCACATCCACCGTGCAATGCATGCGGTT 900

C. sativa - reference ATTGATAGACAAAAGAATCATGGTATACACTTCCGTGTACTAGCTAAAGCGTTACGTATGTCTGGTGGAGATCATATCCATTCAGGTACTGTAGTAGGTAAACTTGAAGGGGAAAGAGAAATCACTTTAGGCTTTGTTGATTTACTACGT 1050

C. sativa - CLAW ATTGATAGACAAAAGAATCATGGTATACACTTCCGTGTACTAGCTAAAGCGTTACGTATGTCTGGTGGAGATCATATCCATTCAGGTACTGTAGTAGGTAAACTTGAAGGGGAAAGAGAAATCACTTTAGGCTTTGTTGATTTACTACGT 1050

C. sativa - reference GATGATTTTATTGAAAAAGATCGAAGCCGTGGTATTTATTTCACTCAAGATTGGGTCTCTCTACCAGGTGTTCTGCCTGTGGCTTCAGGGGGTATTCACGTTTGGCATATGCCTGCTTTGACCGAGATCTTTGGAGATGATTCCGTACTA 1200

C. sativa - CLAW GATGATTTTATTGAAAAAGATCGAAGCCGTGGTATTTATTTCACTCAAGATTGGGTCTCTCTACCAGGTGTTCTGCCTGTGGCTTCAGGGGGTATTCACGTTTGGCATATGCCTGCTTTGACCGAGATCTTTGGAGATGATTCCGTACTA 1200

C. sativa - reference CAATTTGGTGGAGGAACTTTAGGACATCCTTGGGGAAATGCACCCGGTGCTGTCGCTAATCGAGTAGCTCTAGAAGCATGTGTACAAGCTCGTAATGAGGGACGTGATCTTGCTCGTGAGGGTAATGAAATTATTCGTGAGGCTTGTAAA 1350

C. sativa - CLAW CAATTTGGTGGAGGAACTTTAGGACATCCTTGGGGAAATGCACCCGGTGCTGTCGCTAATCGAGTAGCTCTAGAAGCATGTGTACAAGCTCGTAATGAGGGACGTGATCTTGCTCGTGAGGGTAATGAAATTATTCGTGAGGCTTGTAAA 1350

C. sativa - reference TGGAGTCCTGAACTAGCTGCTGCTTGTGAAGTTTGGAAGGAAATCAAATTT 1401

C. sativa - CLAW TGGAGTCCTGAACTAGCTGCTGCTTGTGAAGTTTGGAAGGAAATCAAATTT 1401

L. sativus - CLAW (reversed) ATGTCACCACAAACAGAAACTAAAGCAAAGGTTGGGTTCAAAGCTGGTGTTAAAGATTATAAATTGACTTATTATACTCCTGACTATCAAACCAAAGATACTGATATCTTGGCAGCATTCCGAGTAACTCCTCAACCTGGAGTTCCGCCT 150

L. sativus - reference ATGTCACCACAAACAGAAACTAAAGCAAAGGTTGGGTTCAAAGCTGGTGTTAAAGATTATAAATTGACTTATTATACTCCTGACTATCAAACCAAAGATACTGATATCTTGGCAGCATTCCGAGTAACTCCTCAACCTGGAGTTCCGCCT 150

L. sativus - CLAW (reversed) GAAGAAGCAGGTGCAGCGGTAGCTGCAGAATCTTCCACTGGTACATGGACAACTGTGTGGACCGATGGACTTACCAGCCTTGATCGTTATAAAGGACGCTGCTACGAGATCGAGCCTGTTCCTGGAGAAGATAATCAATTTATTGCTTAT 300

L. sativus - reference GAAGAAGCAGGTGCAGCGGTAGCTGCAGAATCTTCCACTGGTACATGGACAACTGTGTGGACCGATGGACTTACCAGCCTTGATCGTTATAAAGGACGCTGCTACGAGATCGAGCCTGTTCCTGGAGAAGATAATCAATTTATTGCTTAT 300

L. sativus - CLAW (reversed) GTAGCTTATCCCTTAGACCTTTTTGAAGAAGGTTCTGTTACTAACATGTTTACCTCCATTGTAGGTAATGTATTTGGGTTCAAGGCCTTGCGCGCTCTACGTCTGGAAGATTTGCGAATCCCTAATGCTTATGTTAAAACTTTCCAAGGT 450

L. sativus - reference GTAGCTTATCCCTTAGACCTTTTTGAAGAAGGTTCTGTTACTAACATGTTTACCTCCATTGTAGGTAATGTATTTGGGTTCAAGGCCTTGCGCGCTCTACGTCTGGAAGATTTGCGAATCCCTAATGCTTATGTTAAAACTTTCCAAGGT 450

L. sativus - CLAW (reversed) CCTCCTCACGGAATCCAAGTTGAGAGAGATAAATTGAACAAGTATGGACGTCCCCTATTGGGATGTACTATAAAACCCAAATTGGGTTTATCAGCTAAGAATTATGGTAGAGCAGTTTATGAATGTCTCCGCGGGGGACTTGATTTTACC 600

L. sativus - reference CCTCCTCACGGAATCCAAGTTGAGAGAGATAAATTGAACAAGTATGGACGTCCCCTATTGGGATGTACTATAAAACCCAAATTGGGTTTATCAGCTAAGAATTATGGTAGAGCAGTTTATGAATGTCTCCGCGGGGGACTTGATTTTACC 600

L. sativus - CLAW (reversed) AAAGATGATGAAAATGTGAACTCCCAACCATTTATGCGTTGGAGAGACCGTTTCTTATTTTGTGCCGAAGCAATTTATAAATCACAGGCCGAAACAGGGGAAATCAAAGGACATTATTTGAATGCTACTGCGGGTACATGTGAAGAAATG 750

L. sativus - reference AAAGATGATGAAAATGTGAACTCCCAACCATTTATGCGTTGGAGAGACCGTTTCTTATTTTGTGCCGAAGCAATTTATAAATCACAGGCCGAAACAGGGGAAATCAAAGGACATTATTTGAATGCTACTGCGGGTACATGTGAAGAAATG 750

L. sativus - CLAW (reversed) CTAAAAAGAGCTGTATTTGCTAGAGAATTGGGCGTTCCTATCGTAATGCATGACTACTTAACAGGTGGATTCACTGCAAATACTACCCTGTCTCACTATTGCCGCGATAATGGTCTACTTCTTCATATCCACCGTGCAATGCATGCAGTT 900

L. sativus - reference CTAAAAAGAGCTGTATTTGCTAGAGAATTGGGCGTTCCTATCGTAATGCATGACTACTTAACAGGTGGATTCACTGCAAATACTACCCTGTCTCACTATTGCCGCGATAATGGTCTACTTCTTCATATCCACCGTGCAATGCATGCAGTT 900

L. sativus - CLAW (reversed) ATCGATAGACAAAAAAATCATGGTATGCACTTTCGTGTATTAGCTAAAGCCTTACGTTTGTCTGGTGGAGATCATATTCACGCTGGTACTGTAGTAGGTAAACTTGAAGGAGAAAGGGAGATTACTTTAGGTTTTGTTGATTTACTACGT 1050

L. sativus - reference ATCGATAGACAAAAAAATCATGGTATGCACTTTCGTGTATTAGCTAAAGCCTTACGTTTGTCTGGTGGAGATCATATTCACGCTGGTACTGTAGTAGGTAAACTTGAAGGAGAAAGGGAGATTACTTTAGGTTTTGTTGATTTACTACGT 1050

L. sativus - CLAW (reversed) GATGATTATATTGAAAAAGATCGAAGTCGCGGTATTTATTTCACTCAGGATTGGGTTTCTTTACCAGGTGTTATCCCTGTTGCTTCAGGGGGTATTCACGTTTGGCATATGCCTGCTCTGACCGAGATATTTGGAGATGATTCTGTACTC 1200

L. sativus - reference GATGATTATATTGAAAAAGATCGAAGTCGCGGTATTTATTTCACTCAGGATTGGGTTTCTTTACCAGGTGTTATCCCTGTTGCTTCAGGGGGTATTCACGTTTGGCATATGCCTGCTCTGACCGAGATATTTGGAGATGATTCTGTACTC 1200

L. sativus - CLAW (reversed) CAATTCGGTGGAGGAACTTTAGGACACCCTTGGGGAAATGCACCTGGTGCCGTAGCGAATCGAGTAGCTCTGGAAGCATGTGTACAAGCTCGGAATGAGGGACGTGATCTTGCTCGCGAGGGTAATGCAATTATCCGTCAAGCTTGCAAA 1350

L. sativus - reference CAATTCGGTGGAGGAACTTTAGGACACCCTTGGGGAAATGCACCTGGTGCCGTAGCGAATCGAGTAGCTCTGGAAGCATGTGTACAAGCTCGGAATGAGGGACGTGATCTTGCTCGCGAGGGTAATGCAATTATCCGTCAAGCTTGCAAA 1350

L. sativus - CLAW (reversed) TGGAGTCCTGAATTAGCTGCTGCTTGTGAAGTCTGGAAGGAAATCAAATTTGAATTCCCAGCAATGGATA 1420

L. sativus - reference TGGAGTCCTGAATTAGCTGCTGCTTGTGAAGTCTGGAAGGAAATCAAATTTGAATTCCCAGCAATGGATA 1420

M. truncatula - CLAW (reversed) ATGTCACCACAAACAGAAACTAAAGCAACGGTTGGGTTCAAAGCTGGTGTTAAAGATTATCGATTGACTTATTATACTCCTGACTATGAAACCAAAGATACTGATATCTTGGCAGCATTCCGAGTAAGTCCTCAACCTGGAGTTCCGGCT 150

M. truncatula - reference ATGTCACCACAAACAGAAACTAAAGCAACGGTTGGGTTCAAAGCTGGTGTTAAAGATTATCGATTGACTTATTATACTCCTGACTATGAAACCAAAGATACTGATATCTTGGCAGCATTCCGAGTAAGTCCTCAACCTGGAGTTCCGGCT 150

M. truncatula - CLAW (reversed) GAAGAAGCAGGTGCAGCGGTAGCTGCCGAATCTTCCACTGGGACATGGACAACCGTGTGGACCGATGGACTTACCAGTCTTGATCGTTATAAAGGACGCTGCTACCACATCGAACCTGTTGCTGGAGAAGAGAGTCAATTTATTGCTTAT 300

M. truncatula - reference GAAGAAGCAGGTGCAGCGGTAGCTGCCGAATCTTCCACTGGGACATGGACAACCGTGTGGACCGATGGACTTACCAGTCTTGATCGTTATAAAGGACGCTGCTACCACATCGAACCTGTTGCTGGAGAAGAGAGTCAATTTATTGCTTAT 300

M. truncatula - CLAW (reversed) GTAGCTTATCCCTTAGACCTTTTTGAAGAAGGTTCTGTTACTAACATGTTTACCTCCATTGTAGGTAATGTATTTGGGTTCAAGGCCTTGCGTGCTCTACGTCTGGAAGATTTGCGAATCCCCGTTGCTTATGTTAAAACTTTCCAAGGT 450

M. truncatula - reference GTAGCTTATCCCTTAGACCTTTTTGAAGAAGGTTCTGTTACTAACATGTTTACCTCCATTGTAGGTAATGTATTTGGGTTCAAGGCCTTGCGTGCTCTACGTCTGGAAGATTTGCGAATCCCCGTTGCTTATGTTAAAACTTTCCAAGGT 450

M. truncatula - CLAW (reversed) CCTCCTCACGGAATCCAAGTTGAGAGAGATAAATTGAACAAATATGGACGTCCCCTATTGGGATGTACTATTAAACCTAAATTGGGTTTATCCGCTAAAAATTACGGTAGAGCAGTTTATGAATGTCTACGTGGTGGACTTGATTTTACC 600

M. truncatula - reference CCTCCTCACGGAATCCAAGTTGAGAGAGATAAATTGAACAAATATGGACGTCCCCTATTGGGATGTACTATTAAACCTAAATTGGGTTTATCCGCTAAAAATTACGGTAGAGCAGTTTATGAATGTCTACGTGGTGGACTTGATTTTACC 600

M. truncatula - CLAW (reversed) AAAGATGATGAAAATGTGAACTCCCAACCATTTATGCGTTGGAGAGACCGTTTCTTATTTTGTGCCGAAGCTATTTATAAAGCACAGGCCGAAACTGGTGAAATCAAAGGACATTATTTGAATGCTACTGCGGGCACCTGTGAAGACATG 750

M. truncatula - reference AAAGATGATGAAAATGTGAACTCCCAACCATTTATGCGTTGGAGAGACCGTTTCTTATTTTGTGCCGAAGCTATTTATAAAGCACAGGCCGAAACTGGTGAAATCAAAGGACATTATTTGAATGCTACTGCGGGCACCTGTGAAGACATG 750

M. truncatula - CLAW (reversed) ATGAAAAGAGCTGTATTTGCTAGAGAATTGGGCGTGCCTATCGTAATGCATGACTACTTAACCGGTGGATTCACTGCAAATACTACCTTGGCTCACTATTGCCGCGATAATGGTCTACTTCTTCATATCCACCGTGCAATGCATGCAGTT 900

M. truncatula - reference ATGAAAAGAGCTGTATTTGCTAGAGAATTGGGCGTGCCTATCGTAATGCATGACTACTTAACCGGTGGATTCACTGCAAATACTACCTTGGCTCACTATTGCCGCGATAATGGTCTACTTCTTCATATCCACCGTGCAATGCATGCAGTT 900

M. truncatula - CLAW (reversed) ATTGATAGACAGAAAAATCATGGTATGCACTTTCGTGTATTAGCTAAAGCGTTACGTATGTCAGGTGGAGATCATATTCACGCTGGTACTGTAGTAGGTAAACTGGAAGGAGAAAGGGATATTACTTTAGGTTTTGTTGATTTACTACGT 1050

M. truncatula - reference ATTGATAGACAGAAAAATCATGGTATGCACTTTCGTGTATTAGCTAAAGCGTTACGTATGTCAGGTGGAGATCATATTCACGCTGGTACTGTAGTAGGTAAACTGGAAGGAGAAAGGGATATTACTTTAGGTTTTGTTGATTTACTACGT 1050

M. truncatula - CLAW (reversed) GATGATTTTGTTGAAAAAGATAGAAGTCGCGGTATTTTTTTCACTCAGGATTGGGTTTCTTTACCTGGTGTTCTGCCTGTTGCTTCAGGTGGTATTCATGTTTGGCATATGCCTGCTCTGACCGAGATATTTGGAGATGATTCTGTACTT 1200

M. truncatula - reference GATGATTTTGTTGAAAAAGATAGAAGTCGCGGTATTTTTTTCACTCAGGATTGGGTTTCTTTACCTGGTGTTCTGCCTGTTGCTTCAGGTGGTATTCATGTTTGGCATATGCCTGCTCTGACCGAGATATTTGGAGATGATTCTGTACTT 1200

M. truncatula - CLAW (reversed) CAATTCGGTGGAGGAACTTTAGGACACCCTTGGGGAAATGCACCTGGTGCCGTAGCGAATCGAGTAGCTCTGGAAGCATGTGTACAAGCTCGTAATGAAGGACGTGATCTTGCTCGTGAGGGTAATGAAATTATCCGTGAAGCTACCAAA 1350

M. truncatula - reference CAATTCGGTGGAGGAACTTTAGGACACCCTTGGGGAAATGCACCTGGTGCCGTAGCGAATCGAGTAGCTCTGGAAGCATGTGTACAAGCTCGTAATGAAGGACGTGATCTTGCTCGTGAGGGTAATGAAATTATCCGTGAAGCTACCAAA 1350

M. truncatula - CLAW (reversed) TGGAGTCCTGAATTAGCTGCTGCTTGTGAAGTCTGGAAGGAGATCAAATTTGAATTCCCAGCAATGGATA 1420

M. truncatula - reference TGGAGTCCTGAATTAGCTGCTGCTTGTGAAGTCTGGAAGGAGATCAAATTTGAATTCCCAGCAATGGATA 1420

P. dulcis - CLAW (reversed) ATGTCACCACAAACAGAGACTAAAGCAAGTGTTGGATTCAAAGCTGGTGTTAAAGATTATAAATTGACTTATTATACTCCTGACTATGAAACCAAAGATACTGATATCTTGGCAGCATTTCGAGTAACTCCTCAACCTGGAGTTCCACCT 150

P. dulcis - reference ATGTCACCACAAACAGAGACTAAAGCAAGTGTTGGATTCAAAGCTGGTGTTAAAGATTATAAATTGACTTATTATACTCCTGACTATGAAACCAAAGATACTGATATCTTGGCAGCATTTCGAGTAACTCCTCAACCTGGAGTTCCACCT 150

P. dulcis - CLAW (reversed) GAAGAAGCAGGGGCAGCGGTAGCTGCTGAATCTTCTACTGGTACATGGACAACTGTATGGACTGACGGGCTTACTAGTCTTGATCGTTACAAAGGTCGATGCTACCACATCGAGCCCGTTGCTGGAGAAGAAAGTCAATTTATTGCTTAT 300

P. dulcis - reference GAAGAAGCAGGGGCAGCGGTAGCTGCTGAATCTTCTACTGGTACATGGACAACTGTATGGACTGACGGGCTTACTAGTCTTGATCGTTACAAAGGTCGATGCTACCACATCGAGCCCGTTGCTGGAGAAGAAAGTCAATTTATTGCTTAT 300

P. dulcis - CLAW (reversed) GTAGCTTACCCCTTAGACCTTTTTGAAGAGGGTTCTGTTACTAACATGTTTACTTCCATTGTAGGTAATGTGTTTGGGTTCAAGGCCCTGCGCGCTCTACGTCTGGAGGATTTGCGAATCCCTCCTGCTTATGTTAAAACTTTCCAAGGC 450

P. dulcis - reference GTAGCTTACCCCTTAGACCTTTTTGAAGAGGGTTCTGTTACTAACATGTTTACTTCCATTGTAGGTAATGTGTTTGGGTTCAAGGCCCTGCGCGCTCTACGTCTGGAGGATTTGCGAATCCCTCCTGCTTATGTTAAAACTTTCCAAGGC 450

P. dulcis - CLAW (reversed) CCGCCTCATGGGATCCAAGTTGAGAGAGATAAATTGAACAAGTATGGCCGCCCCCTATTGGGATGTACTATTAAACCTAAATTGGGGTTATCCGCTAAGAATTACGGTAGAGCAGTTTATGAATGTCTCCGCGGTGGACTTGATTTTACC 600

P. dulcis - reference CCGCCTCATGGGATCCAAGTTGAGAGAGATAAATTGAACAAGTATGGCCGCCCCCTATTGGGATGTACTATTAAACCTAAATTGGGGTTATCCGCTAAGAATTACGGTAGAGCAGTTTATGAATGTCTCCGCGGTGGACTTGATTTTACC 600

P. dulcis - CLAW (reversed) AAAGATGATGAGAATGTTAATTCCCAACCATTTATGCGTTGGAGAGACCGTTTCTTATTTTGTGCCGAAGCAATTTATAAAGCACAGGCTGAAACAGGTGAAATCAAAGGGCATTACTTGAACGCTACTGCAGGTACATGCGAAGAGATG 750

P. dulcis - reference AAAGATGATGAGAATGTTAATTCCCAACCATTTATGCGTTGGAGAGACCGTTTCTTATTTTGTGCCGAAGCAATTTATAAAGCACAGGCTGAAACAGGTGAAATCAAAGGGCATTACTTGAACGCTACTGCAGGTACATGCGAAGAGATG 750

P. dulcis - CLAW (reversed) ATCAAAAGAGCTGTATTTGCCAGAGAATTGGGGGTTCCTATCGTAATGCATGATTACTTAACAGGGGGATTCACTGCAAATACTAGCTTGGCTCATTATTGCCGAGATAATGGTTTACTTCTTCACATCCACCGTGCAATGCATGCAGTT 900

P. dulcis - reference ATCAAAAGAGCTGTATTTGCCAGAGAATTGGGGGTTCCTATCGTAATGCATGATTACTTAACAGGGGGATTCACTGCAAATACTAGCTTGGCTCATTATTGCCGAGATAATGGTTTACTTCTTCACATCCACCGTGCAATGCATGCAGTT 900

P. dulcis - CLAW (reversed) ATTGATAGACAGAAGAATCATGGTATGCACTTTCGTGTACTAGCTAAAGCGTTACGTATGTCTGGTGGAGATCATATACACGCTGGTACCGTAGTAGGTAAACTTGAGGGGGAAAGGGAGATCACTTTAGGCTTTGTTGATTTACTACGT 1050

P. dulcis - reference ATTGATAGACAGAAGAATCATGGTATGCACTTTCGTGTACTAGCTAAAGCGTTACGTATGTCTGGTGGAGATCATATACACGCTGGTACCGTAGTAGGTAAACTTGAGGGGGAAAGGGAGATCACTTTAGGCTTTGTTGATTTACTACGT 1050

P. dulcis - CLAW (reversed) GATGATTTTGTTGAAAAAGATCGAAGCCGCGGTATTTATTTCACTCAAGATTGGGTCTCTATGCCAGGTGTTTTGCCTGTAGCTTCAGGGGGTATTCACGTTTGGCATATGCCTGCTCTGACCGAGATCTTTGGAGATGATTCTGTACTA 1200

P. dulcis - reference GATGATTTTGTTGAAAAAGATCGAAGCCGCGGTATTTATTTCACTCAAGATTGGGTCTCTATGCCAGGTGTTTTGCCTGTAGCTTCAGGGGGTATTCACGTTTGGCATATGCCTGCTCTGACCGAGATCTTTGGAGATGATTCTGTACTA 1200

P. dulcis - CLAW (reversed) CAATTTGGCGGCGGAACTTTAGGGCACCCTTGGGGAAATGCACCTGGTGCCGTAGCTAATCGAGTAGCTCTAGAAGCATGTGTACAAGCTCGTAATGAGGGACGTGATCTTGCTCGTGAGGGTAATGAAATTATTCGCGAGGCTAGTAAA 1350

P. dulcis - reference CAATTTGGCGGCGGAACTTTAGGGCACCCTTGGGGAAATGCACCTGGTGCCGTAGCTAATCGAGTAGCTCTAGAAGCATGTGTACAAGCTCGTAATGAGGGACGTGATCTTGCTCGTGAGGGTAATGAAATTATTCGCGAGGCTAGTAAA 1350

P. dulcis - CLAW (reversed) TGGAGTCCTGAACTAGCTGCTGCTTGTGAAATATGGAAGGAGATCAAATTTGAATTCCAAGCAATGGATA 1420

P. dulcis - reference TGGAGTCCTGAACTAGCTGCTGCTTGTGAAATATGGAAGGAGATCAAATTTGAATTCC------------ 1408

V. radiata - CLAW (reversed) ATGTCACCACAAACAGAGACTAAAGCAAGTGTTGGGTTCAAAGCTGGTGTTAAAGATTATAAATTGACTTATTATACTCCTGACTATGAAACCAAAGATACTGATATCTTGGCAGCATTCCGAGTAACTCCTCAACCTGGAGTTCCACCT 150

V. radiata - reference ATGTCACCACAAACAGAGACTAAAGCAAGTGTTGGGTTCAAAGCTGGTGTTAAAGATTATAAATTGACTTATTATACTCCTGACTATGAAACCAAAGATACTGATATCTTGGCAGCATTCCGAGTAACTCCTCAACCTGGAGTTCCACCT 150

V. radiata - CLAW (reversed) GAAGAAGCAGGTGCTGCGGTAGCCGCCGAATCTTCTACTGGTACATGGACAACTGTGTGGACCGATGGGCTTACCAGTCTTGATCGTTACAAAGGACGATGCTATCACATCGAACCTGTTCCTGGGGAAGAAAGTCAATTTATTGCTTAC 300

V. radiata - reference GAAGAAGCAGGTGCTGCGGTAGCCGCCGAATCTTCTACTGGTACATGGACAACTGTGTGGACCGATGGGCTTACCAGTCTTGATCGTTACAAAGGACGATGCTATCACATCGAACCTGTTCCTGGGGAAGAAAGTCAATTTATTGCTTAC 300

V. radiata - CLAW (reversed) GTAGCTTATCCCTTAGACCTTTTTGAAGAAGGTTCTGTTACTAACATGTTTACTTCTATTGTCGGTAATGTATTTGGGTTCAAGGCACTGCGTGCTCTACGTCTGGAGGATTTGCGAATCCCAACCGCTTATGTTAAAACTTTCCAAGGT 450

V. radiata - reference GTAGCTTATCCCTTAGACCTTTTTGAAGAAGGTTCTGTTACTAACATGTTTACTTCTATTGTCGGTAATGTATTTGGGTTCAAGGCACTGCGTGCTCTACGTCTGGAGGATTTGCGAATCCCAACCGCTTATGTTAAAACTTTCCAAGGT 450

V. radiata - CLAW (reversed) CCGCCTCATGGCATCCAAGTTGAGAGAGATAAATTGAACAAGTATGGTCGTCCCCTATTAGGATGTACTATTAAACCTAAATTGGGGTTATCCGCTAAGAATTATGGTAGAGCTGTTTATGAATGTCTTCGTGGGGGACTTGATTTTACC 600

V. radiata - reference CCGCCTCATGGCATCCAAGTTGAGAGAGATAAATTGAACAAGTATGGTCGTCCCCTATTAGGATGTACTATTAAACCTAAATTGGGGTTATCCGCTAAGAATTATGGTAGAGCTGTTTATGAATGTCTTCGTGGGGGACTTGATTTTACC 600

V. radiata - CLAW (reversed) AAAGATGATGAAAATGTGAATTCCCAACCATTTATGCGTTGGAGAGACCGTTTCTTATTTTGTGCTGAAGCGATTTATAAATCACAGGCTGAAACAGGTGAAATCAAAGGGCATTACTTGAATGCAACTGCGGGTACATGCGAAGAAATG 750

V. radiata - reference AAAGATGATGAAAATGTGAATTCCCAACCATTTATGCGTTGGAGAGACCGTTTCTTATTTTGTGCTGAAGCGATTTATAAATCACAGGCTGAAACAGGTGAAATCAAAGGGCATTACTTGAATGCAACTGCGGGTACATGCGAAGAAATG 750

V. radiata - CLAW (reversed) ATGAAAAGAGCTATATTTGCCAGAGAATTAGGTGTTCCTATCATAATGCATGATTATTTAACAGGGGGATTCACTGCAAATACTAGCTTGGCTCATTATTGCCGAGATAATGGTCTACTTCTTCATATACATCGTGCAATGCATGCAGTT 900

V. radiata - reference ATGAAAAGAGCTATATTTGCCAGAGAATTAGGTGTTCCTATCATAATGCATGATTATTTAACAGGGGGATTCACTGCAAATACTAGCTTGGCTCATTATTGCCGAGATAATGGTCTACTTCTTCATATACATCGTGCAATGCATGCAGTT 900

V. radiata - CLAW (reversed) ATCGACAGACAAAAGAATCATGGTATGCACTTTCGTGTATTAGCTAAAGCATTACGTTTATCTGGTGGAGATCATGTCCACTCCGGTACCGTAGTAGGTAAACTTGAAGGGGAAAGAGAAATCACTTTAGGTTTTGTTGACTTACTGCGT 1050

V. radiata - reference ATCGACAGACAAAAGAATCATGGTATGCACTTTCGTGTATTAGCTAAAGCATTACGTTTATCTGGTGGAGATCATGTCCACTCCGGTACCGTAGTAGGTAAACTTGAAGGGGAAAGAGAAATCACTTTAGGTTTTGTTGACTTACTGCGT 1050

V. radiata - CLAW (reversed) GATGATTTTGTTGAAAAAGATCGAAGTCGTGGTATTTATTTCACTCAGGATTGGGTTTCTCTACCAGGTGTTATACCTGTTGCTTCGGGAGGTATTCACGTTTGGCATATGCCTGCTCTGACCGAAATCTTTGGAGATGATTCCGTACTT 1200

V. radiata - reference GATGATTTTGTTGAAAAAGATCGAAGTCGTGGTATTTATTTCACTCAGGATTGGGTTTCTCTACCAGGTGTTATACCTGTTGCTTCGGGAGGTATTCACGTTTGGCATATGCCTGCTCTGACCGAAATCTTTGGAGATGATTCCGTACTT 1200

V. radiata - CLAW (reversed) CAATTTGGCGGAGGAACTTTAGGACACCCTTGGGGGAATGCACCAGGTGCTGTAGCTAATCGAGTAGCTCTTGAAGCATGTGTGAAGGCTCGAAATGAAGGACGTGATCTTGCTCGTGAAGGTAATGAAATTATCCGTGAGGCTAGCAAA 1350

V. radiata - reference CAATTTGGCGGAGGAACTTTAGGACACCCTTGGGGGAATGCACCAGGTGCTGTAGCTAATCGAGTAGCTCTTGAAGCATGTGTGAAGGCTCGAAATGAAGGACGTGATCTTGCTCGTGAAGGTAATGAAATTATCCGTGAGGCTAGCAAA 1350

V. radiata - CLAW (reversed) TGGAGTCCTGAATTAGCTGCTGCTTGCGAAGTATGGAAGGAGATAAAATTTGAATTC 1407

V. radiata - reference TGGAGTCCTGAATTAGCTGCTGCTTGCGAAGTATGGAAGGAGATAAAATTTGAATTC 1407
