# Supplementary material for: CLAW: An automated Snakemake workflow for the assembly of chloroplast genomes from long-read data
Source: PLoS Comput Biol. 2024 Feb 9;20(2):e1011870. doi: 10.1371/journal.pcbi.1011870 (PMC10883564; doi:10.1371/journal.pcbi.1011870)
Supplement: S1 Table — (DOCX) [file pcbi.1011870.s002.docx]

| Taxonomic group | Species | ONT Long read accession no. | ONT reads used as input (Mbp) | Reference chloroplast genome accession no. | Reference chloroplast size (kbp) | Assembly size (kbp) | | | No. contigs | | | Similarity (%) | Time to completion (min) | RAM used  (Gb) |
| --- | --- | --- | --- | --- | --- | --- | --- | --- | --- | --- | --- | --- | --- | --- |
|  |  |  |  |  |  | Chl | Mit | Oth | Chl | Mit | Oth |  |  |  |
| Algae | *Chlamydomonas reinhardtii* | ERR3237140 | 60 | NC_005353 | 204 | 467 | - | 59 | 3 | - | 1 | 98.1 | 7.0 | 8.9 |
| Algae | *Chlorella variabilis* | DRR149372 | 21 | NC_015359 | 124 | 120 | - | - | 1 | - | - | 96.1 | 3.8 | 2.8 |
| Algae | *Ostreococcus tauri* | ERR5421724 | 8.5 | NC_008289 | 72 | 184 | 44 | - | 3 | 1 | - | 98.4 | 1.5 | 1.9 |
| Algae | *Pycnococcus provasolii* | DRR252953 | 12 | NC_012097 | 80 | 80 | 49 | - | 1 | 1 | - | 99.5 | 2.2 | 1.8 |
| Monocot | *Asparagus officinalis* | SRR9643837 | 22 | NC_034777 | 157 | 159 | 10 | - | 1 | 1 | - | 98.7 | 6.7 | 4.2 |
| Monocot | *Deschampsia antarctica* | SRR13908657 | 33 | NC_023533 | 135 | 152 | 26 | 17 | 2 | 1 | 2 | 98.9 | 3.3 | 3.5 |
| Monocot | *Oryza sativa* | DRR196880 | 42 | NC_008155 | 135 | 516 | 37 | - | 6 | 1 | - | 98.5 | 10.4 | 8.5 |
| Monocot | *Spirodela polyrhiza* | SRR11472010 | 67 | NC_015891 | 169 | 512 | 96 | 8 | 2 | 1 | 1 | 99.4 | 11.8 | 10.0 |
| Dicot | *Aquilaria sinensis* | SRR9858982 | 54 | NC_029243 | 160 | 173 | 197 | - | 1 | 1 | - | 98.3 | 6.9 | 8.8 |
| Dicot | *Cannabis sativa* | ERR3850904 | 42 | NC_027223 | 154 | 153 | - | 7 | 1 | - | 1 | 98.6 | 7.2 | 7.3 |
| Dicot | *Corylus avellana* | ERR4852503 | 26 | NC_031855 | 160 | 244 | - | 47 | 5 | - | 2 | 98.5 | 4.1 | 4.7 |
| Dicot | *Eucalyptus polybractea* | SRR8692273 | 36 | NC_022393 | 160 | 159 | - | - | 1 | - | - | 98.8 | 5.0 | 6.2 |
| Dicot | *Gossypium longicalyx* | SRR10377593 | 41 | NC_023216 | 160 | 115 | - | - | 1 | - | - | 97.9 | 3.8 | 5.4 |
| Dicot | *Lathyrus sativus* | ERR3374012 | 67 | NC_014063 | 121 | 121 | 78 | - | 1 | 3 | - | 99.2 | 5.7 | 7.3 |
| Dicot | *Medicago truncatula* | SRR10194526 | 45 | NC_003119 | 124 | 124 | - | - | 1 | - | - | 99 | 12.1 | 8.7 |
| Dicot | *Panax ginseng* | SRR13070229 | 29 | NC_006290 | 156 | 136 | - | 14 | 1 | - | 2 | 99.6 | 3.6 | 4.3 |
| Dicot | *Prunus dulcis* | ERR3430399 | 31 | NC_034696 | 158 | 159 | - | - | 1 | - | - | 98.1 | 7.0 | 7.2 |
| Dicot | *Solanum commersonii* | SRR12407219 | 46 | NC_028069 | 156 | 186 | - | 60 | 1 | - | 2 | 99.4 | 4.5 | 3.6 |
| Dicot | *Vigna radiata* | SRR12549534 | 76 | NC_013843 | 151 | 375 | - | 26 | 2 | - | 1 | 98.5 | 11.1 | 9.8 |

Supplementary Table S1. Information on ONT long reads used as input for *CLAW* and the *Unicycler-*generated chloroplast genome assembly statistics.
