# Supplementary material for: CLAW: An automated Snakemake workflow for the assembly of chloroplast genomes from long-read data
Source: PLoS Comput Biol. 2024 Feb 9;20(2):e1011870. doi: 10.1371/journal.pcbi.1011870 (PMC10883564; doi:10.1371/journal.pcbi.1011870)
Supplement: S2 Table — (DOCX) [file pcbi.1011870.s003.docx]

Supplementary Table S2. Information on PacBio long reads used as input for *CLAW* and the *Flye-*generated chloroplast genome assembly statistics.

| **Taxonomic group** | **Species** | **PacBio Long read accession no.** | **PacBio Long reads used as input (Mbp)** | **Reference chloroplast genome**  **accession no.** | **Reference chloroplast**  **Size (kbp)** | **Assembly size (kbp)** | | **No. contigs** | | **Mean**  **coverage (x)** | | **Similarity** (%) | **Time to completion (min)** | **RAM used (Gb)** |
| --- | --- | --- | --- | --- | --- | --- | --- | --- | --- | --- | --- | --- | --- | --- |
|  |  |  |  |  |  | Chl | Mit | Chl | Mit | Chl | Mit |  |  |  |
| Algae | Chlamydomonas reinhardtii | SRR21973883 | 37.9 | NC_005353 | 204 | 205.9 | - | 1 | - | 172 | - | 99.3 | 19.6 | 4.9 |
| Algae | Chlorella variabilis | DRR316159 | 30.3 | NC_015359 | 124 | 177 | - | 1 | - | 137 | - | 89.8 | 6.1 | 6.2 |
| Algae | Ostreococcus tauri | NA | - | NC_008289 | 72 | - | - | - | - | - | - | - | - | - |
| Algae | Pycnococcus provasolii | ERR8705848 | 10.1 | NC_012097 | 80 | 80.2 | - | 1 | - | 101 | - | 99.5 | 3.1 | 2.0 |
| Monocot | Asparagus officinalis | DRR075367 | 54.1 | NC_034777 | 157 | 130 | - | 2 | - | 153 | - | 99.9 | 10.3 | 8.7 |
| Monocot | Deschampsia antarctica | NA | - | NC_023533 | 135 | - | - | - | - | - | - | - | - | - |
| Monocot | Oryza sativa | ERR11472546 | 51.2 | NC_008155 | 135 | 147 | - | 2 | - | 283 |  | 99.9 | 13.9 | 9.0 |
| Monocot | Spirodela polyrhiza | SRR8517588 | 5.9 | NC_015891 | 169 | 19.1 | - | 2 | - | 203 | - | 99.9 | 5.5 | 2.4 |
| Dicot | Aquilaria sinensis | SRR8892931 | 45.1 | NC_029243 | 160 | 214.8 | 161.9 | 2 | 4 | 162 | 22 | 99.7 | 15.2 | 8.8 |
| Dicot | Cannabis sativa | SRR10189116 | 40.9 | NC_027223 | 154 | 161 | - | 2 | - | 230 | - | 99.9 | 11.6 | 5.2 |
| Dicot | Corylus avellana | NA | - | NC_031855 | 160 | - | - | - | - | - | - | - | - | - |
| Dicot | Eucalyptus polybractea | NA | - | NC_022393 | 160 | - | - | - | - | - | - | - | - | - |
| Dicot | Gossypium longicalyx | SRR6335233 | 51.8 | NC_023216 | 160 | 82.7 | - | 19 | - | 318 | - | 99.8 | 12.8 | 8.7 |
| Dicot | Lathyrus sativus | SRR19732304 | 47.9 | NC_014063 | 121 | 121 | - | 1 | - | 323 | - | 99.9 | 9.6 | 10.7 |
| Dicot | Medicago truncatula | SRR6656266 | 64.6 | NC_003119 | 124 | 124.5 | - | 1 | - | 237 | - | 99.9 | 8.4 | 10.4 |
| Dicot | Panax ginseng | NA | - | NC_006290 | 156 | - | - | - | - | - | - | - | - | - |
| Dicot | Prunus dulcis | SRR16267434 | 4.3 | NC_034696 | 158 | 210 | - | 3 | - | 14 | - | 99.9 | 11.1 | 6.8 |
| Dicot | Solanum commersonii | NA | - | NC_028069 | 156 | - | - | - | - | - | - | - | - | - |
| Dicot | Vigna radiata | SRR9994113 | 28.8 | NC_013843 | 151 | 142.5 | - | 2 | - | 142 | - | 98.9 | 11.1 | 6.9 |
