# Supplementary material for: CLAW: An automated Snakemake workflow for the assembly of chloroplast genomes from long-read data
Source: PLoS Comput Biol. 2024 Feb 9;20(2):e1011870. doi: 10.1371/journal.pcbi.1011870 (PMC10883564; doi:10.1371/journal.pcbi.1011870)
Supplement: S3 Table — (DOCX) [file pcbi.1011870.s004.docx]

Supplementary Table S3. Information on PacBio long reads used as input for *CLAW* and the *Unicycler-*generated chloroplast genome assembly statistics.

| **Taxonomic group** | **Species** | **PacBio Long read accession no.** | **PacBio Long reads used as input**  **(Mbp)** | **Reference chloroplast genome accession no.** | **Reference chloroplast size**  **(kbp)** | **Assembly size (kbp)** | | | **No. contigs** | | | **Similarity**  **(%)** | **Time to completion**  **(min)** | **RAM used**  **(Gb)** |
| --- | --- | --- | --- | --- | --- | --- | --- | --- | --- | --- | --- | --- | --- | --- |
|  |  |  |  |  |  | Chl | Mit | Oth | Chl | Mit | Oth |  |  |  |
| Algae | *Chlamydomonas reinhardtii* | SRR21973883 | 37.9 | NC_005353 | 204 | 206 | - | - | 1 | - | - | 99.2 | 17.5 | 5.1 |
| Algae | *Chlorella variabilis* | DRR316159 | 30.3 | NC_015359 | 124 | 177.9 | - | - | 1 | - | - | 89.6 | 5.7 | 5.2 |
| Algae | *Ostreococcus tauri* | NA | - | NC_008289 | 72 | - | - | - | - | - | - | - | - | - |
| Algae | *Pycnococcus provasolii* | ERR8705848 | 10.1 | NC_012097 | 80 | 80.2 | - | 19.4 | 1 | - | 1 | 99.5 | 1.4 | 2.3 |
| Monocot | *Asparagus officinalis* | DRR075367 | 54.1 | NC_034777 | 157 | 136.7 | - | - | 2 | - | - | 97.9 | 6.6 | 7.3 |
| Monocot | *Deschampsia antarctica* | NA | - | NC_023533 | 135 | - | - | - | - | - | - | - | - | - |
| Monocot | *Oryza sativa* | ERR11472546 | 51.2 | NC_008155 | 135 | 134.9 | 137.4 | - | 1 | 3 | - | 99.3 | 8.8 | 9.0 |
| Monocot | *Spirodela polyrhiza* | SRR8517588 | 5.9 | NC_015891 | 169 | 22.8 | - | - | 2 | - | - | 99.9 | 1.2 | 2.4 |
| Dicot | *Aquilaria sinensis* | SRR8892931 | 45.1 | NC_029243 | 160 | 168.2 | 149.2 | - | 2 | 1 | - | 99.2 | 13.3 | 6.5 |
| Dicot | *Cannabis sativa* | SRR10189116 | 40.9 | NC_027223 | 154 | 154.2 | - | - | 1 | - | - | 99.6 | 9.7 | 4.4 |
| Dicot | *Corylus avellana* | NA | - | NC_031855 | 160 | - | - | - | - | - | - | - | - | - |
| Dicot | *Eucalyptus polybractea* | NA | - | NC_022393 | 160 | - | - | - | - | - | - | - | - | - |
| Dicot | *Gossypium longicalyx* | SRR6335233 | 51.8 | NC_023216 | 160 | 138.9 | - | - | 1 | - | - | 99.1 | 9.5 | 6.5 |
| Dicot | *Lathyrus sativus* | SRR19732304 | 47.9 | NC_014063 | 121 | 127.8 | - | - | 2 | - | - | 99.3 | 9.0 | 8.4 |
| Dicot | *Medicago truncatula* | SRR6656266 | 64.6 | NC_003119 | 124 | 125.8 | - | 11 | 1 | - | 1 | 97.3 | 9.3 | 10.2 |
| Dicot | *Panax ginseng* | NA | - | NC_006290 | 156 | - | - | - | - | - | - | - | - | - |
| Dicot | *Prunus dulcis* | SRR16267434 | 4.3 | NC_034696 | 158 | 158.3 | - | - | 1 | - | - | 99.5 | 11.1 | 5.9 |
| Dicot | *Solanum commersonii* | NA | - | NC_028069 | 156 | - | - |  | - | - |  | - | - | - |
| Dicot | *Vigna radiata* | SRR9994113 | 28.8 | NC_013843 | 151 | 177.1 | - | - | 2 | - | - | 99 | 10.8 | 7.4 |
