# Supplementary figures and images for: CLAW: An automated Snakemake workflow for the assembly of chloroplast genomes from long-read data
Source: PLoS Comput Biol. 2024 Feb 9;20(2):e1011870. doi: 10.1371/journal.pcbi.1011870 (PMC10883564; doi:10.1371/journal.pcbi.1011870)

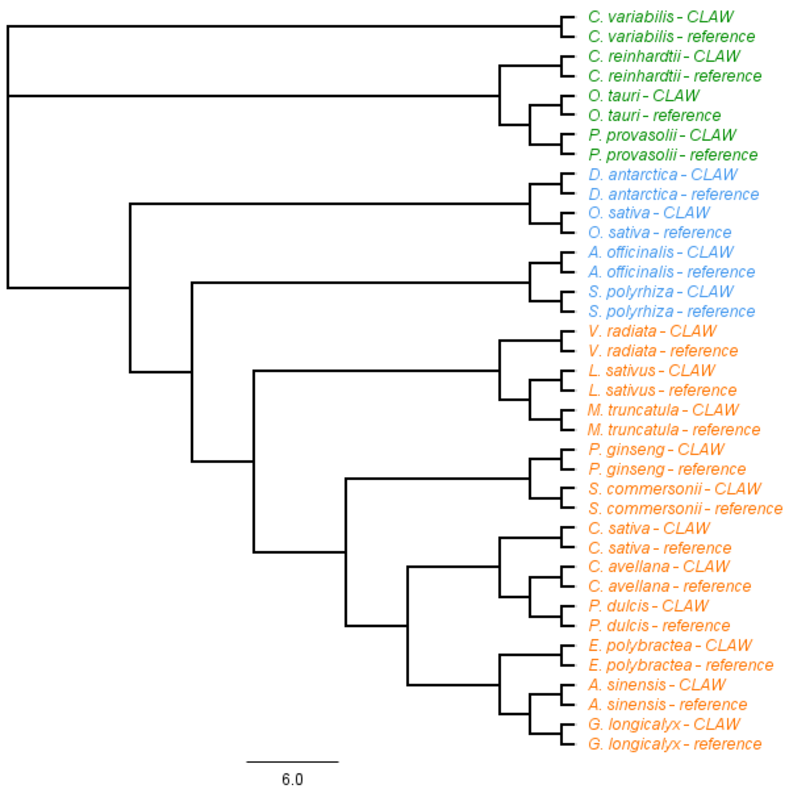

Supplement: S1 Fig — This tree is not meant to infer any phylogenetic relationships. Instead, we include it to show that reference and CLAW-assembled RbcL sequences are similar. Green, blue, and orange highlights indicate algal, monocot, and dicot species, respectively. (TIF) [file pcbi.1011870.s005.tif]
