# Supplementary material for: CLAW: An automated Snakemake workflow for the assembly of chloroplast genomes from long-read data
Source: PLoS Comput Biol. 2024 Feb 9;20(2):e1011870. doi: 10.1371/journal.pcbi.1011870 (PMC10883564; doi:10.1371/journal.pcbi.1011870)
Supplement: Attachment — Submitted filename: Response to Reviewers.docx [file pcbi.1011870.s006.docx]

Dear Editor-in-Chief,

Subject: Resubmission of Manuscript ID PCOMPBIOL-D-23-00916

Enclosed please find the revised version of our manuscript titled "CLAW: An automated Snakemake workflow for the assembly of chloroplast genomes from long-read data" for reconsideration for publication in PLOS Computational Biology.

We are grateful for the invaluable feedback provided by the reviewers and the editorial team during the initial review process. We have meticulously addressed all concerns, comments, and suggestions raised by the reviewers in our revised manuscript. Please see below for a detailed breakdown of responses to reviewer comments.

We believe that the significant revisions have substantially strengthened the manuscript, enhancing its clarity and rigor. We have made significant improvements to the manuscript, our tool, *CLAW*, and have included additional analyses (e.g., inclusion of PacBio test datasets in our analyses) as recommended.

To assist in the review process, we have provided a detailed point-by-point response to each reviewer's comments alongside the revised manuscript.

We are confident that the revised manuscript satisfactorily addresses all concerns raised during this rigorous review and greatly improves the quality and relevance of the study. We hope that our efforts are reflective in the revised version.

We would like to express our gratitude for the opportunity to submit our work to your esteemed journal and for the thorough and insightful feedback provided by the reviewers and editorial team.

Thank you for considering our manuscript for reconsideration. We appreciate your time and consideration.

Sincerely,

Dr. Aaron Phillips

**Reviewer #1:**

The authors developed a tool (CLAW) for automated assembly of chloroplast genomes from long read genomic sequences.

The tool is briefly described and the result of applying it to 19 public data sets is presented.

CLAW appears to be a useful tool, extending the set of available chloroplast assemblers with an option to use long read sequences.

I was able to install the software locally and run it on the provided example dataset.

Furthermore, I could apply it to a different dataset (reads: ERR3638927, reference: NC_035417.1) without problems (no full chloroplast was assembled using my settings, though).

I have some concerns that remain to be addressed by the authors.

**Major concerns:**

- the claim in the abstract (and again in l.136f) "currently no long-read, third generation tools exist" seems to be wrong. I found the recently published article "Plastid Genome Assembly Using Long-read data" presenting the tool ptgaul https://onlinelibrary.wiley.com/doi/10.1111/1755-0998.13787 (preprint published in late 2022)

Thank you for picking up on this. We had finalised the initial manuscript in October 2022. While we did check prior to submission in 2023 for the existence of new tools, we did not come across ptGAUL. We have amended the manuscript to address this, and pointed out the major differences between our tool and ptGAUL.

- l.207: the recommendation to try different random seeds in case of failure to assemble the full chloroplast is a bit surprising to me. Does the random seed only affect the selection of reads for assembly? How seed dependent is success (how often did you observe success on a subsequent try)? How often should the user retry? Might there be a more sophisticated way to select a subset of reads (rather than randomly), that increases the chance for success? One such idea could be uniform coverage across the reference chloroplast.

The random seed is only used in the sub selection of reads from the total chloroplast read pool. In our testing if assembly failed, changing the random seed once, and picking a different read subset, was usually enough to result in a successful assembly. Adding complexity and reducing runtime is something we are currently reluctant to do, especially given that in our testing a single seed change was needed. However, if users request such a feature we can add it in a future update. Uniform read coverage may be one such approach, however we wonder whether the IR regions of the chloroplast genomes might make uniform coverage tricky?

- l.229: percent identity does not take large structural differences into account. Have the results been checked for structural variations, as well? The large size differences mentioned in l.292f seem to indicate, that this could be a problem.

We agree that chloroplast genome assemblies potentially suffer from both per-base accuracy and structural issues. On lines 321 to 338 we discuss how the genome graph (gfa file) can be used by the user to assess the structural completeness of the assembly. We also

checked for structural variations using alignments with the reference chloroplast genomes in Geneious. We present some of these alignments in Figure 2. Other assemblies contained duplicated IR and/or SSC regions either in separate contigs, or included in one contig. For example, the Flye assembly for O. sativa using ONT data produced an assembly ~167 Kbp in size, while the reference sequence is ~135 Kbp in size. BLAST results of the excess ~32 Kbp region not found on the reference sequence reveals it to have 99.4% identity to chloroplast sequence from O. sativa:

Aligning the excess ~32 Kbp region to the reference genome used by CLAW shows that this region is excess IR region sequence (~20 Kbp) and SSC sequence (~12 Kbp):

Additionally, it is important to recognise that many of the reference chloroplast genomes used for seeding and assessment were assembled using short-read technologies. Short-read assembled genomes will struggle to correctly resolve the repeat and inverted repeat. Any differences between our long-read chloroplast genomes and the short-read reference should take this into account.

- How exactly have the 19 test species/datasets been selected? Have any datasets been tested but excluded from the analysis? Have any of them be re-run with different seeds? If so, how often?

We checked the available chloroplast genome assemblies and read libraries on NCBI and selected those that were either commercially important species or biologically (i.e., from a research perspective; e.g., model species) important. No datasets have been tested and excluded.We found it necessary to re-run the workflow for some samples with a different random seed. However, the requirement to do so was uncommon (perhaps 1 out of every 50 assembly attempts) and changing the seed once was enough to remedy failed assemblies.

- Why have no PacBio datasets been used? This leaves the claim that CLAW also works on PacBio data (l.349f) unsupported. Also, the speculation in l.372f could possibly be substantiated, if PacBio datasets were tested, as well.

We agree that this is an oversight of our manuscript that resulted from our high use of ONT data and lack of use of PacBio data. As both ONT and PacBio data is unavailable for the example chloroplast assemblies we have included in the manuscript we have downloaded additional PacBio samples and used CLAW to assemble chloroplast genomes.

There were several reasons for this. First, the thesis that this manuscript was developed from was focussed on using ONT long reads. Thus, during the preparation of the manuscript we tested only ONT long read data. Second, we could not find PacBio data for all of the 19 species that we had initially included as part of the ONT tests of CLAW. Third, we assumed that because we obtained assemblies from ONT data, the PacBio data should also work just fine. We realise that this reason was an oversight now. As such, we have amended the manuscript to include tests of PacBio data for a subset of the original 19 species (i.e., those that have publicly available PacBio data).

- Given the relatively low sequence identity of 98.2% (l.407), compared to 99.9% with short reads (l.366), the possibility of using hybrid approaches with short- and long-read data or using short reads to correct long-reads should be discussed.

This part of the discussion was not derived from our own analyses, rather we made reference to an analysis of interspecific chloroplast genome sequence diversity in Diospyros by Li et al. (reference 38). Thus we are unsure of the connection between these analyses and the use of hybrid approaches for genome assembly in this context, though we agree that a hybrid approach would be useful for resolving systematic errors associated with long read technology. We have added mention of the use of short reads for polishing out per base errors to the discussion.

- l.415: why are you confident in using CLAW to answer questions regarding plastid evolution, given the high divergence due to systematic sequencing errors? This probably distorts phylogenetic analyses, particularly of closely related species or when genomes were sequenced with different technologies.

We base our confidence in the CLAW assemblies on some basic analyses conducted using the RbcL gene as an example. We found that the CLAW generated RbcL genes were highly similar to their reference counterparts, as exemplified in the tree below. Additionally, if users lack confidence in the CLAW chloroplast assembly short-reads can optionally be used to remove any inaccuracies within their sequence - we amended the discussion to reflect this.

**Minor concerns:**

- l.74-81: the author summary contains a number of unproven, subjective or bold claims: existing tools being hard to install (which existing tools are you referring to here), the tool being easy to install and easy to use, the anticipation that barriers for entry to the field of bioinformatics are removed. These claims should be phrased a bit more cautiously, or evidence should be provided.

The manuscript has been amended to reflect this.

- l.130: not necessarily the most frequent, but k-mers within a specific frequency range.

The manuscript has been amended to reflect this.

- l.153f: the default is automatic download from NCBI, but the reference can also be provided manually (documented in the README). Therefore, there is no need to discuss potential problems because of HPC configuration. Also, other concerns, like using an unpublished sequence as reference, do not arise for the reader.

The manuscript has been amended to reflect this.

- l.167: which mapper is used?

The manuscript has been amended with this information.

- l.168: shorter than a user-defined threshold (default: 5 kbp)

The manuscript has been amended to reflect this.

- l.169: why are reads larger than the expected chloroplast genome size excluded? They could contain multiple copies of the circular genome.

We decided to exclude reads larger than the expected chloroplast genome size to minimise contamination with nuclear sequence of chloroplast evolutionary origin. Our concern was that if a nuclear-encoded chloroplast-derived sequence was flanked by a sequence that is not of chloroplastic origin (e.g., transposable elements) then this information could skew the assembly process.

- l.186: in case of a complete circular chloroplast, is the sequence oriented and linearized consistently, e.g. always in order LSC, IR, SSC, IR?

When CLAW rotates the linear chloroplast sequence it does so to match the reference. We do not consider the ordering of the reference genome (LSC, IR, SSC and IR). This rotation is done to allow easier and more accurate comparison of the assembled chloroplast genome to the reference chloroplast genome.

- l.216: why have they been previously downloaded? Downloading them is part of the pipeline.

Because there was an issue executing the reference download following a change in the permission of our HPC. Thus, we had to manually download the reference genomes. We had a section in the manuscript explaining why this may also be necessary, which was removed on request of the reviewer, however this information still exists in the README at the git repository.

- l.251: not shown? Consider adding the alignments as a supplement.

We have added the alignments of RbcL (Reference vs CLAW) from three arbitrarily selected species (one algae, one monocot, and one dicot) as a supplementary file. This file contains alignments of RbcL generated using ONT and PacBio reads. We did not include the pairwise alignments for all 19 species as we do not feel that enough information is gained from being able to view all alignments to warrant inclusion.

- l.299f: how much difference has been observed in the IR regions? A circular assembly graph is also possible in case of identical IRs, e.g. if the arrangement of LSC and SSC could be resolved with spanning reads. (Same comment for l.319)

The manuscript has been corrected. Detailed analysis of individual chloroplast assemblies is the responsibility of the users of CLAW. The resulting assemblies are due to the reads parsed into CLAW and thus we cannot account for different outcomes.

- l.352f: comparison of computation times on different hardware are problematic, this fact should be acknowledged.

The manuscript has been amended to reflect this.

- l.363: how was completeness defined and tested?

Completeness was assessed by comparing the length of the assembly with the reference, by investigating CLAW assembly vs reference assembly alignment plots, and through the annotation of the CLAW assemblies using a chloroplast cDNA library.

- consider deposition of the source code at Zenodo in addition to GitHub (so the software is archived and receives a dedicated doi).

Thank you for this suggestion. We will consider this in due course.

- in the installation instructions in the README "cd long-read-chloroplast-assembly" should be replaced by "cd CLAW".

Thank you for picking up on this error. The README has been amended to reflect this.

- consider replacing "--profile profiles/slurm" with "--cores 4" in the instructions, this should work on most systems, while the slurm profile requires a specific setup.

We have amended the README at the git repository to reflect the fact that not all users will have access to a Slurm HPC.

**Reviewer #2:**

The authors present a pipeline to assemble chloroplast genomes using long reads. Their approach involves capturing reads from whole-genome sequencing projects and assembling them using two different tools. Additionally by making use of the snakemake workflow the steps are reproducible and easy to automate.

The manuscript is well written and easy to follow, however I have some concerns. The main one is that there is no comparison regarding performance with ptGAUL (Zhou et al, 2023) given that their methodology is similar. I would like to see a comparison of the performance of both tools and advantages and disadvantages of using each (advantage of snakemake workflow, flye vs flye & unicycler, circularization of the reference, polishing, etc).

Thank you for picking up on this. We had finalised the initial manuscript in October 2022, so had no knowledge of ptGAUL at that time. While we did check prior to submission in 2023 for the existence of new tools, we somehow did not come across ptGAUL. Possibly because we searched for “chloroplast” rather than “plastid”. We do not think a comparison in performance between the two tools is necessary, as this is not the purpose of the current manuscript. However, we tested ptGAUL using the test data set accompanying CLAW and obtained the following results upon alignment of ptGAUL with the reference genome:

ptGAUL completed rapidly, but did not produce a good assembly for our test dataset.

We have added a section outlining the key differences between ptGAUL and CLAW - this section focuses on the advantages of implementing CLAW as a snakemake workflow.

We have added some text to the manuscript about the advantages of polishing with short reads post-assembly, should the user want to.

Chloroplast circularisation was added as a method to increase the alignment rate of true chloroplast sequences, not nuclear reads containing chloroplast genes. If chloroplast assembly fails the user can optionally filter reads based on alignment score. Aligned nuclear sequences should achieve lower mapping scores than true chloroplast sequences. This functionality was recently added.

Additionally I have some other questions:

1) Why use Unicycler? It seems that in your tests Flye gave better results. Do you have a hypothesis as to why more mitochondrial contigs are obtained using Unicycler?

Unicycler was implemented in CLAW to give users more options, should they require it. Users do not have to use Unicycler, but they have the option to use it if they like. Flye and Unicycler use different algorithms for assembly. Sometimes Flye outperforms Unicycler and vice versa. We have no specific hypothesis as to why Unicycler might generate more mitochondrial contigs than Flye. Additionally, by creating CLAW with Snakemake we can easily add additional assemblers to CLAW if any become available.

2) For Figure 2 I would suggest adding the blue, red and green arrows to all 4 subfigures and not only in the first one to make them more comparable.

This change has been implemented.

3) Was a test ran using the same individual for reference and reads? In this case obtaining a 100% identity was possible?

No, this was not attempted.

4) Regarding the github describing the tool, while I find it well explained I believe it can be improved to make it more user friendly for non specialized users. Additionally including a snakemake profile for workstations (or other non cluster configurations) might make the tool more accesible and easy to use for users without much experience with snakemake.

We have amended the README at the git repository to contain instructions on a more generic usage that will work across local and non-Slurm HPC infrastructures. We have added new profiles for local and other HPC infrastructures. However, the authors don't have access to the whole suit of HPC queueing systems and can only test on available systems.

**Reviewer #3:**

Phillips et al. have developed an automated workflow to help assemble chloroplasts from whole genome shotgun long read sequencing data. The workflow includes baiting reads and assembly either with Unicycler or Flye. The authors also describe potential issues that users should be aware of, such as the higher sequencing error rate using nanopore reads, and how inverted repeats, etc. can affect the assemblies and be seen in visualization of the assembly graphs via Bandage.

In general, I found the manuscript to be well-written and a good effort to fill in a need to assemble chloroplast genomes with long read sequencing data. There are various tools (as the authors cite) for short read data assemblies, but chloroplasts provide a particular challenge with genomic inversions and long read data can help with these issues. I think this study is a valuable contribution to the field.

**Major comments:**

1. The manuscript would benefit from a figure outlining the workflow in a graphical format.

We have added the following graphical workflow.

2. The workflow only supports use on a cluster. There are still many labs that do not use clusters but run bioinformatics tools on their own servers and do not need a workflow that accommodates SLURM scheduling. Please include an alternative workflow such as this – it will help improve the reach of this tool and also encourage novice users as the authors state in the Author Summary section.

CLAW can be executed on the users personal device, locally, with no need to access a HPC or other cloud computing infrastructure. We have amended the manuscript to make this more clear, and have added more details to the README at the git repository on how this can be done using the newly added –profile profiles/local option.

3. The manuscript would benefit from including PacBio test data for a few samples, or attempting to use a tool like Medaka to error correct. I appreciate that the authors point out the issues with the error rate in nanopore data, so it is unclear why they did not attempt to use PacBio data as well, or use medaka or to attempt to error correct their assembled genomes. Please explain or provide further analysis.

We have provided some additional analyses, this time including PacBio reads for a subset of the species we initially reported on.

**Minor comments:**

1. Amend references to plants to at least algae, plants and other photosynthetic organisms. In certain places in the manuscript, the authors describe chloroplasts as being from plant cells or describe CLAW in respect to plants. Technically algae are not considered plants, such as diatoms and euglena, that have chloroplasts. Cyanobacteria also have chloroplasts. CLAW would be useful for assembling chloroplasts from these organisms as well. Please amend language in the manuscript to reflect this (e.g. line 42, 80)

We have added reference to algal cells in the manuscript. We do not accept that cyanobacteria contain chloroplasts, rather that cyanobacteria are the ancestors of chloroplasts in algal and plant cells. Thus, we have not amended our language to cover this facet of the review.

2. Minimap2 is not stated as the method for read baiting and default parameters are not described in the methods overview. Please include in the methods section.

We have added this information.

3. Line 149: authors mention fast5, POD5 is now the default and should also be mentioned

We have added mention of POD5 to this section.

4. Line 292: Were all of the reference genomes circular? Illumina only genomes tend to be smaller than complete, circular genomes.

According to NCBI, all reference genomes were circular except for *Medicago truncatula*, which is reported as a linear assembly.

5. Error on the github installation directions, need to cd into CLAW, not “long-read-chloroplast-assembly”

Thank you for this correction.
