# Supplementary material for: CLAW: An automated Snakemake workflow for the assembly of chloroplast genomes from long-read data
Source: PLoS Comput Biol. 2024 Feb 9;20(2):e1011870. doi: 10.1371/journal.pcbi.1011870 (PMC10883564; doi:10.1371/journal.pcbi.1011870)
Supplement: Attachment — Submitted filename: Response_to_reviewer_comments.docx [file pcbi.1011870.s007.docx]

Reviewer #1: The authors updated the manuscript, addressing most of the concerns raised by the reviewers. Importantly, the related published tool ptGAUL is now mentioned. Overall, the manuscript is hugely improved. My remaining concerns are:
- Evaluation of the completeness and accuracy of chloroplast genome assemblies is challenging, so thanks to the authors for providing some further detail on differences between CLAW assemblies and the reference. As mentioned in l.317ff, there are on average differences of more than 15kbp between assembly and reference. Even if some of the difference is due to an additional or missing copy of the IR, I would consider this inaccurate or incomplete. Thus, I don't think the claim in line 405, that CLAW was able to "assemble complete and accurate" genomes for all test datasets, is fully justified.

The manuscript has been updated to reflect this.

- Thanks for providing some RbcL alignments. However, all pairwise alignments should be shown, rather than a manual selection. Providing them all is easier to explain and does not raise questions about the selection procedure. Also, if they are provided in a suitable format (e.g. a zip of fasta files), the file size will be neglectible.

We have updated the manuscript to reflect this.

Reviewer #2: The new version of the submitted manuscript is greatly improved and I also believe that the addition of the diagram regarding the pipeline was an important addition. The authors have addressed all the issues and answered all the questions that I had.

Reviewer #3: I am mostly satisfied with the authors edits. I have two requests for minor revisions:

1. Change the last line (477) “Users may consider the use of short read technology, if available, to correct errors in their assemblies” to “Users may consider the use of short read technology, if available, or nanopore reads, if there was enough sequencing depth, to correct errors in their assemblies.” Or something similar. The reference cited also mentions Medaka, which uses nanopore reads to polish the nanopore assemblies, not just short read polishers. This also points out to the read that they may not need to generate another dataset for polishing.

The manuscript has been updated to reflect this.

2. Move the edited sentence from #1 up to the bottom of the paragraph describing the error rates that ends on line 434. I find that users new to ONT and PacBio are often unaware of the ability to polish assemblies, so moving that sentence and reference closer to the discussion I think will help reduce confusion for those readers.
The manuscript has been updated to reflect this.

Other than those two revisions, I recommend this manuscript for publication. An aside is that with the new Dorado basecaller from nanopore, polishing the error may soon be unnecessary with the 10.4.1 chemistry.

The authors were right to correct my comment about cyanobacteria and chloroplasts. That was a misunderstanding of the biology on my part
